# Zebrafish vascular quantification: a tool for quantification of three-dimensional zebrafish cerebrovascular architecture by automated image analysis

**DOI:** 10.1242/dev.199720

**Published:** 2022-02-14

**Authors:** Elisabeth C. Kugler, James Frost, Vishmi Silva, Karen Plant, Karishma Chhabria, Tim J. A. Chico, Paul A. Armitage

**Affiliations:** 1 Department of Infection, Immunity and Cardiovascular Disease, University of Sheffield, Medical School, Beech Hill Road, Sheffield S10 2RX, UK; 2 The Bateson Centre, Firth Court, University of Sheffield, Western Bank, Sheffield S10 2TN, UK; 3 Insigneo Institute for in silico Medicine, The Pam Liversidge Building, Sheffield S1 3JD, UK; 4 Hull York Medical School, John Hughlings Jackson Building, University Road, University of York, Heslington, York YO10 5DD, UK

**Keywords:** Angiogenesis, LSFM, Registration, Quantification, Vasculature

## Abstract

Zebrafish transgenic lines and light sheet fluorescence microscopy allow in-depth insights into three-dimensional vascular development *in vivo*. However, quantification of the zebrafish cerebral vasculature in 3D remains highly challenging. Here, we describe and test an image analysis workflow for 3D quantification of the total or regional zebrafish brain vasculature, called zebrafish vasculature quantification (ZVQ). It provides the first landmark- or object-based vascular inter-sample registration of the zebrafish cerebral vasculature, producing population average maps allowing rapid assessment of intra- and inter-group vascular anatomy. ZVQ also extracts a range of quantitative vascular parameters from a user-specified region of interest, including volume, surface area, density, branching points, length, radius and complexity. Application of ZVQ to 13 experimental conditions, including embryonic development, pharmacological manipulations and morpholino-induced gene knockdown, shows that ZVQ is robust, allows extraction of biologically relevant information and quantification of vascular alteration, and can provide novel insights into vascular biology. To allow dissemination, the code for quantification, a graphical user interface and workflow documentation are provided. Together, ZVQ provides the first open-source quantitative approach to assess the 3D cerebrovascular architecture in zebrafish.

## Abstract (precis)

**Summary:** An image analysis workflow pipeline for 3D quantification of the total or regional zebrafish brain vasculature, called zebrafish vasculature quantification or ZVQ.

## INTRODUCTION

Our understanding of the mechanisms governing vascular development relies heavily on animal models. The zebrafish is an excellent model for three dimensional (3D) *in vivo* vascular imaging (Huisken et al., 2004; Gut et al., 2017) due to advantages such as optical clarity of the embryo, multiple vascular transgenic lines (Chávez et al., 2016; Chico et al., 2008) and advanced microscopy, including light sheet microscopy (LSFM) that can visualise the entire vasculature. Despite its small size, the zebrafish embryo possesses a complex vascular architecture that expands rapidly over time. Some vascular territories, such as the trunk, display a simple and highly stereotyped pattern, but regions such as the cerebral vessels are far more complex, with greater inter-individual and inter-group variability.

Although acquisition of high-resolution imaging of the vasculature in zebrafish and other preclinical models has advanced considerably, objective quantification remains limited. Most studies rely on subjective visual assessment to identify whether an experimental manipulation affects vascular development. If such effects are detected ‘by eye’, quantification may be performed manually on selected vessels, but this biases towards detection of obvious phenotypes. Furthermore, manual quantification of even a small number of vessels in a single embryo is time-consuming, and does not quantify vascular anatomy elsewhere. Vascular phenotypes are commonly reported using representative images of individual animals, rather than comprehensively reporting inter-individual and inter-group variability.

A robust automated approach that co-registers individuals to display a ‘map’ of regions of vascular similarity and variability, and to quantify 3D vascular anatomy would substantially address these limitations. However, to achieve this, many challenges need to be considered. LSFM datasets are often large, rendering data handling, processing and analysis computationally demanding. The cerebral vessels of a 3 dpf zebrafish measure ∼600 µm laterally and the dorsal cerebral vasculature measures ∼280 µm axially. Vessel diameters typically range from 5 to 20 µm, showing either a double-peak (lumenised) or single-peak (unlumenised) cross-sectional intensity distribution with ∼1 µm thick vessel walls (Kugler et al., 2018, 2019a, 2020a). Thus, to provide sufficient information, images are acquired with ∼0.33×0.33×0.6 µm (*x*,*y*,*z*) voxel size and 1920×1920×460 voxels, resulting in images of about 3.5 GB per embryo. File size increases exponentially with additional fluorophores/channels, time-points, experiments, views, etc.

Analysis of preclinical imaging data often lacks assessment of data properties, such as noise, artefacts and motion. A further challenge is that typically only 3-15% of voxels in vascular imaging datasets are vascular, limiting the information available to drive analysis approaches and making processing potentially prone to bias towards background, rather than vascular information (Haixiang et al., 2017; Rahman and Davis, 2013; Tetteh et al., 2019). These issues have led most quantitative studies to analyse 2D projections, an approach that fails to capture the complexity of the 3D vasculature and is confounded by overlying vessels. 3D quantification is clearly preferable, but is technically and computationally more challenging.

Very few previous studies have attempted to quantitatively characterise the zebrafish cranial vasculature. Tam et al. (2012) and Chen et al. (2012) focussed on characterising small vascular regions, instead of the entirety of the brain vasculature, in confocal microscopy images using commercial software. We previously presented a 3D enhancement and segmentation approach for the cranial vasculature in zebrafish transgenic reporter lines (Kugler et al., 2018, 2019a). Daetwyler et al. (2019) presented a method to segment the zebrafish cerebral vasculature using machine learning (dual ResUNet) but did not include further quantification.

Accurate segmentation is the foundation of subsequent analysis and quantification. We therefore previously developed and validated a zebrafish vascular segmentation workflow and examined its sensitivity, robustness and accuracy (Kugler et al., 2020a,b preprint). We applied this to the transgenic *Tg(kdrl:HRAS-mCherry)^s916^* (Chi et al., 2008) using Sato enhancement and Otsu thresholding (Kugler et al., 2019a; Sato et al., 1998; Otsu, 1979). Our segmentation approach handles lumenised (double-peak cross-sectional intensity distribution) and unlumenised (single-peak cross-sectional intensity distribution) vessels by conversion of double peaks to single peaks, artificially ‘filling’ all vessels, allowing vascular segmentation independent of lumenisation. We confirmed the robustness of our approach with respect to fluorescence signal, sensitivity to true biological differences and ability to extract vessels at different scales.

Building on this validated segmentation approach, we now present the first open-source 3D image analysis pipeline for zebrafish vasculature, called ‘ZVQ’. We apply this to the cerebral vasculature and show that it reliably provides: (1) registration of multiple embryos, allowing examination of vascular patterning similarity/variability between individuals or groups; and (2) global and/or regional quantification of eight vascular parameters (similarity, volume, surface area, density, branching points, length, radius and complexity).

To demonstrate the scientific utility and throughput of our tools, we applied these to datasets from 13 different experiments and quantified the following: (1) changes in vascular architecture during embryonic development from 2 to 5 dpf; (2) the effect of absent blood flow (Sehnert et al., 2002); (3) pharmacological manipulations of components associated with angiogenesis, vasoconstriction and endothelial cell (EC) architecture [i.e. vascular endothelial growth factor (VEGF) inhibition (Nakamura et al., 2006), Notch inhibition (Geling et al., 2002), F-actin polymerisation inhibition (Morton et al., 2000), myosin II inhibition (Kovács et al., 2004), changed osmotic pressure by glucose increase, and altered membrane rigidity by DMSO treatment; (4) the effect of previously published morpholino antisense oligonucleotides targeting *jagged1a* (*jag1a*) (Geudens et al., 2010), *jagged1b* (*jag1b*) (Geudens et al., 2010), *dll4* (Geudens et al., 2010), *notch1b* (Quillien et al., 2014) and collagen- and calcium-binding EGF domain-containing protein 1 (*ccbe1*) (Guen et al., 2014); (5) regional vascular topology of the mid- versus hindbrain; and (6) left-right vascular symmetry.

To allow dissemination and wider application of our method, we implemented it as a workflow in the open-source image analysis software Fiji (Schindelin et al., 2012), which contains a custom graphical user interface (GUI), accompanied with comprehensive workflow documentation. In summary, we describe the first comprehensive 3D quantification approach for zebrafish cerebrovascular quantification that generates a far larger quantity of data than manual analysis in a fraction of the time. It detects quantifiable differences of a magnitude that is difficult or impossible to detect by eye, and provides a range of novel tools to analyse and present these data. These tools are likely to aid discovery of biological insights into vascular development.

## RESULTS

### Establishment of the ZVQ workflow for vascular patterning analysis and quantification

**Fig. 1.**
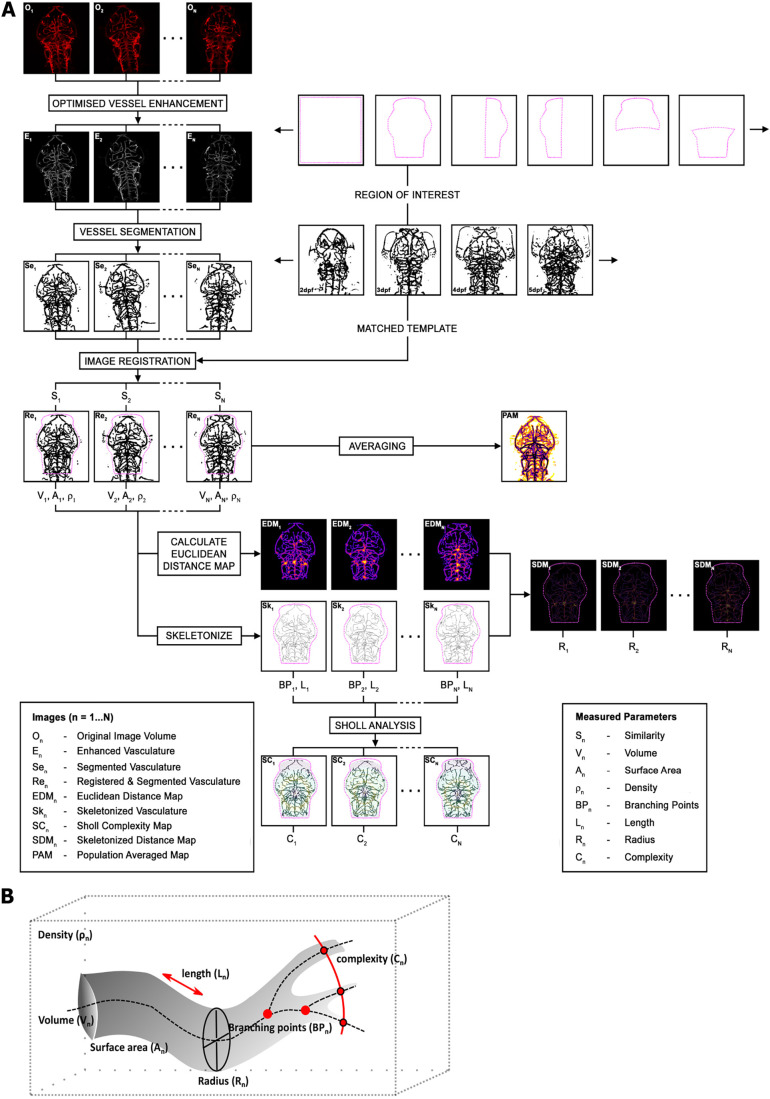
**ZVQ workflow overview.** (A) Workflow of ZVQ. First, the original images (O_n_) are motion corrected, enhanced (E_n_) and segmented (Se_n_). Registration to template embryo brings all embryos into one spatial coordinate system (Re_n_), allowing quantification of similarity (S_n_), and is followed by the generation of a population average map (PAM). For examination of specific regions of vascular development, a region of interest (ROI) can be specified. Following ROI extraction, volume (V_n_), surface voxel (A_n_) and vascular density (ρ_n_) are quantified. Euclidean distance maps (EDM_n_) are combined with vascular skeletons (Sk_n_) to quantify radius in skeletonised distance maps (SDM_n_ and R_n_), which are also used to quantify network length (L_n_), branching points (BP_n_) and complexity (C_n_) via Sholl analysis. (B) Schematic of the vascular parameters extracted by ZVQ.

We established the ZVQ workflow in the open-source image analysis framework, Fiji (Schindelin et al., 2012) (Fig. 1A). Briefly, ZVQ builds on our published optimised segmentation approach using Sato enhancement and Otsu thresholding (Kugler et al., 2019a; Sato et al., 1998; Otsu, 1979) that established the first validated segmentation approach for the zebrafish vasculature. This was applied to all data presented to extract the vasculature.

After segmentation, individual embryos are registered to a template animal (see details below). A user-defined region of interest (ROI) can be specified for analysis of specific regions. Following segmentation and registration, to examine patterning differences between animals or groups of animals, a single population average map (PAM) is generated from all animals in a specified group (Fig. 1A). This PAM shows areas of reproducible and variable vascular patterning within this group. Comparison of the PAMs between experimental groups allows assessment of regional vascular patterning differences (specific examples are shown below).

Following generation of the PAMs, automated quantification of a range of vascular parameters was performed (Fig. 1B). These are: (1) vascular similarity (S_n_) – the volume of vascular overlap quantified by Dice coefficient (a measure of inter-animal variability in vascular anatomy in the specified ROI); (2) vascular volume (V_n_) – vascular voxels classified using segmentation as vascular and non-vascular; (3) surface area (A_n_); (4) density (ρ_n_) – the ratio of vascular volume to total volume of interest; (5) network length (L_n_) – the number of centre-line voxels, derived by 3D thinning of the segmented vasculature; (6) branching points (BP_n_) – points where vessels split up into daughter branches; (7) average radius (R_n_) – vascular thickness given by the distance from a local centre-line (or vessel radius midpoint) to corresponding vessel walls; and (8) complexity (C_n_) – number of concentric shell intersections from the centre point using Sholl analysis, where the subscript *n* represents a measurement in the *n*^th^ fish of the group under study (Fig. 1; Fig. S1).

Once established, we applied ZVQ to a range of experimental datasets to examine its performance in visualising and quantifying biological differences between groups of embryos. ZVQ builds on our optimised segmentation approach, using Sato enhancement and Otsu thresholding (Kugler et al., 2019a; Sato et al., 1998; Otsu, 1979), that presents the first fully validated segmentation approach for the zebrafish vasculature, and was used for all data in this article.

### Applying ZVQ to quantify cerebral vascular development from 2 to 5 dpf

**Fig. 2.**
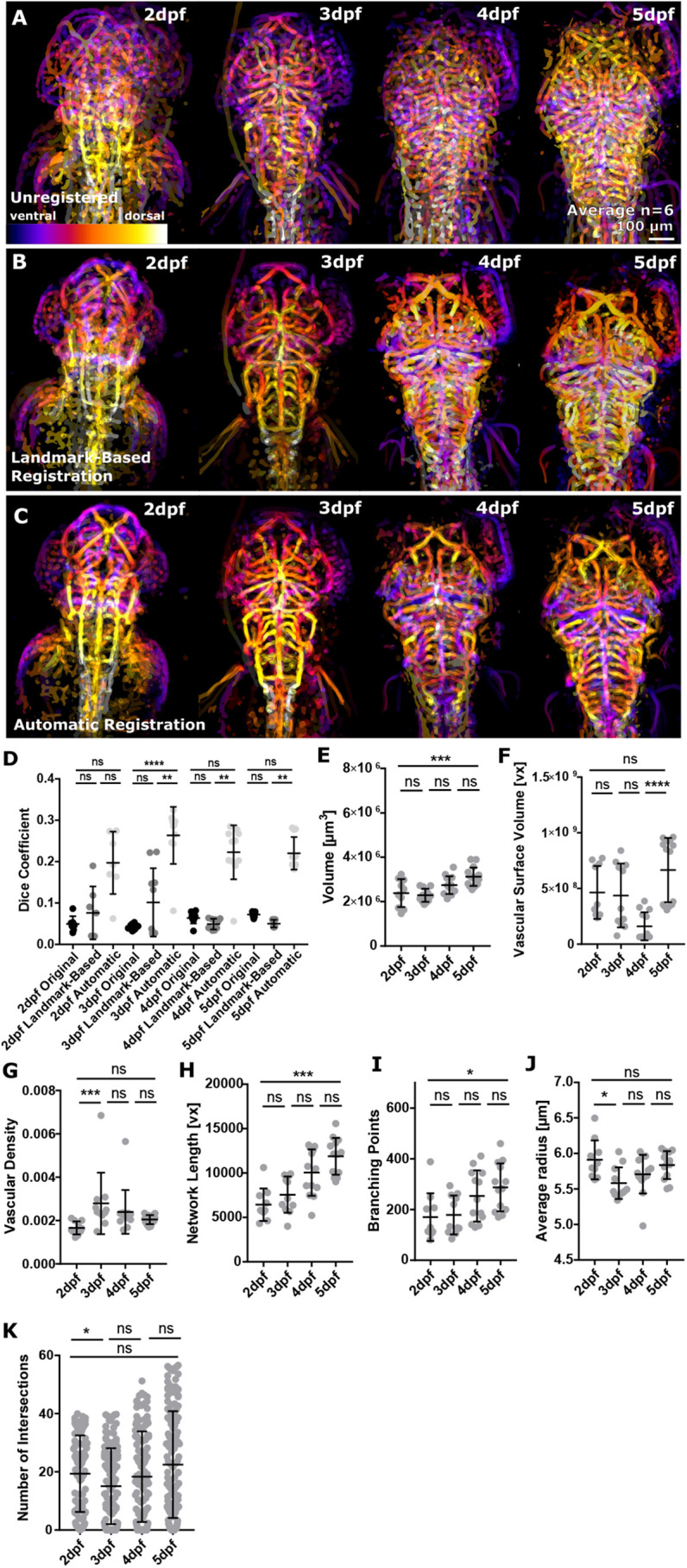
**Application of ZVQ to embryos from 2 to 5 dpf to analyse vascular growth.** (A) Depth-coded MIP showing regions of overlap (purple, ventral; white, dorsal) of six fish before registration from 2 to 5 dpf. (B) Depth-coded MIP showing regions of overlap (purple, ventral; white, dorsal) of six fish after manual registration from 2 to 5 dpf. (C) Depth-coded MIP showing regions of overlap (purple, ventral; white, dorsal) of six fish after automatic registration from 2 to 5 dpf. (D) Dice coefficient between template and moving image was increased after application of rigid registration using both anatomical landmark-based and automatic rigid registration from 2 to 5 dpf [2 dpf, *n*=7; 3 dpf, *n*=10; 4 dpf, *n*=10; 5 dpf, *n*=10; two experimental repeats; Kruskal–Wallis test; ns (*P*>0.5), **P=0.01-0.001, ****P<0.0001; data are mean±s.d.]. (E) Vascular volume was statistically significantly increased from 2 to 5 dpf (*P*=0.0008; 2 dpf, *n*=10; 3 dpf, *n*=12; 4 dpf, *n*=13; 5 dpf, *n*=15; one-way ANOVA; data are mean±s.d.). (F) Vascular surface was not statistically significantly increased from 2 to 5 dpf (*P*=0.4885; 2 dpf, *n*=10; 3 dpf, *n*=11; 4 dpf, *n*=13; 5 dpf, *n*=14; two experimental repeats; Kruskal–Wallis test; data are mean±s.d.). (G) Vascular density was not statistically significantly increased from 2 to 5 dpf (*P*=0.2041; 2 dpf, *n*=10; 3 dpf, *n*=11; 4 dpf, *n*=13; 5 dpf, *n*=14; two experimental repeats; Kruskal–Wallis test; data are mean±s.d.). (H) Network length was statistically significantly increased from 2 to 5 dpf (*P*=0.0001; 2 dpf, *n*=10; 3 dpf, *n*=12; 4 dpf, *n*=13; 5 dpf, *n*=15; Kruskal–Wallis test; mean±s.d.). (I) Branching points were statistically significantly increased from 2 to 5 dpf (*P*=0.0082; Kruskal–Wallis test; data are mean±s.d.). (J) Average vessel radius was not statistically significantly changed from 2 to 5 dpf (*P>0.9999; Kruskal–Wallis test; data are mean±s.d.). (K) Sholl analysis was conducted to assess vascular complexity, showing no significant increase from 2 to 5 dpf (2-3 dpf, *P=0.0340; 3-4 dpf, *P*=0.6825; 4-5 dpf *P*=0.2000; 2-5 dpf, *n*=5; Kruskal–Wallis test; data are mean±s.d.).

We used ZVQ to examine changes in cerebral vascular development between 2 and 5 dpf. We first used both manual rigid landmark-based and automatic rigid registration methods to co-register 2, 3, 4 and 5 dpf embryos (six per group, registering five individuals to a single target embryo) (Fig. 2A-C; Figs S2, S3), to generate population average maps (PAMs) of these groups. Examination of these PAMs allows identification of regions of similarity and variability within the group (Fig. S3C-G; Movies 1-4). Quantification of Dice score showed higher inter-group similarity after automatic registration (Fig. 2D; 2 dpf manual *P*>0.9999, automatic *P*=0.2154; 3 dpf manual *P*>0.9999, automatic *P*<0.0001; 4 dpf manual *P*>0.9999, automatic *P*=0.3962; 5 dpf manual *P*>0.9999, automatic *P*>0.9999) and a lower coefficient of variation (CoV) after automatic registration (2 dpf, 38.18%; 3 dpf, 26.21%; 4 dpf, 29.23%; 5 dpf, 17.93%) than manual registration (2 dpf, 84.02%; 3 dpf, 81.20%; 4 dpf, 26.21%; 5 dpf, 18.39%).

Unlike examination of individual animals, examining PAMs of groups from 2 to 5 dpf allows identification of regions of conserved vascular patterning. Fig. 2C shows PAMs from six animals each at 2, 3, 4 and 5 dpf. These demonstrate a high degree of vascular similarity at all time points, particularly of perineural vessels, such as the primordial midbrain channel (PMBC), primordial hindbrain channel (PHBC), middle cerebral vein (MCeV), mesencephalic vein (MsV) and central arteries (CtAs). Over time, the expansion of the hindbrain vasculature between 2 and 5 dpf, with an increase in the number of central arteries, could be observed.

We next examined the ability of ZVQ to detect vascular topology parameters, which were expected to increase in volume, length, branching points and complexity in the ROI of the entire brain over 3 days of embryonic development. We found that vascular volume (Fig. 2E; *P*=0.0008; one-way ANOVA), network length (Fig. 2H; *P*<0.0001; Kruskal-Wallis test) and branching points (Fig. 2I; *P*=0.0082; Kruskal-Wallis test), but not surface volume (Fig. 2F; *P*=0.4885), density (Fig. 2G; *P*=0.2041), average radius (Fig. 2J; *P*>0.9999; Kruskal-Wallis test) or complexity (Fig. 2K; *P*>0.9999; Kruskal-Wallis test), significantly increased from 2 to 5 dpf. This suggested quantification by ZVQ is able to detect biologically meaningful changes in vascular parameters with statistical significance, even in relatively low group sizes of six embryos.

Together, this shows for the first time that landmark- and object-based rigid inter-sample registration can be used to bring the vasculature of different embryos into one spatial coordinate system, with automatic registration performing better than manual landmark-based registration. This ability to register multiple embryos allows the consistent automated placement of regions of interest and produces a PAM that allows inter-group conservation of vascular patterning to be assessed. Furthermore, ZVQ rapidly provides quantitation of multiple parameters that show statistically significant changes during early embryonic development in a manner many times faster than manual quantification.

### Examining the impact of inter-sample registration on measured vascular parameters

Our workflow was implemented to allow quantification either without registration (requiring manual ROI selection for each individual fish) or with registration (using one ROI selection per group). We therefore compared the performance of our quantification using both non-registered and registered datasets. This showed good agreement of data before and after registration (Fig. S4A-F), with a reduction in coefficient of variation (CoV) of volume (before registration, 11.14%; after registration, 5.98%), length (before, 12.03%; after, 3.56%) and branching points (before, 14.78%; after, 6.21%), but not surface (before, 5.96%; after, 6.02%), density (before, 3.80%; after, 7.57%) or diameter (before, 1.89%; after, 2.02%).

This suggests that registration results in some minor alteration of the quantified parameters. Conversely, quantification after registration reduces manual work by allowing one ROI per group to be applied to all samples rather than specification of the ROI for each individual.

As 3D registration of the zebrafish brain vasculature has never been performed before, we examined which similarity measurement might be most appropriate to assess registration outcomes in our data. We quantified Jaccard index, Dice coefficient, total overlap, mutual information, sum of squared differences, mean square error and structural similarity, finding similar outcomes in all metrics (Fig. S4G-M).

### Using ZVQ to quantify the effect of experimental manipulations on cerebral vascular patterning

**Fig. 3.**
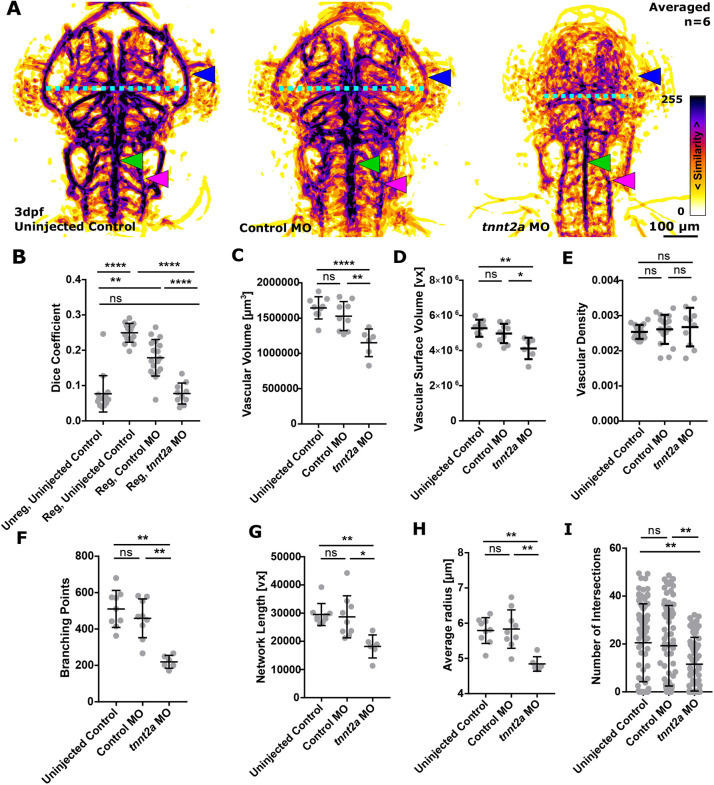
**Absent blood flow reduces the cerebral vasculature.** (A) MIPs of averaged data of six uninjected controls, control MO and *tnnt2a* MO following segmentation and registration, showing high inter-embryo similarity in midbrain vessel patterning [PMBC pattern (blue arrowhead), head size (PMBC′ to PMBC″ distance; cyan dotted line), BA (green arrowhead) and PHBC (magenta arrowhead)]. (B) A statistically significant decrease in the Dice coefficient was found when comparing registered control MO to *tnnt2a* MO (****P<0.0001; uninjected control=15, control MO=18, *tnnt2a* MO=10; Kruskal–Wallis test; data are mean±s.d.). (C) Vascular volume was statistically significantly decreased in *tnnt2a* MO (uninjected control, ****P<0.0001; control MO, ****P<0.0001; uninjected control=9, control MO=9, *tnnt2a* MO=6; one-way ANOVA; data are mean±s.d.). (D) Vascular surface was statistically significantly decreased in *tnnt2a* MO (uninjected control, *P*<0.0001; control MO, *P*=0.0007; Kruskal–Wallis test; data are mean±s.d.). (E) Vascular density was not statistically significantly changed in *tnnt2a* MO (uninjected control, *P*=0.6514; control MO, *P*=0.9082; one-way ANOVA; data are mean±s.d.). (F) Branching points were statistically significantly decreased in *tnnt2a* MO (uninjected control, *P*=0.0019; control MO, *P*=0.0092; Kruskal–Wallis test; data are mean±s.d.). (G) Vascular network length was statistically significantly decreased in *tnnt2a* MO (uninjected control, *P*=0.0033; control MO, *P*=0.0209; Kruskal–Wallis test; data are mean±s.d.). (H) Average vessel radius was statistically significantly decreased in *tnnt2a* MO (uninjected control, *P*=0.0050; control MO, *P*=0.0067; Kruskal–Wallis test; data are mean±s.d.). (I) Vascular complexity was statistically significantly decreased in *tnnt2a* MO (uninjected control, *P*=0.0050; control MO, *P*=0.0067; Kruskal–Wallis test; data are mean±s.d.).

**Fig. 4.**
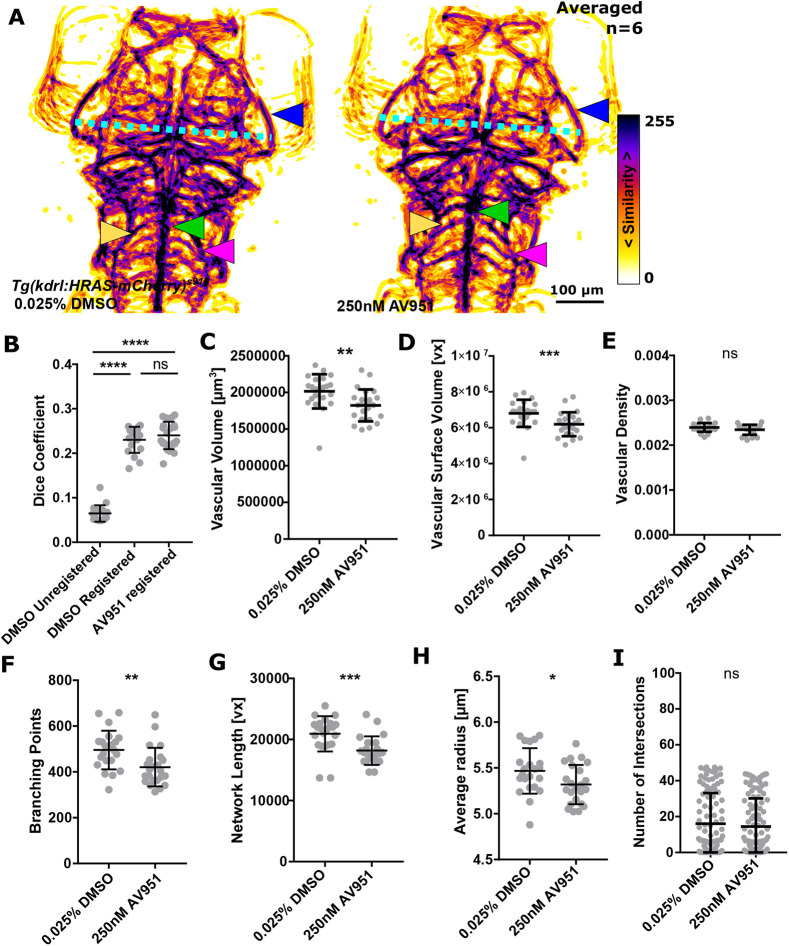
**Short-term inhibition of VEGF reduces vascular topology parameters.** (A) MIPs of averaged data of control and AV951-treated samples following segmentation and registration showed no visual phenotype [PMBC pattern (blue arrowhead), head size (PMBC′ to PMBC″ distance; cyan dotted line), BA (green arrowhead), CtAs (yellow arrowhead) and PHBC (magenta arrowhead)]. (B) No statistically significant difference was found when comparing registered controls with VEGF inhibitor-treated samples (*P*>0.9999; control=18, AV951=23; Kruskal–Wallis test; data are mean±s.d.). (C) Vascular volume was statistically significantly decreased in AV951-treated samples (*P*=0.0014; control=22, AV951=23; two-tailed Mann–Whitney *U*-test; data are mean±s.d.). (D) Vascular surface was statistically significantly decreased in AV951-treated samples (*P*=0.0010; two-tailed Mann–Whitney *U*-test; data are mean±s.d.). (E) Vascular density was not statistically significantly changed in AV951-treated samples (*P*=0.1048; unpaired two-tailed Student's *t*-test; data are mean±s.d.). (F) Branching points were statistically significantly decreased in AV951-treated samples (*P*=0.0016; two-tailed Mann–Whitney *U*-test; data are mean±s.d.). (G) Vascular network length was statistically significantly changed in AV951-treated samples (*P*=0.0004; two-tailed Mann–Whitney *U*-test; data are mean±s.d.). (H) Average vessel radius was statistically significantly reduced in AV951-treated samples (*P*=0.0371; unpaired two-tailed Student's *t*-test; data are mean±s.d.). (I) Vascular complexity was not statistically significantly changed in AV951-treated samples (*P*=0.4949; two-tailed Mann–Whitney *U*-test; data are mean±s.d.).

After showing ZVQ is able to display inter-group differences in vascular anatomy using PAMs and to detect differences in quantitative vascular parameters between different developmental stages, we next examined whether ZVQ can be used to display and quantify biologically relevant differences in embryos of the same developmental stage subjected to experimental perturbation. We first quantified the effect of absent blood flow induced by *tnnt2a* morpholino antisense knockdown. Fig. 3A shows PAMs generated by co-registering uninjected control (*n*=15), control morphant (*n*=18) and *tnnt2a* morphant embryos (*n*=10). Visual analysis shows similar PAMs, achieved by 3D image registration and represented as MIPs, between untreated and control morphants. However, the *tnnt2a* morphant PAM shows substantial anatomic alteration. Although there is high inter-embryo similarity in midbrain vessel patterning and PMBC position in the controls (both injected and uninjected), this is reduced in *tnnt2a* morphants (blue arrowhead), along with reduced head size (PMBC-to-PMBC distance; Fig. 3A, cyan dotted line). In contrast, the stereotypic patterning of the basilar artery (BA) (green arrowhead) and PHBC (magenta arrowhead) are conserved in the absence of flow. Automated quantification showed that Dice coefficient (Fig. 3B), vascular volume (Fig. 3C), vascular surface volume (Fig. 3D), branching points (Fig. 3F), network length (Fig. 3G), average vessel radius (Fig. 3H) and vascular complexity were statistically significantly decreased in *tnnt2a* morphants (Fig. 3I). The only parameter not statistically significantly altered was vascular density (Fig. 3E). This confirms that ZVQ detects biologically relevant effect sizes induced by a previously published (but not quantified) physiological manipulation. We next examined the ability of ZVQ to detect the effect of pharmacological treatments. To test this, we performed chemical inhibition of VEGF signalling, which is known to inhibit vascular growth (Bower et al., 2017; Ellis and Hicklin, 2008; Gerhardt et al., 2003). To induce a less pronounced effect on vascular patterning than in the *tnnt2a* morphants, we used very brief (2 h) VEGF-receptor (VEGFR) inhibition with 250 nM AV951 at 4 dpf. Visual assessment of the PAMs (six/group) showed no obvious difference in vascular patterning or topology between control and AV951-treated groups (Fig. 4A), suggesting that visual assessment would not detect patterning differences. However, after quantifying vascular parameters with ZVQ, vascular volume, surface volume, branch points, network length and average radius were all statistically significantly reduced (Fig. 4; Table S1). Together, this showed that quantification by ZVQ is able to detect inter-group differences that would be challenging to detect visually.

### Quantification of a range of physiological, biological and pharmacological manipulation on vascular development

**Fig. 5.**
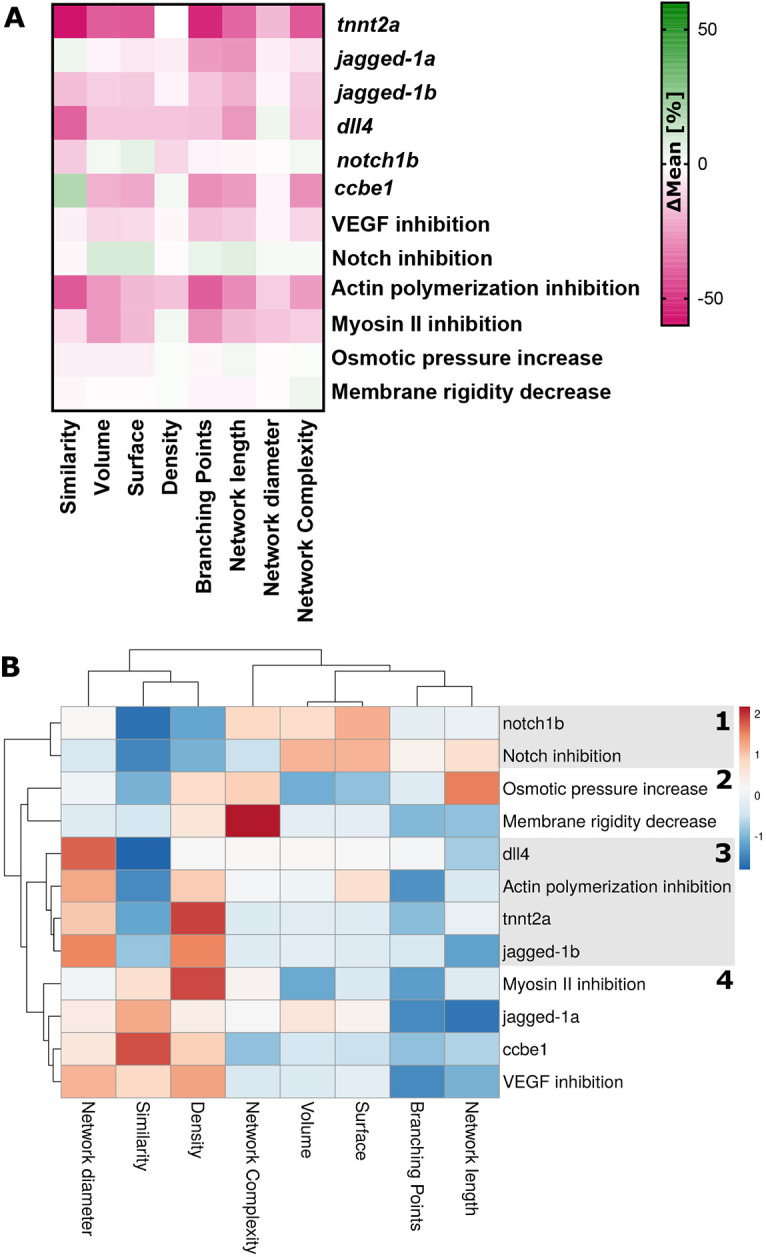
**Quantification of vascular parameters and cluster analysis.** (A) Percentage differences in mean values were quantified for the measured parameters (control MO to MO, and untreated to treated). (B) Cluster analysis identified four main clusters, including (1) inhibitors of anti-angiogenic factors (*notch1b* MO and Notch inhibition), (2) factors inducing cellular changes (osmotic pressure and membrane rigidity changes), (3) factors with severe angiogenic defects (*dll4* MO, actin polymerisation inhibition, *tnnt2a* MO and *jagged1b* MO) and (4) inhibitors of angiogenic factors (myosin II inhibition, *jagged1a* MO, *ccbe1* MO and VEGF inhibition).

We next quantified the effect of other pharmacological manipulations known to influence vascular development (Fig. 5A; Table S1). We examined the effect of Notch signalling inhibition, which was shown to result in hyper-vascularisation (Bray, 2006; Watson et al., 2013). Visual assessment of PAMs (*n*=6) showed no overt phenotype (Fig. S5), but quantification showed an increase of almost all measured vascular parameters, but only a statistically significant increase in vascular volume and surface volume (Fig. S5).

We then examined the impact of inhibiting F-actin polymerisation and myosin II cytoskeletal components, which are pivotal for EC stability and the vascular smooth muscle cell (vSMC) contractile machinery (Sacharidou et al., 2012). Visual assessment of PAMs (*n*=6) suggested, for both treatments, an overall preservation of vessel patterning but with regional alteration in the intra-neural midbrain region. Vessel diameters in the PAMs were also observably reduced in comparison with controls. Indeed, when quantifying the vascular architecture upon inhibition of F-actin polymerisation (latrunculin B; Fig. S6) and myosin II (blebbistatin; Fig. S7), a statistically significant reduction of almost all parameters was found.

Next, we studied the impact of increased osmotic pressure using glucose, which is thought to result in cellular swelling, potentially impacting angiogenesis (Scallan et al., 2010; Severinghaus, 1995), with a previous study in zebrafish showing that only exposure for over 96 h impacted vascular topology in tectal vessels (Chhabria et al., 2018). Visual assessment of PAMs (*n*=6) showed no overt phenotype. Quantifications confirmed that 24 h glucose exposure did not alter cerebrovascular topology significantly (Fig. S8; 1.31%).

Last, we examined the impact of decreased membrane rigidity (DMSO) on vascular topology as it results in increased membrane permeability (de Ménorval et al., 2012) and in transient osmotic absorption in vessels (Glass et al., 2006) and increases membrane permeability (de Ménorval et al., 2012), which could potentially change at least vessel radius. Again, no discernible phenotype was observed in the PAMs and quantification showed that 24 h exposure did not statistically significantly change vascular topology (Fig. S9).

We next examined the effect of previously published morpholinos on the vascular architecture, to further assess the performance of ZVQ and provide quantitative insights into the effects of these morpholinos on cerebral angiogenesis.

Jagged1 has previously been shown to be a proangiogenic regulator in mice, but so far no study has examined the distinctive roles of the zebrafish orthologues *jagged1a* or *jagged1b* in zebrafish angiogenesis. Visual assessment of PAMs of both *jagged1a* and *jagged1b* morphants suggested patterning alterations in intra-neural midbrain vessels. Quantification by ZVQ showed morpholino knockdown of *jagged1a* decreased network length and branching points (Fig. S10), while knockdown of *jagged1b* reduced vascular volume, surface volume and network length (Fig. S11). We next examined the impact of *dll4* knockdown, which was previously shown to result in hyper-vascularisation in the zebrafish trunk (Leslie et al., 2007) and mouse retina (Lobov et al., 2007). PAMs indicated that *dll4* morphants had a smaller head size, with patterning changes in the midbrain, but not hindbrain. Our quantification showed a decrease of all vascular parameters, except radius and complexity (Fig. S12).

Loss of *notch1b* has previously been shown not to significantly impact vascular topology in the zebrafish trunk (Quillien et al., 2014). Similarly, PAMs displayed no observable difference, and quantification showed no statistically significant difference in the cerebrovascular architecture upon *notch1b* knockdown (Fig. S13).

Previous work showed that *ccbe1* is required for lymphangiogenesis and venous differentiation (Hogan et al., 2009), and loss of *ccbe1* impacts the murine coronary artery (Bonet et al., 2018). PAMs suggested alterations of the midbrain vasculature, with quantification of vascular topology after *ccbe1* knockdown showing a significant reduction of volume, surface volume, branching points and network length (Fig. S14).

To further study the impact of the examined morpholinos and chemicals on vascular topology, we quantified percentage differences in mean values of all of the measured quantitative parameters (Fig. 5A; Table S1; control morphant to morphant, and untreated to treated; see Movies 1-16), whereas percentage differences were used to allow comparison between groups.

Cluster analysis revealed five main clusters, which can be considered as: (1) inhibitors of anti-angiogenic factors (*notch1b* morpholino and Notch inhibition), (2) factors inducing cellular changes (osmotic pressure and membrane rigidity changes), (3) factors with severe angiogenic defects (*dll4* morpholino, actin polymerisation inhibition, *tnnt2a* morpholino and *jagged1b* morpholino), and (4) inhibitors of angiogenic factors (myosin II inhibition, *jagged1a* morpholino, *ccbe1* morpholino and VEGF inhibition) (Fig. 5B). Our data showed that ZVQ provides informative population average maps and extracts meaningful parameters to quantify and describe vascular topology.

### Comparing regional similarity and left-right symmetry

Next, we examined regional vascular topology to examine the ability of ZVQ to quantify vascular anatomy in smaller, specific regions of interest. Our initial manual measurements of local vascular growth had shown that growth was comparable between fish (Fig. S2), thus embryos could be compared with each other on a scale.

**Fig. 6.**
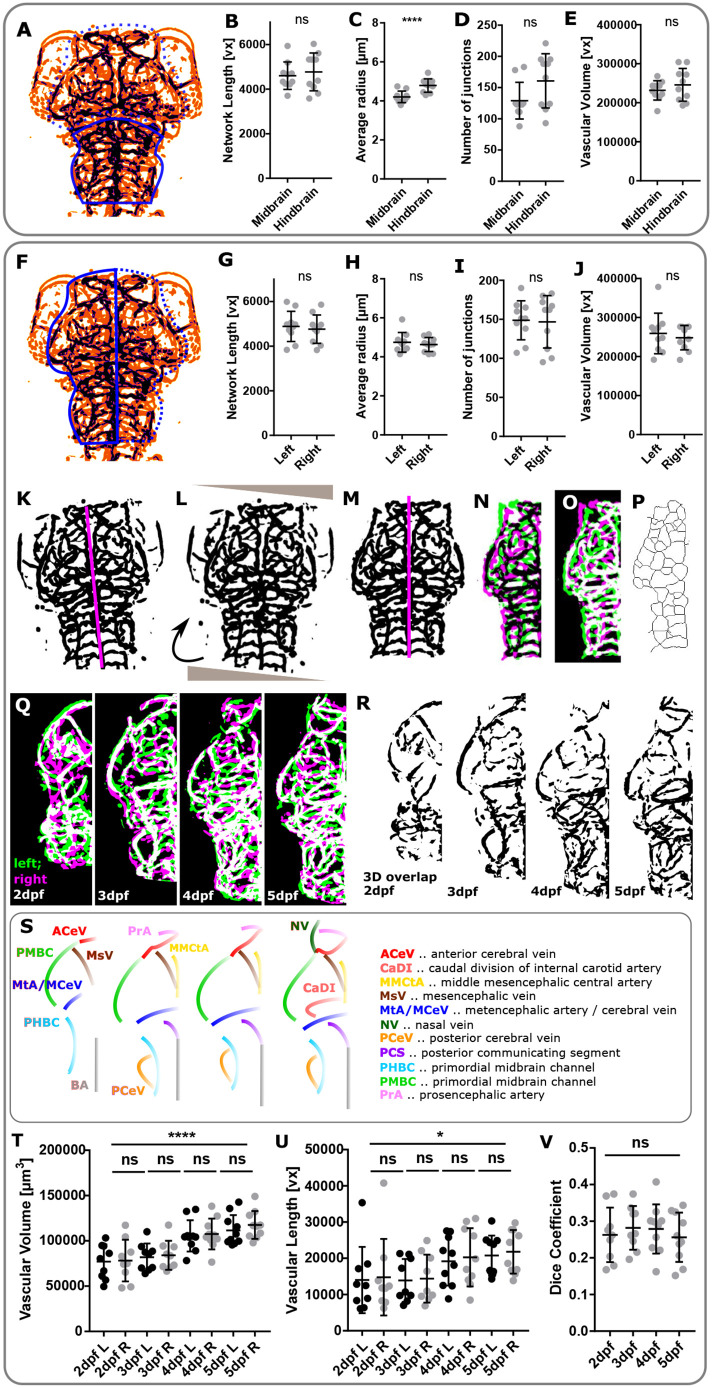
**Comparing regional similarity and left-right symmetry.** (A) ROI selection allows comparison of vascular parameters in the midbrain (dotted line) and hindbrain (solid line). (B) Comparing the midbrain to the hindbrain, network length was not statistically significantly different (*P*=0.6168, *n*=10, unpaired two-tailed Student's *t*-test; data are mean±s.d.). (C) The average vessel radius was statistically significantly higher in the hindbrain (*P*=0.0008, *n*=10, unpaired two-tailed Student's *t*-test; data are mean±s.d.). (D) The number of junctions was not statistically significantly different (*P*=0.0717, *n*=10, unpaired two-tailed Student's *t*-test; data are mean±s.d.). (E) Vascular volume was not statistically significantly different (*P*=0.3698, *n*=10, unpaired two-tailed Student's *t*-test; data are mean±s.d.). (F) ROI selection allows comparison of vascular parameters in the right (broken line) and left (solid line) brain hemisphere. (G) Comparing the left and right vasculature, network length was not statistically significantly different (*P*=0.6830, *n*=10, unpaired two-tailed Student's *t*-test; data are mean±s.d.). (H) The average vessel radius was not statistically significantly different (*P*=0.5665, *n*=10, unpaired two-tailed Student's *t*-test; data are mean±s.d.). (I) The number of junctions was not statistically significantly different (*P*=0.8816, *n*=10, unpaired two-tailed Student's *t*-test; data are mean±s.d.). (J) Vascular volume was not statistically significantly different (*P*=0.5730, *n*=10, unpaired two-tailed Student's *t*-test; data are mean±s.d.). (K) Manual selection of left-right body axis (magenta) in segmented template fish used for image rotation and left-right separation in registered segmented images. (L) Image rotation was performed to align sample anterior-posterior axis with image *x*-axis. (M) Right vascular volume was mirrored and vascular volume quantified for the left and right vasculature. (N,O) Similarity measures were extracted to compare left and mirrored right vasculature. (P) Left and right vascular network lengths were quantified after skeletonisation to extract vascular centrelines (representative images). (Q) MIPs of left (green) and right (magenta) vasculature, showing regions of similarity (white). (R) Representative micrographs of regions of left-right vascular overlap from one example fish. (S) Schematics showing left-right symmetric vessels from 2 to 5 dpf. (T) Cranial vascular volume was found to statistically significantly increase from 2 to 5 dpf (****P<0.0001), while no statistically significant difference was found between the vascular volume of the left (L) and right (R) brain hemispheres (L-R; 2 dpf, *P*<0.9999, *n*=9 embryos; 3 dpf, *P*>0.9999, *n*=9 embryos; 4 dpf, *P*>0.9999, *n*=9 embryos; 5 dpf, *P*=0.9946, *n*=10 embryos; two experimental repeats; one-way ANOVA; data are mean±s.d.). (U) Cranial vascular network length was found to statistically significantly increase from 2 to 5 dpf (*P=0.0194), while no statistically significant L-R difference was found (L-R; 2 dpf, *P*<0.9999; 3 dpf, *P*>0.9999; 4 dpf, *P*>0.9999; 5 dpf, *P*>0.9999; *n* values as above; Kruskal–Wallis test; data are mean±s.d.). (V) The Dice co-efficient was not statistically significantly changed between the left and right vasculature from 2 to 5 dpf (*P*=0.8058; one-way ANOVA; 2 dpf, *n*=9 embryos; 3 dpf, *n*=9 embryos; 4 dpf, *n*=10 embryos; 5 dpf, *n*=10 embryos; data are from two experimental repeats; data are mean±s.d.).

We quantified vascular parameters in the midbrain and hindbrain regions at 3 dpf (Fig. 6A). As this has never been carried out before, it was unclear whether vessel radius, network length, branching points or volume would be similar in these regions. We found vascular network length (*P*=0.6168; Fig. 6B), branch points (*P*=0.0717; Fig. 6D) and vascular volume (*P*=0.0717; Fig. 6E) to be not significantly different, while the average vessel radius was significantly higher in the hindbrain (*P*=0.0008; Fig. 6C). To understand the reason for this, comparing vessel radii further revealed that the BA was the main contributing factor for the increased average vessel radius in the hindbrain (Figs S15, S17). We used the same approach to quantify the vasculature of the left and right brain at 3 dpf (Fig. 6F). We found no statistically significant difference in network length (*P*=0.6830; Fig. 6G), average vessel radius (*P*=0.5665; Fig. 6H), number of junctions (*P*=0.8816; Fig. 6I) or vascular volume (*P*=0.5730; Fig. 6J). To examine left-right vascular symmetry in more detail and over time, we then developed an image analysis workflow to assess topological similarity of the left and right vasculature automatically (Fig. 6K-P). This was achieved by mirroring the vasculature from the right brain hemisphere to the left by horizontal transformation (Movie 17) and extracting vessel overlap by voxel majority decisions (Fig. 6Q,R). This suggested that peripheral/perineural vessels show a strong degree of left-right symmetry from 2 to 5 dpf (Fig. 6S). Quantification of vascular volume (Fig. 6T) and network length (Fig. 6U), showed no statistically significant difference from 2 to 5 dpf, although the Dice coefficient between the left and right vasculature was low (Fig. 6V). Thus, our quantification approach allows for novel regional insights of vascular topology, showing that the average vessel radius is higher in hindbrain vessels than midbrain vessels, but that the vasculature is highly symmetric from 2 to 5 dpf.

We also examined whether regional differences between sample groups could be extracted by image calculation of overlap and difference (Fig. S16), finding that regions of similarity and overlap could indeed be calculated. This allows researchers to identify regions of interest, which could be used to examine these extracted regions in more detail via specific ROI selection.

Last, we examined whether information could be extracted from individual vessels. Theoretically, various approaches for this exist. For example, (1) vessel start and endpoints could be extracted, and cubic ROIs drawn automatically (Fig. S17A); however, this is inaccurate because of vessel proximity and curvature. (2) Individual vessels could be extracted using a brain atlas (Fig. S17B), as previously shown for mice (Todorov et al., 2020); however, zebrafish brain atlases are only available for 6 dpf and healthy controls; an alternative could be to use a vessel-based atlas, which is likely to work at least for major vessels, but not for smaller non-stereotypic vessels. (3) Individual vessels could be annotated manually and annotations transferred to other vessels (Fig. S17C). We examined this last option using simple neurite tracer (SNT) (Longair et al., 2011), finding it, in theory, applicable, but highly laborious (annotation for one fish taking ∼8 h; Fig. S17D-I).Together, our approach allows for regional studies of the cerebral vasculature, but future work is needed to allow extraction and annotation of individual vessels.

### Dissemination of the presented analysis approach

As our aim was to provide a useful and easy-to-use image analysis approach for the community without prior computational knowledge, the following steps were undertaken to allow uptake by the community:

1. ZVQ was implemented as one Macro in the open-source software Fiji, which is free to use and is cross-platform applicable. Additionally, the workflow was designed in a modular fashion, allowing users to run one or multiple analysis steps at a time.
2. A graphical user interface (GUI) was designed to allow users to select the required steps using drop-down boxes without the need to code (Fig. S18).
3. The code is provided on Github, accompanied by an in-depth step-by-step workflow documentation (explaining individual steps, input and output); importantly, the code will be maintained by the lead author, but is free to be modified and adapted by other researchers, depending on their data and analysis needs.
4. Example data are provided to allow users to examine the workflow first, before applying it to their own data.
5. We have included reviewer and user feedback to improve the presented workflow for use.

## DISCUSSION

In this work, we present ZVQ – the first biomedical image analysis workflow that allows 3D zebrafish cerebrovascular quantification, including: (1) inter-sample registration and generation of population average maps; (2) quantification of parameters (vascular volume, surface volume, density, network length, branching points, radii and network complexity); and (3) quantification of regional vascular parameters comparing midbrain with hindbrain, left with right and regional vascular topology, using the freely available image analysis software framework Fiji.

Our pipeline builds on a validated segmentation approach, applicable for all examined ages and data. Should users wish to further improve segmentation outcomes, this could be achieved by raw data quality improvement, such as (1) prevention of pigmentation using chemicals or zebrafish non-pigmented lines, or (2) multi-view image acquisition and fusion.

When examining registration methods, we found manual landmark-based as well as automatic rigid registration were applicable to our data, and suggest that automatic registration is preferable based on the decreased CoV and the significant reduction in user interaction. However, as for all automatic approaches, registration outcomes should be visually and, where possible, quantitatively assessed. Future work is needed to examine whether registration outcomes could be improved using iterative registration or bespoke solutions optimised for sparse vascular datasets.

Registration resulted in low Dice scores for both registration methods. However, unlike in the medical field, no reference values were available to examine our registration outcomes, as we here present zebrafish vascular inter-sample registration for the first time. To address this, we validated our segmentation approach with experimental data from the same sample after displacement and examined other similarity measurement scores, including Jaccard index, total overlap, mutual information, sum of squared differences, mean square error and structural similarity. As all of these showed similar results, we opted to display the Dice coefficient, owing to its implementation in the free open-source software Fiji and computational efficiency. We anticipate that data sparsity of the segmented vasculature is one contributing factor for these low Dice scores, as small shifts between vessels caused either by inter-sample variation or residual registration errors will affect the overlap more drastically than would be the case for more continuous structures such as brain tissue. Future work is needed to examine Dice coefficients on vessel-segment levels, to study voxel-wise comparisons, to explore alternative similarity metrics more suited to a sparse vascular network and to develop registration methods that utilise higher order transformations.

Our registration approach allows examination of regional similarity, as well as facilitating the development of a vascular atlas. Future work could use double transgenics to allow other information, such as expression patterns, to be co-registered to the vascular atlas, as has been performed for a zebrafish brain atlas (Randlett et al., 2015; Marquart et al., 2017).

We showed ZVQ is able to extract data from different ages (2-5 dpf examined). We anticipate that the change from 2-3dpf in vascular density is likely to be due to the higher head curvature at 2 dpf, rather than to a smaller volume (Fig. 2E) or head size (Fig. S3). We suggest that the transient dip observed in vascular surface volume at 4 dpf could potentially be due to: (1) the amount of pruning starting at 3 dpf resulting in decreased surface area, and (2) the fact that myogenic responses and vascular regulation start at 4 dpf and might alter surface transiently. However, as there is no ‘gold-standard’ to compare our data against, future work is needed to examine this further.

Our studies examining vascular anatomy in groups of animals with and without experimental perturbation (physiological, chemical or genetic) showed ZVQ is able to detect statistically significant differences with relatively low group sizes and despite relatively mild patterning defects in many cases. For example, we show the first quantitative assessments of decreased and increased cerebral vascular patterning in response to VEGF and Notch inhibition, respectively. This is in agreement with the literature that VEGF is pro-angiogenic, while Notch is angiogenesis limiting (Lobov et al., 2007; Phng and Gerhardt, 2009; Hogan and Schulte-Merker, 2017). As expected, our data show that inhibition of F-actin polymerisation or myosin II both result in a reduction of vascular parameters, and we anticipate this is mainly caused by a mechanical collapse of the cytoskeleton and the vascular lumen.

The unchanged vascular topology upon glucose treatment is in agreement with previous work, which showed 24 h exposure has no significant effect on vascular topology (Chhabria et al., 2018). Additionally, the unchanged vascular topology upon DMSO treatment suggests that DMSO indeed only leads to transient vascular changes, as previously suggested (Glass et al., 2006).

These findings clearly demonstrate that quantification of 3D vascular parameters enables meaningful insights into the effects of a range of manipulations on vascular anatomy, and is sensitive enough to extract true biological differences, resulting in a decrease or increase of the vasculature. When examining morpholino data, we show for the first time that loss of *jagged1a* and *jagged1b* results in mild cerebrovascular defects. As *jagged1a* and *jagged1b* are paralogues found in zebrafish due to partial genome duplication (Meyer and Schartl, 1999), we anticipate that their simultaneous loss might result in a stronger effect.

Finding that *dll4* knockdown results in a reduction of the cerebral vasculature is in contrast to previous work in the zebrafish trunk (Leslie et al., 2007). This suggests that *dll4* might have cerebrovascular-specific roles that have been previously overlooked and require future investigation.

Our finding that loss of *notch1b* does not significantly impact vascular topology suggests that *notch1b* plays a conserved role in the zebrafish head and trunk vasculature (Quillien et al., 2014).

Having found that loss of *ccbe1* results in a decrease of vascular volume, surface volume, branching points and network length. We postulate that *ccbe1* has a conserved function in different vascular beds, as previous work has shown that *ccbe1* is required for zebrafish lymphangiogenesis and venous differentiation (Hogan et al., 2009), as well as murine coronary artery development (Bonet et al., 2018).

Moreover, we developed an automated approach to study the vasculature in defined regions, illustrating the usefulness of the approach in the midbrain and hindbrain, as well as for comparison of the left and right vasculature. However, the implementation of our analysis approach is not limited to this, allowing the user to select a variety of regions of interest.

Future studies are needed to examine vascular symmetry between the left and right vasculature, as well as to examine stochasticity rules of vascular patterning in more depth, to assess local differences and how these might relate to structural and/or functional brain asymmetry, which is established at 4 dpf (Dreosti et al., 2014). As the zebrafish brain vasculature is an enclosed system, which is in contrast to the human cerebrovascular tree, localised effects, such as by a stroke, are likely to translate to global outcomes. We anticipate that our quantification approach will be feasible to allow examination of left-right symmetry in older zebrafish, which may be more likely to show vascular asymmetry. Our quantification was performed at levels of the entire brain or in subregions, and was sufficient to detect significant differences during development and in response to manipulations. Future work might examine vascular parameters in older zebrafish, other transgenic lines and vessel-specific quantification, which will require an in-depth annotation.

We presented a proof-of-principle example on how to examine individual vessels, but future work is needed to establish brain and vascular atlases for zebrafish at varying ages and of varying vascular phenotypes to allow automatic annotation and extraction on vessels. Currently, atlases for the zebrafish brain exist for only healthy animals at 6 dpf (Randlett et al., 2015; Kunst et al., 2019) and adults (Kenney et al., 2021; Ullmann et al., 2010). This means any analysis in other ages or treatment groups would require creation of new atlases to allow for brain/morphological differences with dedicated experiments and analysis. Alternatively, new atlases could possibly be developed using available information and modelling age-specific changes. However, this would require extensive *a priori* information and validation. To allow application of the analysis approach by other researchers, we have implemented a GUI, and we provide code, example data and step-by-step workflow documentation.

### Conclusions

We describe ZVQ – the first comprehensive workflow for assessing zebrafish 3D cerebrovascular similarity, topology and regional variability. Our data show that robust image analysis is needed to allow objective and quantitative insights. Our results emphasise that the cerebral vasculature is highly complex and that many mechanisms regulate its formation and patterning.

## MATERIALS AND METHODS

### Zebrafish strains, handling and husbandry

Experiments performed at the University of Sheffield conformed to UK Home Office regulations and were performed under Home Office Project Licence 70/8588 held by T.J.A.C. Maintenance of adult zebrafish in the fish facilities was conducted according to previously described husbandry standard protocols at 28°C, with a 14:10 h (h) light:dark cycle (Westerfield, 1993; Aleström et al., 2020). Embryos, obtained from controlled pair- or group-mating, were incubated in E3 buffer (5 mM NaCl, 0.17 mM KCl, 0.33 mM CaCl_2_ and 0.33 mM MgSO_4_) with or without Methylene Blue. All experiments were conducted with the transgenic reporter line *Tg(kdrl:HRAS-mCherry)^s916^* (Chi et al., 2008).

### Data for biological application

Data described in this section were obtained from Kugler et al. (2019b). Briefly, the impact of nine chemical components and six morpholinos (Genetools) on the vascular architecture was examined. Morpholino injections were performed at the one-cell stage using Phenol Red as an injection marker.

#### Chemical components

The conditions studied, the compounds used and the *n* values were as follows. VEGF inhibition was achieved using 250 nM AV951 for 2 h (Nakamura et al., 2006) at 4 dpf (control, *n*=22; treated, *n*=23; three experimental repeats). Notch inhibition was achieved using 50 µm DAPT for 12 h (Geling et al., 2002) at 4 dpf (control, *n*=24; treated, *n*=24; three experimental repeats). Myosin II inhibition was achieved using 25 µM blebbistatin for 1 h (Kovács et al., 2004) at 4 dpf (control, *n*=21; treated, *n*=23; 3 experimental repeats). F-actin polymerisation inhibition was achieved with 100 nM Latrunculin B for 1 h (Morton et al., 2000) at 4 dpf (control, *n*=13; treated, *n*=12; two experimental repeats). Osmotic pressure was studied using 40 nM glucose for 24 h at 4 dpf (control, *n*=18; treated, *n*=18; two experimental repeats). Membrane rigidity was assessed using 0.25% DMSO for 24 h at 4 dpf (control, *n*=24; treated, *n*=22; three experimental repeats).

#### Morpholinos

The following morpholinos were used: cardiac troponin T2a (*tnnt2a*; 1.58 ng; Genetools; 5′-CATGTTTGCTCTGATCTGACACGCA-3′) (Sehnert et al., 2002); *jagged1a* (*jag1a*; 0.1 ng; Genetools; 5′-GTCTGTCTGTGTGTCTGTCGCTGTG-3′) (Geudens et al., 2010); *jagged1b* (*jag1b*; 0.8 ng; Genetools; 5′-CTGAACTCCGTCGCAGAATCATGCC-3′) (Geudens et al., 2010); *delta-like ligand 4* (*dll4*; 3 ng; Genetools; 5′-GAGAAAGGTGAGCCAAGCTGCCATG-3′) (Geudens et al., 2010); *notch1b* (0.25 ng; Genetools; 5′-GTTCCTCCGGTTACCTGGCATACAG-3′) (Quillien et al., 2014); and calcium-binding EGF-like domain 1 (*ccbe1*) (5 ng; Genetools; 5′-CGGGTAGATCATTTCAGACACTCTG-3′) (Guen et al., 2014). Control MO injection was performed according to the same protocol, with final concentrations as above (Genetools; 5′-CCTCTTACCTCAGTTATTTATA-3′).

#### Morpholino sample data

Experimental repeats and *n* values for each morpholino were as follows. Cardiac troponin T2a: 3 dpf uninjected control, *n*=17; control MO, *n*=18; MO, *n*=15; two experimental repeats. *jag1a*: 3 dpf uninjected control, *n*=11; control MO, *n*=12; MO, *n*=10; two experimental repeats. *jag1b*: 3 dpf uninjected control, *n*=21; control MO, *n*=21; MO, *n*=21; three experimental repeats. *dll4*: 3 dpf uninjected control, *n*=23; control MO, *n*=23; MO, *n*=23; three experimental repeats. *notch1b*: 3 dpf uninjected control, *n*=21; control MO, *n*=21; MO, *n*=21; three experimental repeats. *ccbe1*: 3 dpf uninjected control, *n*=22; control MO, *n*=22; MO, *n*=22; three experimental repeats.

### Image acquisition

Datasets were obtained using a Zeiss Z.1 light sheet microscope with a Plan-Apochromat 20×/1.0 Corr nd=1.38 objective and a scientific complementary metal-oxide semiconductor (sCMOS) detection unit. Data were acquired with activated pivot scan, dual-sided illumination and online fusion. Properties of acquired data are as follows: 0.7× zoom, 16 bit image depth, 1920×1920 pixel (∼0.33×0.33 µm) image size and minimum *z*-stack interval (∼0.5 µm), 561 nm laser, LP560 and LP585. Zebrafish embryos were embedded in 2% LMP-agarose containing 200 mg/l tricaine (MS-222, Sigma). The image acquisition chamber was filled with E3 plus tricaine (200 mg/ml) and maintained at 28°C.

### Image analysis

Images were analysed using open-source software Fiji (Schindelin et al., 2012). Data analysis was performed without blinding.

#### Pre-processing and segmentation

Data were pre-processed by performing a .czi to .tiff conversion and by generation of maximum intensity projections (MIPs) using the Bio-Formats plug-in. Intra-stack motion correction was performed based on scale invariant feature transformation (SIFT) (Lowe, 1999, 2004) using the Fiji plug-in ‘Linear Stack Alignment with SIFT’ with parameters as described previously (Kugler et al., 2018, 2019a).

Vascular enhancement was performed assuming local vessel tubularity using the Fiji Sato enhancement filter ‘Analyse>Tubeness’ (Sato et al., 1998) with an optimised parameter set as described previously (Kugler et al., 2018, 2019a,b). Enhanced images were segmented using Otsu thresholding (Otsu, 1979) as described in previously (Kugler et al., 2018, 2019a).

#### Manual measurements

The following measurements were performed manually using the Fiji line region of interest (ROI) tool and ‘Analyse>Measure’. Growth expansion of the primordial midbrain channel (PMBC) was measured at the inner vascular edge at the position behind the eyes. Distance of the middle metencephalic central artery (MMCtA) was measured at the most anterior point. The MMCtA angle was measured in the right MMCtA from its posterior to its anterior end. Posterior metencephalic central artery (PMCtA) angle was measured from its origin in the centre of the embryo to its right lateral end.

#### Inter-sample registration

Visual comparison of samples was used to identify and extract 11 anatomical landmarks that were found in all samples and distributed along the anterior-posterior, left-right and dorso-ventral body axis. These 11 anatomical landmarks (Fig. 2) were then used for manual landmark-based rigid registration using the Fiji plug-in ‘Name Landmarks and Register’. The automatic rigid inter-sample registration method, by Johannes Schindelin and Mark Longair, based on the virtual insect brain protocol (VIB) (Schmid, 2010; Jenett et al., 2006), was used with the following parameters: no initial transformation, five best matching orientations for further optimisation, image downsampling two to six times, no ROI reduction (i.e. bounding box selection) for optimisation, using Euclidean measure of difference and showing transformed image.

Target embryos, used as a template for registration, were selected based on (1) sample body-axis orientation along the image axis (anterior-posterior along image *y*-axis, coronal plane along image *z*-axis and *x*-axis); (2) all major vessels visualised in image; and (3) no obvious abnormalities (i.e. vascular patterning visually normal, no haemorrhaging and no gross defects).

To allow comparison between sample groups, all embryos were registered to a template from the control group, while ROIs for quantification were drawn for one sample/template per group. To validate registration, we constructed a new dataset by repetitive image acquisition of the same sample after manual displacement. This allowed quantification of registration outcomes following registration to the initial acquisition. To further examine 3D topological similarity between samples, six embryos were registered to a target and averages extracted.

#### Similarity assessment

Vascular intra-sample left-right and inter-sample similarity (S_n_) between target (T) and moving image (M) were quantified using the Fiji plug-in MorphoLibJ (Legland et al., 2016), Dice coefficient [DC_(M,T)_; Eqn 1], Jaccard index [JI_(M,T)_; Eqn 2] and total overlap [TO_(M,T)_; Eqn 3].
(1)

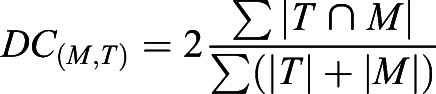

(2)

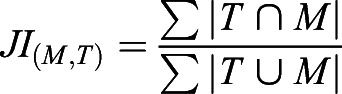

(3)

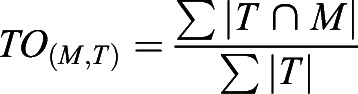
Mutual Information [MI_(M,T)_; Eqn 4], sum of squared differences [SSD_(M,T)_; Eqn 5], mean square error [MSE_(M,T)_; Eqn 6] and structural similarity [SSIM_(M,T)_; Eqn 7] (Taha and Hanbury, 2015; Wang et al., 2004) were computed using MATLAB.
(4)

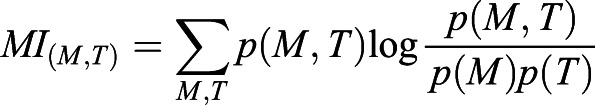

(5)

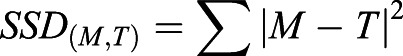

(6)

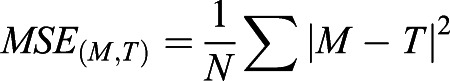

(7)

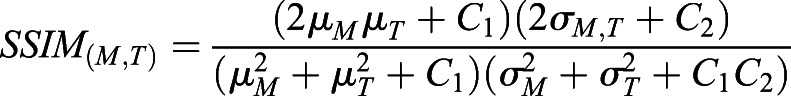


µ is the local mean and σ is the standard deviation.

### Quantification

Vascular volume (V_n_; Eqn 8) quantification was performed in a cranial ROI as described previously (Kugler et al., 2018, 2019a), by multiplying histogram black voxel count (V_black_) following segmentation by the respective voxel volume (V_x,y,z_).
(8)

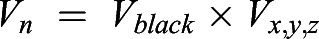
Vascular surface voxels (A_n_) were quantified in the same cranial ROI after edge detection using Canny Edge detection, multiplying voxel number of edges by voxel size (Canny, 1986).

Vascular density (D_n_; Eqn 9) was quantified as vascular volume (V_n_) as a proportion of total cranial ROI volume (V_total_).
(9)

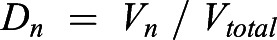
Next, data were downsampled (1920 pixels to 512 pixels using bilinear interpolation) to decrease processing time in the following steps:

Euclidean distance maps (EDM_n_) of vascular voxel (v) distance to the nearest background voxel (b) were produced from binary segmented images using the Fiji plug-in ‘Distance Map 3D’, which calculates distance in three-dimensional Euclidean space [Eqn 10; process>binary>distance map in 3D; implemented by Jens Bache-Wiig and Christian Henden (Borgefors, 1996)].
(10)

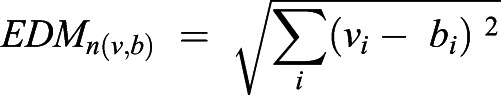
Centre-line extraction was performed in segmented images using the Fiji ‘Skeletonise 2D/3D’ plug-in (by Ignacio Arganda-Carreras; Arganda-Carreras et al., 2010), based on 3D thinning (Lee et al., 1994), using a layer by layer removal. Centre-line voxels (valued as zero in images) were quantified for total network length (L_n_) analysis by quantification of vascular voxels (zero value) in the histogram.

The ‘Analyse Skeleton’ plug-in in Fiji (analyse>skeleton>analyse skeleton 2D/3D; implemented by Ignacio Arganda-Carreras (Arganda-Carreras et al., 2010) was used to identify and measure the number of branching points (analyse>skeleton>summarise skeleton). To quantify vessel radii (R_n_), EDMs were multiplied with extracted skeletons, resulting in a 1D representation of vessel radii as represented by intensity of voxels.

To analyse vascular complexity (C_n_) by assessment of branching, Sholl dentritic arbour analysis (Eqn 11) (Sholl, 1953; Bird and Cuntz, 2019) was applied to 2D skeleton MIPs using the Fiji Sholl Analysis plug-in (Ferreira et al., 2014) (developed and maintained by Tom Maddock, Mark Hiner, Curtis Rueden, Johannes Schindelin and Tiago Ferreira):
(11)

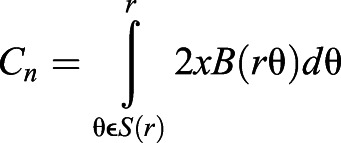
where C_n_ is vascular complexity given by Sholl intersection profiles as number of branches with the distance from the shell centre, calculated with spheres of radius S(r), B being the region of interest examined, θ being a point on S(r) and χB(r,θ) being the function for point (r,θ) in B. Default parameters were used to create a sphere, except for distance from the centre (700 voxel being the sphere size) and step size (five voxel being the shell step-size). The centre of the sphere was placed in the midbrain at the BA-PCS junctions.

#### Intra-sample symmetry

Intra-sample symmetry was assessed using the segmented vasculature to allow topological rather than intensity-based assessments. After manual selection of the central body axis [centrally along the basal aorta (BA), between L-R posterior communicating segments (PCS), between L-R MMCtA and the bifurcation point of L-R anterior cerebral vein (ACeV)], automatic image rotation was performed to align the anterior-posterior axis of samples with the image *y*-axis to allow for mirroring. Afterwards, the right vascular volume was automatically mirrored by horizontal image transformation and similarity was derived via voxel overlap.

#### Manual vessel tracing

Semi-automatic tracing and annotation of cerebral vessels was performed using the Fiji plug-in Simple Neurite Tracer (SNT) (Longair et al., 2011). To allow detection of these vessels in other fish (following segmentation, registration and skeletonisation), traces were extracted as vessel-specific ROIs (multiple ROIs to describe *x*, *y* and *z* position). These vessel-specific ROIs were then used to produce rectangular ROIs (*x*,*y*; 30×130 voxels) to allow the detection of centrelines in other fish. The ROIs were then used to detect and quantify vessel intensities (from EDM and skeleton) to quantify vessel-specific diameters.

#### Code and documentation

The code is available on Github (https://github.com/ElisabethKugler/ZFVascularQuantification) and is accompanied by in-depth step-by-step workflow documentation (*SDoc1*; questions and bug reports to be sent to E.C.K.). A doi has been assigned with Zenodo (https://doi.org/10.5281/zenodo.3978278). The workflow is designed in a modular fashion, allowing users to run individual or multiple steps at once, depending on their data and analysis needs. It was implemented in an open-source framework to allow adaption and changes by the community. Example data are available on zenodo (10.5281/zenodo.4108660; https://zenodo.org/record/4108660#.X47XU9BKizc).

#### Data representation

To visualise data, maximum intensity projections (MIPs) were generated and intensity inversion was applied as appropriate to give the clearest rendering of relevant structures.

### Statistical analysis

Normality of data was tested using D'Agostino-Pearson omnibus test. Statistical analysis of normally distributed data was performed using one-way ANOVA to compare multiple groups or Student's *t*-test to compare two groups. Non-normally distributed data were analysed with a Kruskal–Wallis test to compare multiple groups, or a Mann–Whitney test to compare two groups. Analysis was performed in GraphPad Prism Version 7. *P* values are indicated as follows: *P<0.05, **P<0.01, ***P<0.001 and ****P<0.0001. Based on the mean and s.d. of the data in the examined groups in the assays used, post-hoc power calculations have shown that these assays have at least 80% power to detect a 30% effect difference between groups when group sizes are 12/group (α=0.05). Samples were excluded from analysis if image quality did not allow for reliable quantification. Animals were allocated to treatment groups randomly without selection. Imaging and data analysis were performed unblinded to treatment allocation, often because the effect of treatment was easily deduced from the appearance of the micrograph. To overcome subjective bias, objective automated image analysis was applied where possible. Data are mean±s.d., if not otherwise stated. Cluster analysis was performed using ClustVis (Metsalu and Vilo, 2015). Settings used were as follows: clustering distance – correlation, clustering method – average, tree ordering – tightest cluster first. Clustering validation was performed using relative clustering validation (varying the number of clusters from one to three; one displayed). Image representation was performed using Inkscape (https://www.inkscape.org).

## Supplementary Material

Supplementary information

Reviewer comments

## References

[DEV199720C1] Aleström, P., D'Angelo, L., Midtlyng, P. J., Schorderet, D. F., Schulte-Merker, S., Sohm, F. and Warner, S. (2020). Zebrafish: Housing and husbandry recommendations. *Lab. Anim.* 54, 213-224. 10.1177/0023677219869037 31510859 PMC7301644

[DEV199720C2] Arganda-Carreras, I., Fernández-González, R., Muñoz-Barrutia, A. and Ortiz-De-Solorzano, C. (2010). 3D reconstruction of histological sections: Application to mammary gland tissue. *Microsc. Res. Tech.* 73, 1019-1029. 10.1002/jemt.20829 20232465

[DEV199720C3] Bird, A. D. and Cuntz, H. (2019). Dissecting sholl analysis into its functional components. *Cell Reports* 27, 3081-3096.e5. 10.1016/j.celrep.2019.04.097 31167149

[DEV199720C4] Bonet, F., Pereira, P. N. G., Bover, O., Marques, S., Inácio, J. M. and Belo, J. A. (2018). CCBE1 is required for coronary vessel development and proper coronary artery stem formation in the mouse heart. *Dev. Dyn.* 247, 1135-1145. 10.1002/dvdy.24670 30204931

[DEV199720C5] Borgefors, G. (1996). On digital distance transforms in three dimensions. *Comput. Vis. Image Underst.* 64, 368-376. 10.1006/cviu.1996.0065

[DEV199720C6] Bower, N. I., Vogrin, A. J., Le Guen, L., Chen, H., Stacker, S. A., Achen, M. G. and Hogan, B. M. (2017). Vegfd modulates both angiogenesis and lymphangiogenesis during zebrafish embryonic development. *Development* 144, 507-518. 10.1242/dev.146969 28087639

[DEV199720C7] Bray, S. J. (2006). Notch signalling: a simple pathway becomes complex. *Nat. Rev. Mol. Cell Biol.* 7, 678-689. 10.1038/nrm2009 16921404

[DEV199720C8] Canny, J. (1986). A computational Approach to Edge Detection. IEEE Transactions on Pattern Analysis and Machine Intelligence PAMI-8, 679-698. 21869365

[DEV199720C9] Chávez, M. N., Aedo, G., Fierro, F. A., Allende, M. L. and Egaña, J. T. (2016). Zebrafish as an emerging model organism to study angiogenesis in development and regeneration. *Front. Physiol.* 7, 56. 10.3389/fphys.2016.00056 27014075 PMC4781882

[DEV199720C10] Chen, Q., Jiang, L., Li, C., Hu, D., Bu, J.-w, Cai, D. and Du, J.-l. (2012). Haemodynamics-driven developmental pruning of brain vasculature in Zebrafish. *PLoS Biol.* 10, e1001374. 10.1371/journal.pbio.1001374 22904685 PMC3419171

[DEV199720C11] Chhabria, K., Plant, K., Bandmann, O., Wilkinson, R. N., Martin, C., Kugler, E., Armitage, P. A., Santoscoy, P. L. M., Cunliffe, V. T., Huisken, J. et al. (2018). The effect of hyperglycemia on neurovascular coupling and cerebrovascular patterning in zebrafish. *J. Cereb. Blood Flow Metab.* 40, 298-313. 10.1177/0271678X18810615 30398083 PMC6985997

[DEV199720C12] Chi, N. C., Shaw, R. M., De Val, S., Kang, G., Jan, L. Y., Black, B. L. and Stainier, D. Y. R. (2008). Foxn4 directly regulates tbx2b expression and atrioventricular canal formation. *Genes Dev.* 22, 734-739. 10.1101/gad.1629408 18347092 PMC2275426

[DEV199720C13] Chico, T. J. A., Ingham, P. W. and Crossman, D. C. (2008). Modeling cardiovascular disease in the Zebrafish. *Trends Cardiovasc. Med.* 18, 150-155. 10.1016/j.tcm.2008.04.002 18555188

[DEV199720C14] Daetwyler, S., Günther, U., Modes, C. D., Harrington, K. and Huisken, J. (2019). Multi-sample SPIM image acquisition, processing and analysis of vascular growth in zebrafish. *Development* 146, dev173757. 10.1242/dev.173757 30824551 PMC6451323

[DEV199720C15] De Ménorval, M.-A., Mir, L. M., Fernández, M. L. and Reigada, R. (2012). Effects of dimethyl sulfoxide in cholesterol-containing lipid membranes: a comparative study of experiments in silico and with cells. *PLoS ONE* 7, e41733. 10.1371/journal.pone.0041733 22848583 PMC3404987

[DEV199720C16] Dreosti, E., Vendrell Llopis, N., Carl, M., Yaksi, E. and Wilson, S. W. (2014). Left-right asymmetry is required for the habenulae to respond to both visual and olfactory stimuli. *Curr. Biol.* 24, 440-445. 10.1016/j.cub.2014.01.016 24508167 PMC3969106

[DEV199720C17] Ellis, L. M. and Hicklin, D. J. (2008). VEGF-targeted therapy: mechanisms of anti-tumour activity. *Nat. Rev. Cancer* 8, 579-591. 10.1038/nrc2403 18596824

[DEV199720C18] Ferreira, T. A., Blackman, A. V., Oyrer, J., Jayabal, S., Chung, A. J., Watt, A. J., Sjöström, P. J. and Van Meyel, D. J. (2014). Neuronal morphometry directly from bitmap images. *Nat. Methods* 11, 982-984. 10.1038/nmeth.3125 25264773 PMC5271921

[DEV199720C19] Geling, A., Steiner, H., Willem, M., Bally-Cuif, L. and Haass, C. (2002). A gamma-secretase inhibitor blocks Notch signaling in vivo and causes a severe neurogenic phenotype in zebrafish. *EMBO Rep.* 3, 688-694. 10.1093/embo-reports/kvf124 12101103 PMC1084181

[DEV199720C20] Gerhardt, H., Golding, M., Fruttiger, M., Ruhrberg, C., Lundkvist, A., Abramsson, A., Jeltsch, M., Mitchell, C., Alitalo, K., Shima, D. et al. (2003). VEGF guides angiogenic sprouting utilizing endothelial tip cell filopodia. *J. Cell Biol.* 161, 1163-1177. 10.1083/jcb.200302047 12810700 PMC2172999

[DEV199720C21] Geudens, I., Herpers, R., Hermans, K., Segura, I., Ruiz De Almodovar, C., Bussmann, J., De Smet, F., Vandevelde, W., Hogan, B. M., Siekmann, A. et al. (2010). Role of delta-like-4/Notch in the formation and wiring of the lymphatic network in zebrafish. *Arterioscler. Thromb. Vasc. Biol.* 30, 1695-1702. 10.1161/ATVBAHA.110.203034 20466977 PMC5497575

[DEV199720C22] Glass, C. A., Perrin, R. M., Pocock, T. M. and Bates, D. O. (2006). Transient osmotic absorption of fluid in microvessels exposed to low concentrations of dimethyl sulfoxide. *Microcirculation* 13, 29-40. 10.1080/10739680500383464 16393944

[DEV199720C23] Guen, L. L., Karpanen, T., Schulte, D., Harris, N. C., Koltowska, K., Roukens, G., Bower, N. I., Van Impel, A., Stacker, S. A., Achen, M. G. et al. (2014). Ccbe1 regulates Vegfc-mediated induction of Vegfr3 signaling during embryonic lymphangiogenesis. *Development* 141, 1239-1249. 10.1242/dev.100495 24523457

[DEV199720C24] Gut, P., Reischauer, S., Stainier, D. Y. R. and Arnaout, R. (2017). Little fish, big data: zebrafish as a model for cardiovascular and metabolic disease. *Physiol. Rev.* 97, 889-938. 10.1152/physrev.00038.2016 28468832 PMC5817164

[DEV199720C25] Haixiang, G., Yijing, L., Shang, J., Mingyun, G., Yuanyue, H. and Bing, G. (2017). Learning from class-imbalanced data: Review of methods and applications. *Expert Syst. Appl.* 73, 220-239. 10.1016/j.eswa.2016.12.035

[DEV199720C26] Hogan, B. M. and Schulte-Merker, S. (2017). How to plumb a pisces: understanding vascular development and disease using Zebrafish embryos. *Dev. Cell* 42, 567-583. 10.1016/j.devcel.2017.08.015 28950100

[DEV199720C27] Hogan, B. M., Bos, F. L., Bussmann, J., Witte, M., Chi, N. C., Duckers, H. J. and Schulte-Merker, S. (2009). Ccbe1 is required for embryonic lymphangiogenesis and venous sprouting. *Nat. Genet.* 41, 396-398. 10.1038/ng.321 19287381

[DEV199720C28] Huisken, J., Swoger, J., Del Bene, F., Wittbrodt, J. and Stelzer, E. H. K. (2004). Optical sectioning deep inside live embryos by selective plane illumination microscopy. *Science* 305, 1007-1009. 10.1126/science.1100035 15310904

[DEV199720C29] Jenett, A., Schindelin, J. E. and Heisenberg, M. (2006). The Virtual Insect Brain protocol: creating and comparing standardized neuroanatomy. *BMC Bioinformatics* 7, 544. 10.1186/1471-2105-7-544 17196102 PMC1769402

[DEV199720C30] Kenney, J. W., Steadman, P. E., Young, O., Shi, M. T., Polanco, M., Dubaishi, S., Covert, K., Mueller, T. and Frankland, P. W. (2021). AZBA: a 3D adult Zebrafish brain atlas for the digital age. *Elife* 10, e69988. 10.7554/eLife.69988 34806976 PMC8639146

[DEV199720C31] Kovács, M., Tóth, J., Hetényi, C., Málnási-Csizmadia, A. and Sellers, J. R. (2004). Mechanism of blebbistatin inhibition of myosin II. *J. Biol. Chem* 279, 35557-35563. 10.1074/jbc.M405319200 15205456

[DEV199720C32] Kugler, E., Chico, T. and Armitage, P. (2018). Image analysis in light sheet fluorescence microscopy images of transgenic zebrafish vascular development. In *Medical Image Understanding and Analysis. MIUA 2018. vol. Communications in Computer and Information Science*, Vol. 894 (ed. M. Nixon, S. Mahmoodi and R. Zwiggelaar), pp. 343-353. Cham: Springer.

[DEV199720C33] Kugler, E., Plant, K., Chico, T. and Armitage, P. (2019a). Enhancement and segmentation workflow for the developing zebrafish vasculature. *J. Imaging* 5, 14. 10.3390/jimaging5010014 34465714 PMC8320862

[DEV199720C34] Kugler, E. C., Lessen, M., Daetwyler, S., Chhabria, K., Savage, A. M., Silva, V., Plant, K., Macdonald, R. B., Huisken, J., Wilkinson, R. N. et al. (2019b). Cerebrovascular endothelial cells form transient Notch-dependent cystic structures in zebrafish. *EMBO Rep.* 20, e47047. 10.15252/embr.201847047 31379129 PMC6680135

[DEV199720C35] Kugler, E., Chico, T. and Armitage, P. A. (2020a). Validating segmentation of the zebrafish vasculature. In *Medical Image Understanding and Analysis* (ed. Y. Zheng, B. M. Williams and K. Chen), pp. 270-281. Springer International Publishing.

[DEV199720C36] Kugler, E. C., Rampun, A., Chico, T. and Armitage, P. (2020b). *Segmentation of the Zebrafish Brain Vasculature from Light Sheet Fluorescence Microscopy Datasets*. *bioRxiv* 10.1101/2020.07.21.213843

[DEV199720C37] Kunst, M., Laurell, E., Mokayes, N., Kramer, A., Kubo, F., Fernandes, A. M., Förster, D., Dal Maschio, M. and Baier, H. (2019). A cellular-resolution atlas of the larval Zebrafish brain. *Neuron* 103, 21-38.e5. 10.1016/j.neuron.2019.04.034 31147152

[DEV199720C38] Lee, T. C., Kashyap, R. L. and Chu, C. N. (1994). Building skeleton models via 3-D medial surface axis thinning algorithms. *"CVGIP, Graph. Models Image Process."* 56, 462-478. 10.1006/cgip.1994.1042

[DEV199720C39] Legland, D., Arganda-Carreras, I. and Andrey, P. (2016). MorphoLibJ: integrated library and plugins for mathematical morphology with ImageJ. *Bioinformatics* 32, 3532-3534. 10.1093/bioinformatics/btw413 27412086

[DEV199720C40] Leslie, J. D., Ariza-Mcnaughton, L., Bermange, A. L., Mcadow, R., Johnson, S. L. and Lewis, J. (2007). Endothelial signalling by the Notch ligand Delta-like 4 restricts angiogenesis. *Development* 134, 839-844. 10.1242/dev.003244 17251261

[DEV199720C41] Lobov, I. B., Renard, R. A., Papadopoulos, N., Gale, N. W., Thurston, G., Yancopoulos, G. D. and Wiegand, S. J. (2007). Delta-like ligand 4 (Dll4) is induced by VEGF as a negative regulator of angiogenic sprouting. *Proc. Natl. Acad. Sci. USA* 104, 3219-3224. 10.1073/pnas.0611206104 17296940 PMC1805530

[DEV199720C42] Longair, M. H., Baker, D. A. and Armstrong, J. D. (2011). Simple Neurite Tracer: open source software for reconstruction, visualization and analysis of neuronal processes. *Bioinformatics* 27, 2453-2454. 10.1093/bioinformatics/btr390 21727141

[DEV199720C43] Lowe, D. (1999). Object recognition from local scale-invariant features. *Proceedings of the Seventh IEEE International Conference on Computer Vision* 2, 1150-1157. 10.1109/ICCV.1999.790410

[DEV199720C44] Lowe, D. G. (2004). Distinctive image features from scale-invariant keypoints. *Int. J. Comput. Vis.* 60, 91-110. 10.1023/B:VISI.0000029664.99615.94

[DEV199720C45] Marquart, G. D., Tabor, K. M., Horstick, E. J., Brown, M., Geoca, A. K., Polys, N. F., Nogare, D. D. and Burgess, H. A. (2017). High-precision registration between zebrafish brain atlases using symmetric diffeomorphic normalization. *Gigascience* 6, 1-15. 10.1093/gigascience/gix056 PMC5597853 28873968

[DEV199720C46] Metsalu, T. and Vilo, J. (2015). ClustVis: a web tool for visualizing clustering of multivariate data using Principal Component Analysis and heatmap. *Nucleic Acids Res.* 43, W566-W570. 10.1093/nar/gkv468 25969447 PMC4489295

[DEV199720C47] Meyer, A. and Schartl, M. (1999). Gene and genome duplications in vertebrates: the one-to-four (-to-eight in fish) rule and the evolution of novel gene functions. *Curr. Opin. Cell Biol.* 11, 699-704. 10.1016/S0955-0674(99)00039-3 10600714

[DEV199720C48] Morton, W. M., Ayscough, K. R. and Mclaughlin, P. J. (2000). Latrunculin alters the actin-monomer subunit interface to prevent polymerization. *Nat. Cell Biol.* 2, 376-378. 10.1038/35014075 10854330

[DEV199720C49] Nakamura, K., Taguchi, E., Miura, T., Yamamoto, A., Takahashi, K., Bichat, F., Guilbaud, N., Hasegawa, K., Kubo, K., Fujiwara, Y. et al. (2006). KRN951, a highly potent inhibitor of vascular endothelial growth factor receptor tyrosine kinases, has antitumor activities and affects functional vascular properties. *Cancer Res.* 66, 9134-9142. 10.1158/0008-5472.CAN-05-4290 16982756

[DEV199720C50] Otsu, N. (1979). A threshold selection method from gray-level histograms. *Trans. Sys.Man.* 9, 62-66. 10.1109/TSMC.1979.4310076

[DEV199720C51] Phng, L.-K. and Gerhardt, H. (2009). Angiogenesis: a team effort coordinated by notch. *Dev. Cell* 16, 196-208. 10.1016/j.devcel.2009.01.015 19217422

[DEV199720C52] Quillien, A., Moore, J. C., Shin, M., Siekmann, A. F., Smith, T., Pan, L., Moens, C. B., Parsons, M. J. and Lawson, N. D. (2014). Distinct Notch signaling outputs pattern the developing arterial system. *Development* 141, 1544-1552. 10.1242/dev.099986 24598161 PMC4074308

[DEV199720C53] Rahman, M. M. and Davis, D. N. (2013). Addressing the class imbalance problem in medical datasets. *IJMLC*, 3, 224-228.

[DEV199720C54] Randlett, O., Wee, C. L., Naumann, E. A., Nnaemeka, O., Schoppik, D., Fitzgerald, J. E., Portugues, R., Lacoste, A. M. B., Riegler, C., Engert, F. et al. (2015). Whole-brain activity mapping onto a zebrafish brain atlas. *Nat. Methods* 12, 1039-1046. 10.1038/nmeth.3581 26778924 PMC4710481

[DEV199720C55] Sacharidou, A., Stratman, A. N. and Davis, G. E. (2012). Molecular mechanisms controlling vascular lumen formation in three-dimensional extracellular matrices. *CTO* 195, 122-143. 10.1159/000331410 21997121 PMC3325603

[DEV199720C56] Sato, Y., Nakajima, S., Shiraga, N., Atsumi, H., Yoshida, S., Koller, T., Gerig, G. and Kikinis, R. (1998). 3D multi-scale line filter for segmentation and visualization of curvilinear structures in medical images. *Med. Image Anal.* 2, 143-168. 10.1016/s1361-8415(98)80009-1 10646760

[DEV199720C57] Scallan, J., Huxley, V. H. and Korthuis, R. J. (2010). *Pathophysiology of Edema Formation. Capillary Fluid Exchange: Regulation, Functions, and Pathology*: Morgan & Claypool Life Sciences. 21452435

[DEV199720C58] Schindelin, J., Arganda-Carreras, I., Frise, E., Kaynig, V., Longair, M., Pietzsch, T., Preibisch, S., Rueden, C., Saalfeld, S., Schmid, B. et al. (2012). Fiji - an Open Source platform for biological image analysis. *Nat. Methods* 9, 676-682. 10.1038/nmeth.2019 22743772 PMC3855844

[DEV199720C59] Schmid, B. (2010). *Computational Tools for the Segmentation and Registration of Confocal Brain Images of Drosophila Melanogaster*. Bayerischen Julius-Maximilians-Universitaet Wuerzburg.

[DEV199720C60] Sehnert, A. J., Huq, A., Weinstein, B. M., Walker, C., Fishman, M. and Stainier, D. Y. R. (2002). Cardiac troponin T is essential in sarcomere assembly and cardiac contractility. *Nat. Genet* 31, 106-110. 10.1038/ng875 11967535

[DEV199720C61] Severinghaus, J. W. (1995). Hypothetical roles of angiogenesis, osmotic swelling, and ischemia in high-altitude cerebral edema. *J. Appl. Physiol* 79, 375-379. 10.1152/jappl.1995.79.2.375 7592190

[DEV199720C62] Sholl, D. A. (1953). Dendritic organization in the neurons of the visual and motor cortices of the cat. *J. Anat* 87, 387-406. 10.1038/171387a0 13117757 PMC1244622

[DEV199720C63] Taha, A. A. and Hanbury, A. (2015). Metrics for evaluating 3D medical image segmentation: analysis, selection, and tool. *BMC Med. Imaging* 15, 29. 10.1186/s12880-015-0068-x 26263899 PMC4533825

[DEV199720C64] Tam, S. J., Richmond, D. L., Kaminker, J. S., Modrusan, Z., Martin-Mcnulty, B., Cao, T. C., Weimer, R. M., Carano, R. A. D., Van Bruggen, N. and Watts, R. J. (2012). Death receptors DR6 and TROY regulate brain vascular development. *Dev. Cell* 22, 403-417. 10.1016/j.devcel.2011.11.018 22340501

[DEV199720C65] Tetteh, G., Efremov, V., Forkert, N. D., Schneider, M., Kirschke, J., Weber, B., Zimmer, C., Piraud, M. and Menze, B. H. (2019). DeepVesselNet: vessel segmentation, centerline prediction, and bifurcation detection in 3-D angiographic volumes. *Front. Neurosci.* 14, 592352. 10.3389/fnins.2020.592352 PMC7753013 33363452

[DEV199720C66] Todorov, M. I., Paetzold, J. C., Schoppe, O., Tetteh, G., Shit, S., Efremov, V., Todorov-Völgyi, K., Düring, M., Dichgans, M., Piraud, M. et al. (2020). Machine learning analysis of whole mouse brain vasculature. *Nat. Methods* 17, 442-449. 10.1038/s41592-020-0792-1 32161395 PMC7591801

[DEV199720C67] Ullmann, J. F. P., Cowin, G., Kurniawan, N. D. and Collin, S. P. (2010). A three-dimensional digital atlas of the zebrafish brain. *Neuroimage* 51, 76-82. 10.1016/j.neuroimage.2010.01.086 20139016

[DEV199720C68] Wang, Z., Bovik, A. C., Sheikh, H. R. and Simoncelli, E. P. (2004). Image quality assessment: from error visibility to structural similarity. *IEEE Trans. Image Process.* 13, 600-612. 10.1109/TIP.2003.819861 15376593

[DEV199720C69] Watson, O., Novodvorsky, P., Gray, C., Rothman, A. M. K., Lawrie, A., Crossman, D. C., Haase, A., Mcmahon, K., Gering, M., Van Eeden, F. J. M. et al. (2013). Blood flow suppresses vascular Notch signalling via dll4 and is required for angiogenesis in response to hypoxic signalling. *Cardiovasc. Res.* 100, 252-261. 10.1093/cvr/cvt170 23812297 PMC3797625

[DEV199720C70] Westerfield, M. (1993). *The Zebrafish book: a guide for laboratory use of Zebrafish (Brachydanio rerio)*. University of Oregon Press.

