# Supplementary material for: Zebrafish vascular quantification: a tool for quantification of three-dimensional zebrafish cerebrovascular architecture by automated image analysis
Source: Development. 2022 Feb 14;149(3):dev199720. doi: 10.1242/dev.199720 (PMC8918806; doi:10.1242/dev.199720)
Supplement: Supplementary information [file develop-149-199720-s1.pdf]

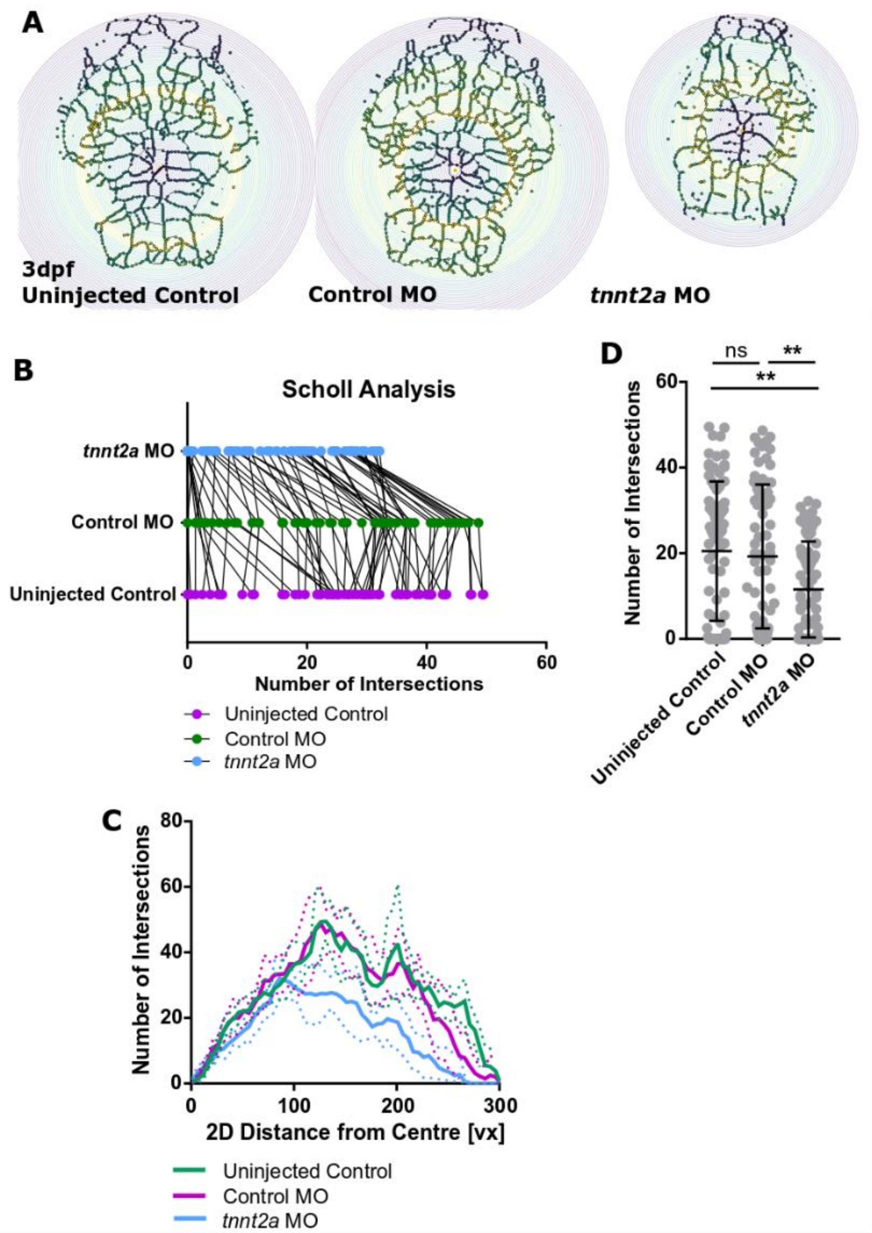
A
3dpf
Uninjected Control
Control MO
tnnt2a MO
B
Scholl Analysis
tnnt2a MO
Control MO
Uninjected Control
0
20
40
60
Number of Intersections
Uninjected Control
Control MO
tnnt2a MO
D
ns
**
**
Number of Intersections
60
40
20
0
Uninjected Control
Control MO
tnnt2a MO
C
80
60
40
20
0
Number of Intersections
0
100
200
300
2D Distance from Centre [vx]
Uninjected Control
Control MO
tnnt2a MO

Fig. S1. Scholl Analysis example.

A Scholl analysis, a method to originally quantify dendritic arbours, was used to assess branching of the cerebral vasculature. To achieve this, the centre-point of analysis was placed in skeleton MIPs between the left and right posterior communicating segment (exemplified by uninjected controls, control morphants and *tnnt2a* moprhants). From this centre-point, Scholl analysis is conducted by growing spheres (indicated by colours) and analysis of intersections (*ie* vessels) along those spheres.

B-D Scholl analysis therefore quantifies number of intersections and provides information on the 2D distance from the centre, allowing an analysis of complexity by number of intersections.

Development • Supplementary information

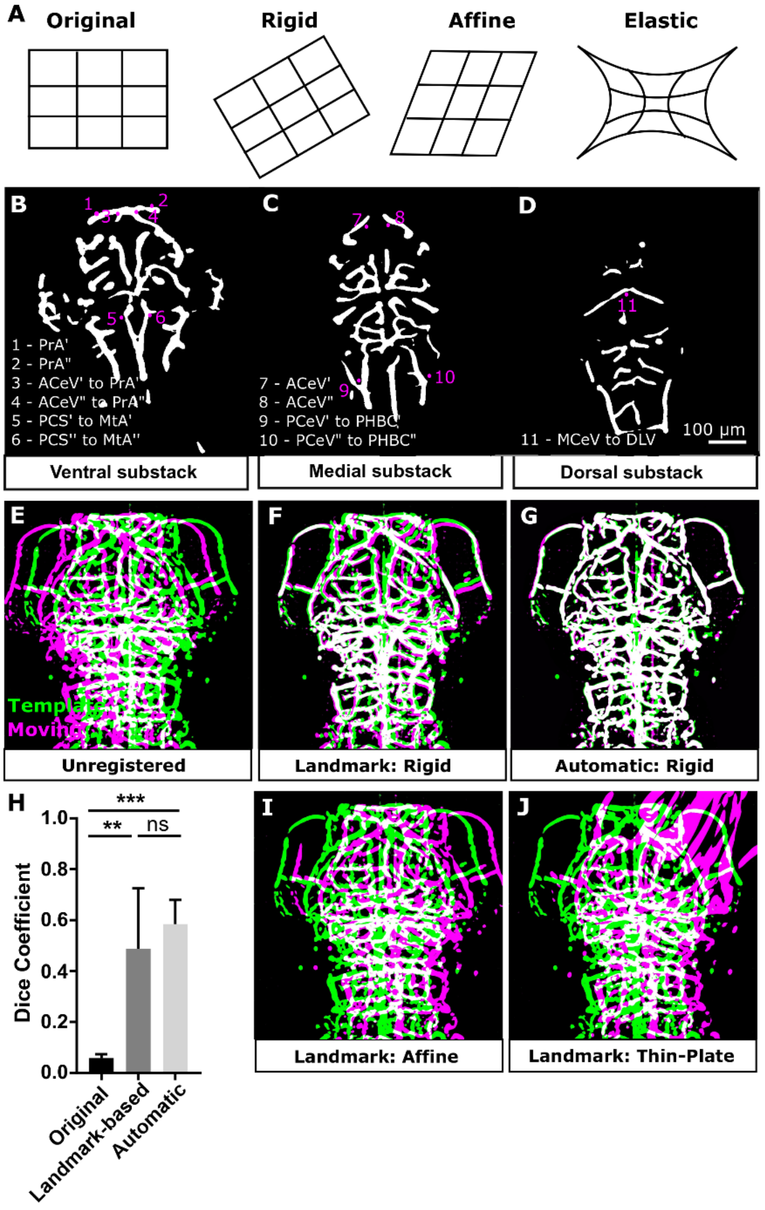
A
Original
Rigid
Affine
Elastic
B
C
D
1 - PrA'
2 - PrA"
3 - ACeV' to PrA'
4 - ACeV" to PrA"
5 - PCS' to MtA'
6 - PCS" to MtA"
7 - ACeV'
8 - ACeV"
9 - PCeV' to PHBC
10 - PCeV" to PHBC"
11 - MCeV to DLV
100 μm
Ventral substack
Medial substack
Dorsal substack
E
F
G
Template
Moving
Unregistered
Landmark: Rigid
Automatic: Rigid
H
Dice Coefficient
***
**
ns
Original
Landmark-based
Automatic
I
J
Landmark: Affine
Landmark: Thin-Plate

Fig. S2. Testing and validation of rigid landmark- or object-based inter-sample registration.

(A) The degree of freedom in registration methods determines the image transformations conducted. **(B-D)** Anatomical landmarks chosen for landmark-based rigid registration were as follows: (1) left prosencephalic artery (PrA'), (2) right PrA (PrA''), (3) junction point of left PrA and anterior cerebral vein (ACeV'), (4) junction point of right PrA'' and ACeV'', (5) junction of left posterior communicating segment (PCS') to metencephalic artery (MtA'), (6) junction of right PCS'' to MtA'', (7) highest curvature point of ACeV', (8) highest curvature point of ACeV'', (9) left posterior junction point of posterior cerebral vein (PCeV') and primordial hindbrain channel (PHBC'), (10) left posterior junction point pf PCeV'' and PHBC'', (11) junction point of middle cerebral vein (MCeV) and dorsal longitudinal vein (DLV). Positions are indicated in representative image.

E Same sample from two acquisitions overlaid following segmentation (green - template; magenta - moving image; white - overlap).

F Samples after rigid anatomical landmark-based registration.

G Samples after rigid automatic registration.

H Dice coefficient was statistically significantly increased after landmark-based (p 0.0017) and automatic registration (p 0.0003; n=5 embryos; 3 experimental repeats; One-Way ANOVA; mean ± s.d.).

I Samples after affine anatomical landmark-based registration.

J Samples after thin-plate anatomical landmark-based registration.

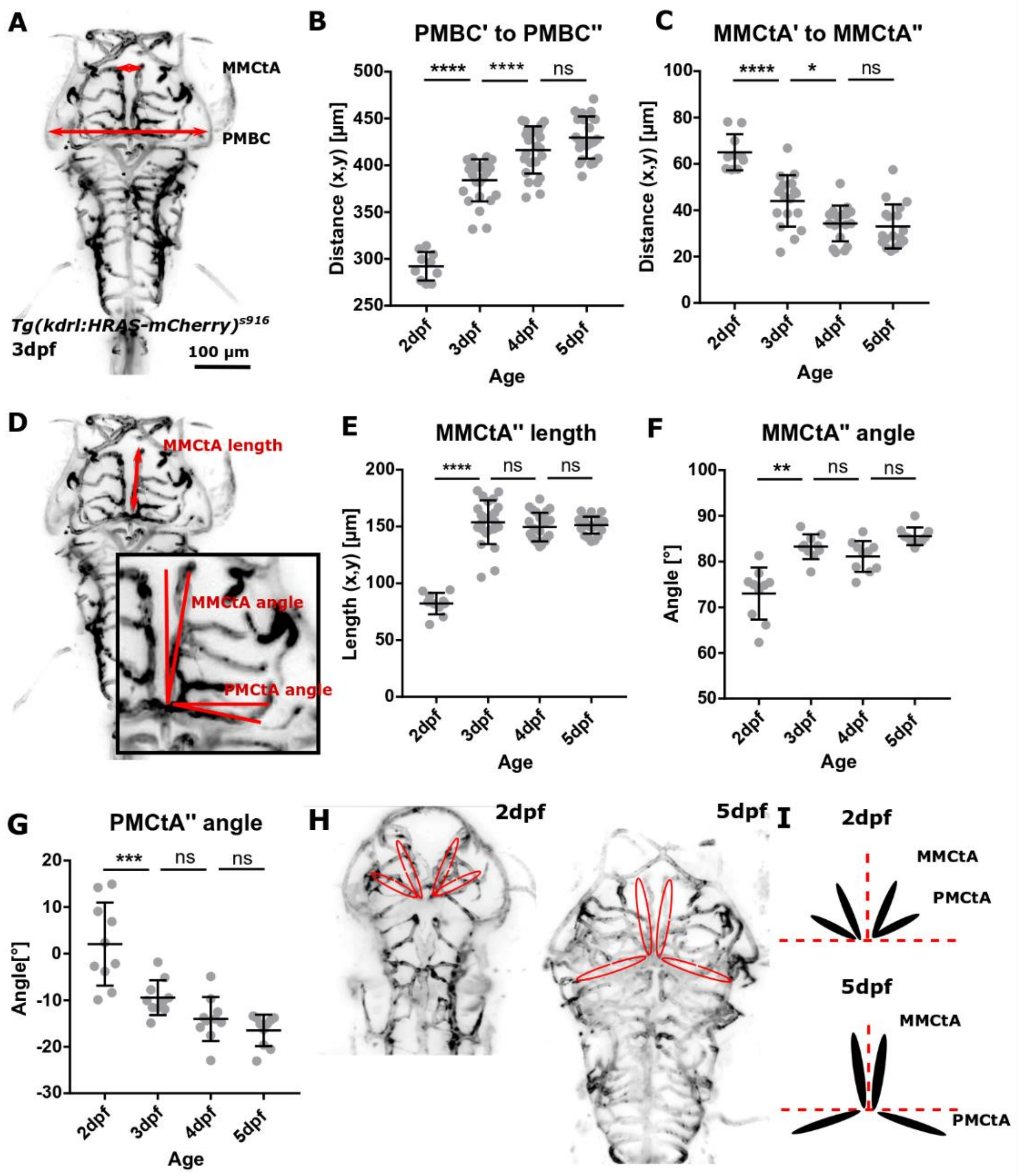

Fig. S3. Manual quantification allowed identification of anatomical landmarks for registration.

A Manual measurements were performed on the primordial midbrain channel (PMBC) and middle mesencephalic central artery (MMCtA).

B Distance between PMBC' and PMBC'' was found to statistically significantly increase from 2-to-4dpf (2-3dpf p<0.0001, 3-4dpf p<0.0001, 4-5dpf p 0.1786; embryos n 2dpf=10 (2 experimental repeats), 3dpf=25, 4dpf=25, 5dpf=25; 4 experimental repeats; One-way ANOVA; mean ± s.d.).

C Distance between anterior ends of MMCtA' and MMCtA'' was found to statistically significantly decrease from 2-to-4dpf (2-3dpf p<0.0001, 3-4dpf p 0.0137, 4-5dpf p

Development • Supplementary information

0.9739; embryos n 2dpf=10 (2 experimental repeats), 3dpf=18, 4dpf=18, 5dpf=18; 3 experimental repeats; One-way ANOVA; mean ± s.d.).

D Manual measurements were performed to measure MMCtA'' length, MMCtA'' angle, and PMCtA'' angle.

E MMCtA'' length was found to statistically significantly increase from 2-to-3dpf, but not 3-to-5dpf (2-3dpf $p<0.0001$, 3-4dpf p 0.7138, 4-5dpf p 0.9773; embryos n 2dpf=10 (2 experimental repeats), 3dpf=24, 4dpf=24, 5dpf=24; 4 experimental repeats; One-way ANOVA; mean ± s.d.).

F MMCtA'' angle was found to statistically significantly increase from 2-to-3dpf, but not 3-to-5dpf (2-3dpf p 0.0028, 3-4dpf $p>0.9999$, 4-5dpf p 0.0657; embryos n 2dpf=10, 3dpf=10, 4dpf=10, 5dpf=10; 2 experimental repeats; Kruskal-Wallis test; mean ± s.d.). **G** PMCtA'' angle was found to statistically significantly decrease from 2-to-3dpf, but not 3-to-5dpf (2-3dpf p 0.0003, 3-4dpf p 0.2790, 4-5dpf p 0.7652; embryos n 2dpf=10, 3dpf=10, 4dpf=10, 5dpf=10; 2 experimental repeats; One-way ANOVA; mean ± s.d.). **H,I** Data showed that MMCtA moved towards embryonic midline, whilst PMCtA moved posteriorly.

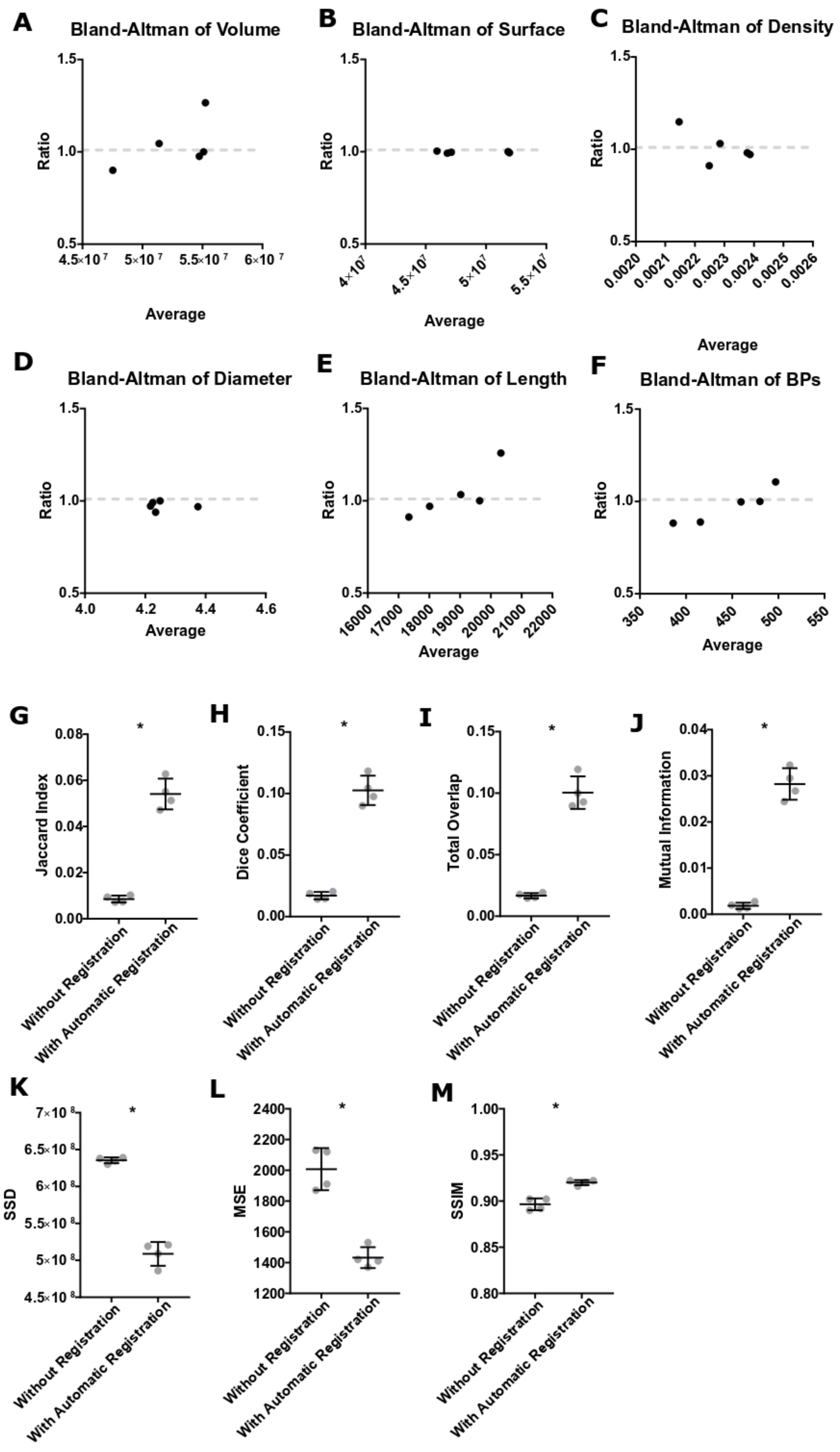

Development • Supplementary information

Fig. S4. Examining the impact of registration.

A Bland-Altman test of vascular volume pre- and post- automatic intersample-registration (n=5, 3dpf; bias 1.038, stdev 0.1381).

B Bland-Altman test of vascular surface pre- and post- automatic intersample-registration (bias 0.9974, stdev 0.00482).

C Bland-Altman test of vascular density pre- and post- automatic intersample-registration (bias 1.009, stdev 0.0889).

D Bland-Altman test of vascular diameter pre- and post- automatic intersample-registration (bias 0.9743, stdev 0.02364).

E Bland-Altman test of vascular length pre- and post- automatic intersample-registration (bias 1.035, stdev 0.1327).

F Bland-Altman test of vascular branching points pre- and post- automatic intersample-registration (bias 0.9751, stdev 0.0925).

G The Jaccard index was statistically significantly increased, following inter-sample registration (p 0.0286; two-tailed Mann-Whitney U test; mean ± s.d.).

H The Dice coefficient was statistically significantly increased, following inter-sample registration (p 0.0286; two-tailed Mann-Whitney U test; mean ± s.d.).

I The Total Overlap was statistically significantly increased, following inter-sample registration (p 0.0286; two-tailed Mann-Whitney U test; mean ± s.d.).

J The Mutual Information was statistically significantly increased, following inter-sample registration (p 0.0286; two-tailed Mann-Whitney U test; mean ± s.d.).

K The Sum of Squared Differences was statistically significantly decreased, following inter-sample registration (p 0.0286; two-tailed Mann-Whitney U test; mean ± s.d.).

L The Mean Square Error was statistically significantly decreased, following inter-sample registration (p 0.0286; two-tailed Mann-Whitney U test; mean ± s.d.).

M The Structural Similarity was statistically significantly increased, following inter-sample registration (p 0.0286; two-tailed Mann-Whitney U test; mean ± s.d.).

Development • Supplementary information

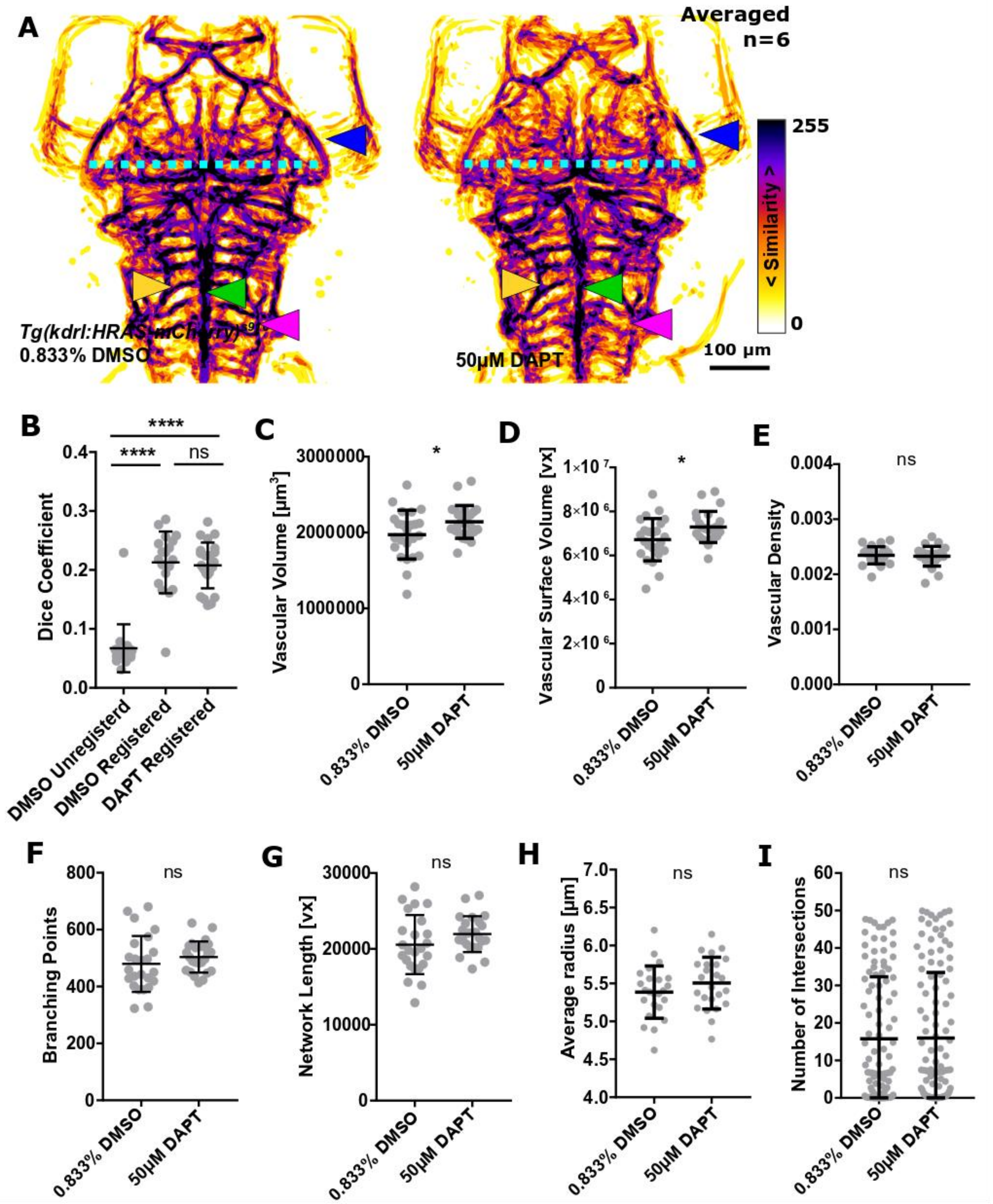

Fig. S5. The impact of Notch inhibition on vascular topology.

A MIPs of averaged data of control and DAPT treated samples following segmentation and registration showed no observable phenotype. *PMBC pattern (blue arrowhead), head size (PMBC'-to-PMBC'' distance; cyan dotted line), BA (green arrowhead), CtAs (yellow arrowhead), and PHBC (magenta arrowhead)*

B No statistically significant difference was found when comparing registered controls to Notch inhibitor treated samples (p>0.9999; control=19, DAPT=24; Kruskal-Wallis test; mean ± s.d.).

C Vascular volume was statistically significantly increased in DAPT treated samples (p 0.039; control=24, DAPT=24; unpaired t-test; mean ± s.d.).

D Vascular surface was statistically significantly decreased in DAPT treated samples (p 0.0226; unpaired two-tailed Student's t-test; mean ± s.d.).

E Vascular density was not statistically significantly changed in DAPT treated samples (p 0.8023; two-tailed Mann-Whitney U test; mean ± s.d.).

F Branching points were not statistically significantly changed in DAPT treated samples (p 0.2976; unpaired two-tailed Student's t-test; mean ± s.d.).

G Vascular network length was not statistically significantly changed in DAPT treated samples (p 0.1436; unpaired two-tailed Student's t-test; mean ± s.d.).

H Average vessel radius was not statistically significantly changed in DAPT treated samples (p 0.2334; unpaired two-tailed Student's t-test; mean ± s.d.).

I Vascular complexity was not statistically significantly changed in DAPT treated samples (p 0.8276; two-tailed Mann-Whitney U test; mean ± s.d.).

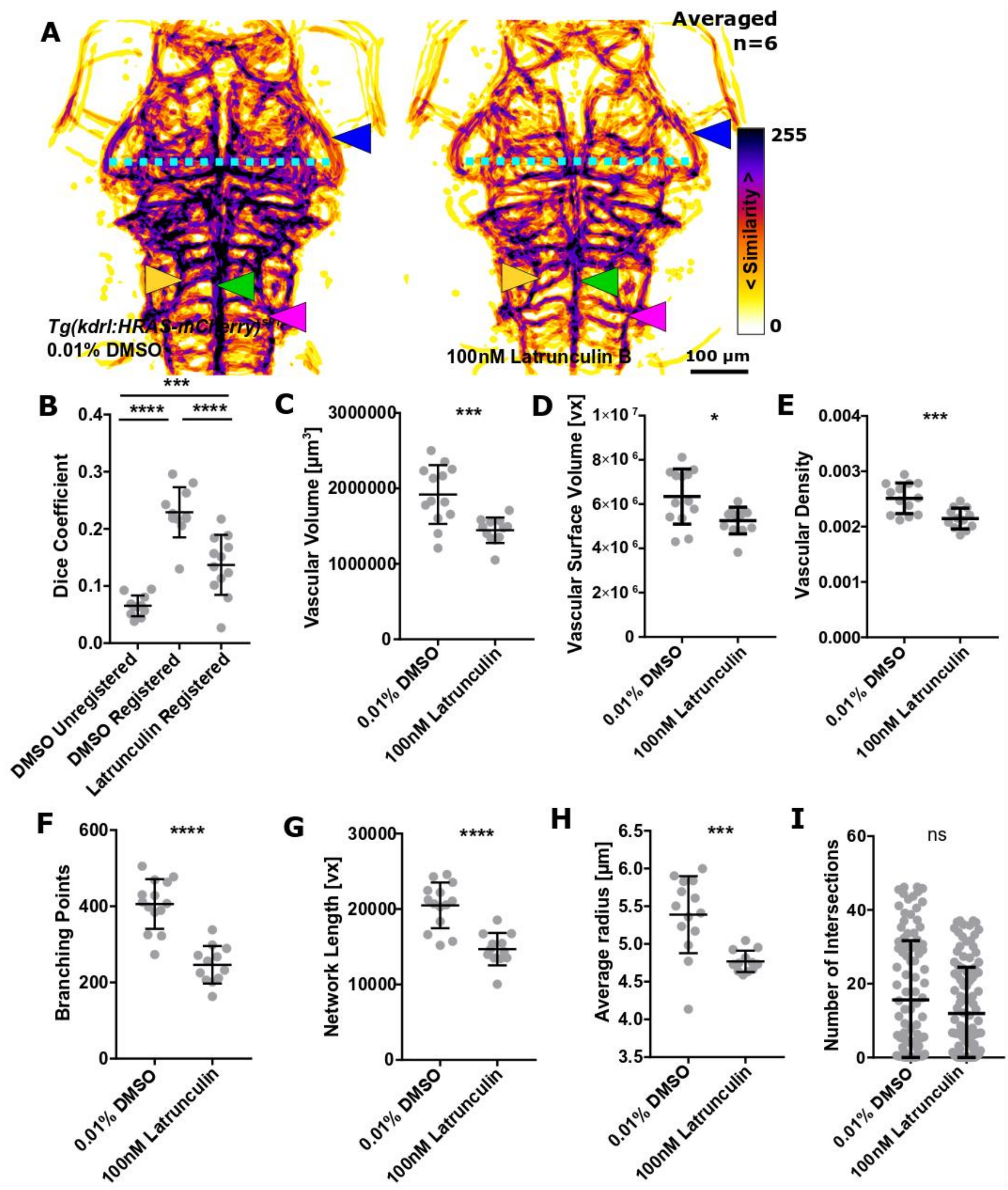

Development • Supplementary information

Fig. S6. The impact of actin polymerization inhibition on vascular topology. A MIPs of averaged data of control and Latrunculin B treated samples following segmentation and registration suggested overall high conservation of vessel patterning but altered in the intra-neural midbrain vessels and reduced vessel thickness. *PMBC pattern (blue arrowhead), head size (PMBC'-to-PMBC'' distance; cyan dotted line), BA (green arrowhead), CtAs (yellow arrowhead), and PHBC (magenta arrowhead)*

B A statistically significant reduction was found when comparing registered controls to F-actin inhibitor treated samples ($p<0.0001$; control=11, Latrunculin B=12; One-way ANOVA; mean ± s.d.).

C Vascular volume was statistically significantly decreased in Latrunculin B treated samples (p 0.0008; control=13, Latrunculin B=12; unpaired t-test; mean ± s.d.).

D Vascular surface was statistically significantly decreased in Latrunculin B treated samples (p 0.0119; unpaired t-test; mean ± s.d.).

E Vascular density was statistically significantly decreased in Latrunculin B treated samples (p 0.0008; unpaired two-tailed Student's t-test; mean ± s.d.).

F Branching points were statistically significantly decreased in Latrunculin B treated samples ($p<0.0001$; two-tailed Mann-Whitney U test; mean ± s.d.).

G Vascular network length was statistically significantly decreased in Latrunculin B treated samples ($p<0.0001$; two-tailed Mann-Whitney U test; mean ± s.d.).

H Average vessel radius was statistically significantly reduced in Latrunculin B treated samples (p 0.0002; two-tailed Mann-Whitney U test; mean ± s.d.).

I Vascular complexity was not statistically significantly changed in Latrunculin B treated samples (p 0.1829; two-tailed Mann-Whitney U test; mean ± s.d.).

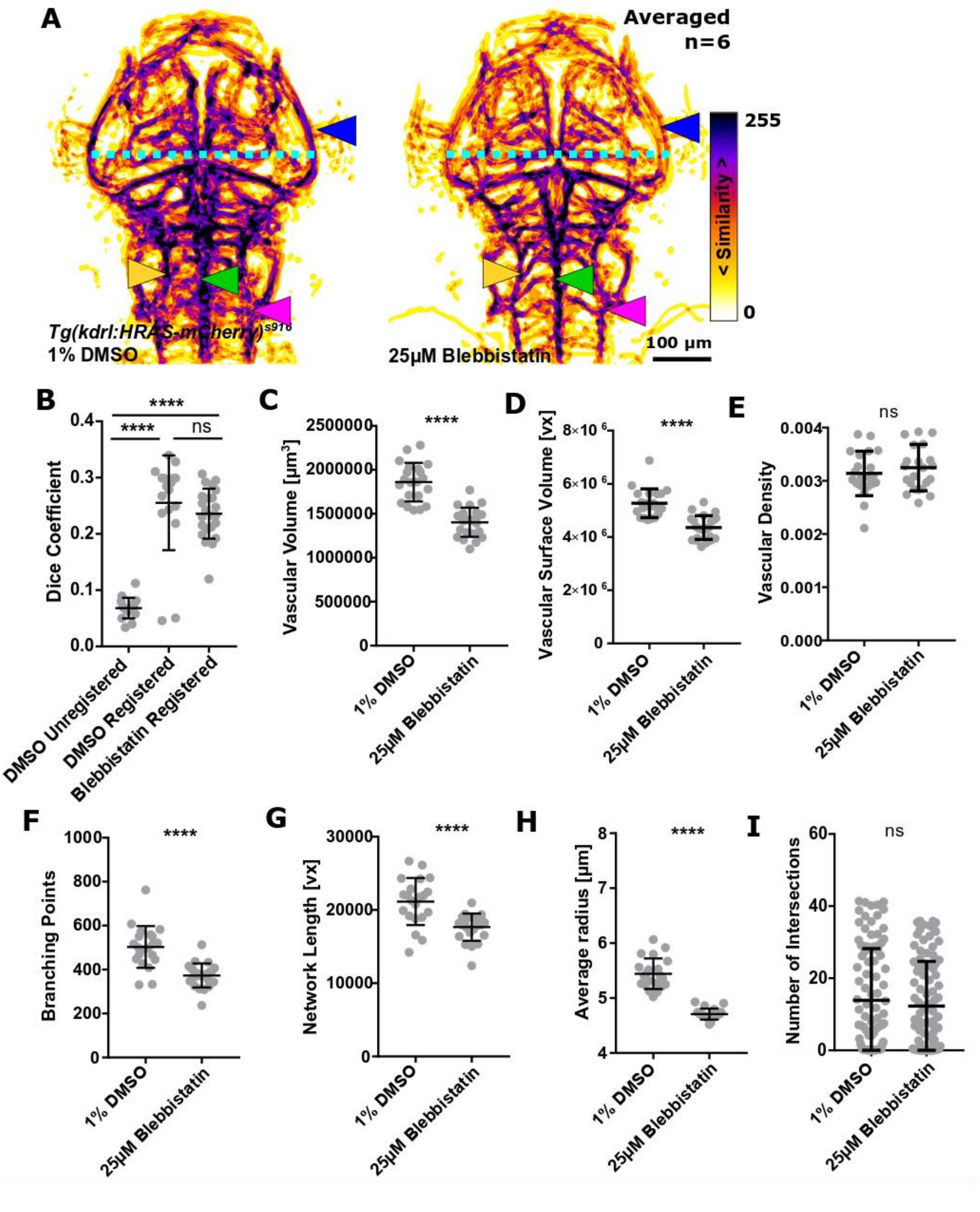

Development • Supplementary information

Fig. S7. The impact of Myosin II inhibition on vascular topology.
A MIPs of averaged data of control and Blebbistatin treated samples following segmentation and registration suggested overall high conservation of vessel patterning but altered in the intra-neural midbrain vessels and reduced vessel thickness. *PMBC pattern (blue arrowhead), head size (PMBC'-to-PMBC" distance; cyan dotted line), BA (green arrowhead), CtAs (yellow arrowhead), and PHBC (magenta arrowhead).*
B No statistically significant difference was found when comparing registered controls to Myosin II inhibitor treated samples (p 0.5737; control=17, Blebbistatin=23; Kruskal-Wallis test; mean ± s.d.).
C Vascular volume was statistically significantly decreased in Blebbistatin treated samples ($p<0.0001$; control=21, Blebbistatin=23; unpaired t-test; mean ± s.d.).
D Vascular surface was statistically significantly decreased in Blebbistatin treated samples ($p<0.0001$; two-tailed Mann-Whitney U test; mean ± s.d.).
E Vascular density was not statistically significantly changed in Blebbistatin treated samples (p 0.4097; unpaired two-tailed Student's t-test; mean ± s.d.).
F Branching points were statistically significantly decreased in Blebbistatin treated samples ($p<0.0001$; unpaired two-tailed Student's t-test; mean ± s.d.).
G Vascular network length was statistically significantly decreased in Blebbistatin treated samples ($p<0.0001$; two-tailed Mann-Whitney U test; mean ± s.d.).
H Average vessel radius was statistically significantly reduced in Blebbistatin treated samples ($p <0.0001$; unpaired two-tailed Student's t-test; mean ± s.d.).
I Vascular complexity was not statistically significantly changed in Blebbistatin treated samples (p 0.4909; two-tailed Mann-Whitney U test; mean ± s.d.).

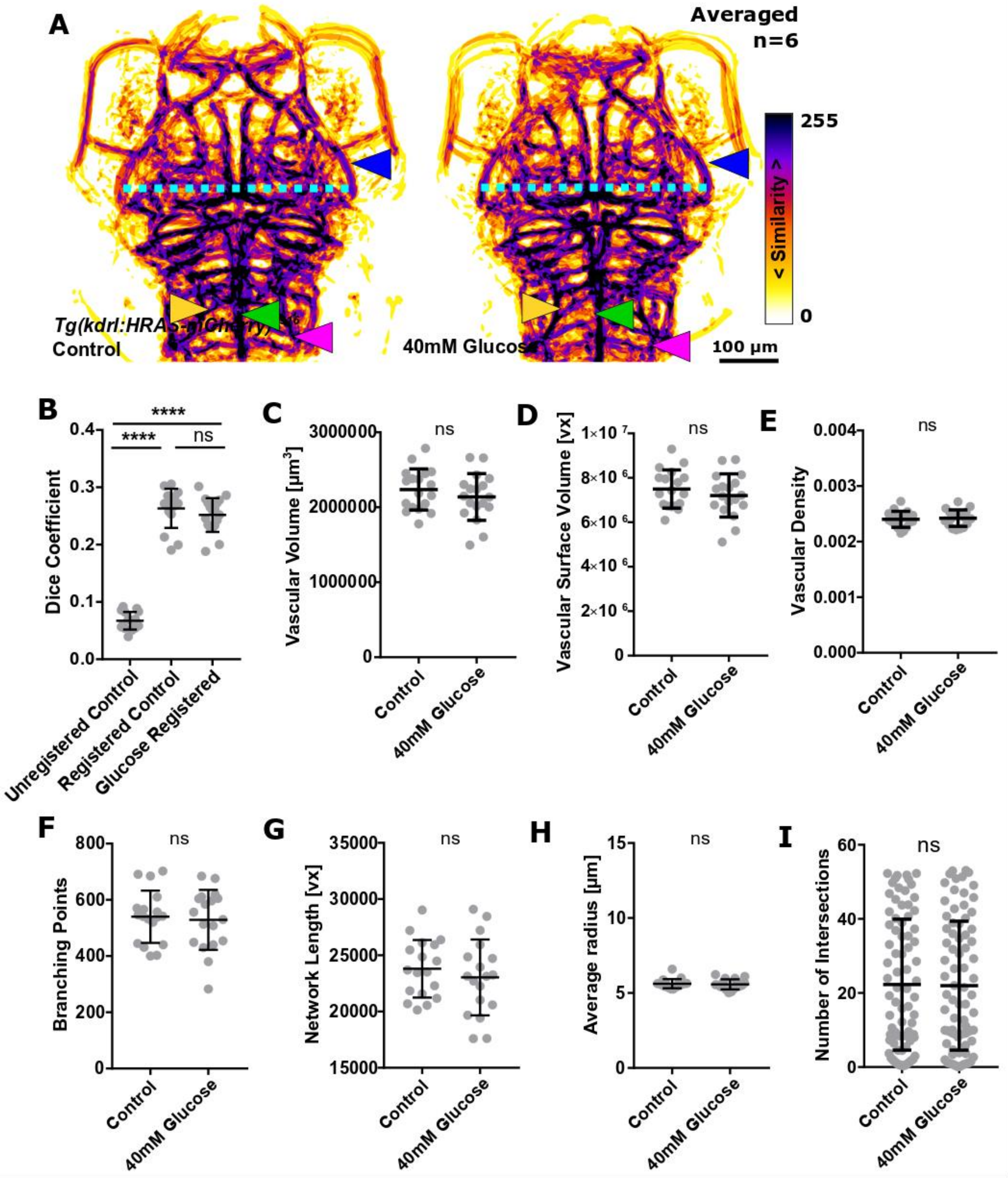

Development • Supplementary information

Figure S8. The impact of glucose increase on vascular topology.

A MIPs of averaged data of control and glucose treated samples following segmentation and registration showed no observable phenotype. *PMBC pattern (blue arrowhead), head size (PMBC'-to-PMBC'' distance; cyan dotted line), BA (green arrowhead), CtAs (yellow arrowhead), and PHBC (magenta arrowhead)*.

B No statistically significant difference was found when comparing registered controls to glucose treated samples (p 0.4559; control=16, glucose=18; One-way ANOVA; mean ± s.d.).

C Vascular volume was not statistically significantly changed in glucose treated samples (p 0.3183; control=18, glucose=18; unpaired two-tailed Student's t-test; mean ± s.d.).

D Vascular surface was not statistically significantly changed in glucose treated samples (p 0.3472; unpaired two-tailed Student's t-test; mean ± s.d.).

E Vascular density was not statistically significantly changed in glucose treated samples (p 0.6678; unpaired two-tailed Student's t-test; mean ± s.d.).

F Branching points were not statistically significantly changed in glucose treated samples (p 0.7434; unpaired two-tailed Student's t-test; mean ± s.d.).

G Vascular network length was not statistically significantly changed in glucose treated samples (p 0.4488; unpaired two-tailed Student's t-test; mean ± s.d.).

H Average vessel radius was not statistically significantly changed in glucose treated samples (p 0.7016; two-tailed Mann-Whitney U test; mean ± s.d.).

I Vascular complexity was not statistically significantly changed in glucose treated samples (p 0.9824; two-tailed Mann-Whitney U test; mean ± s.d.).

Development • Supplementary information

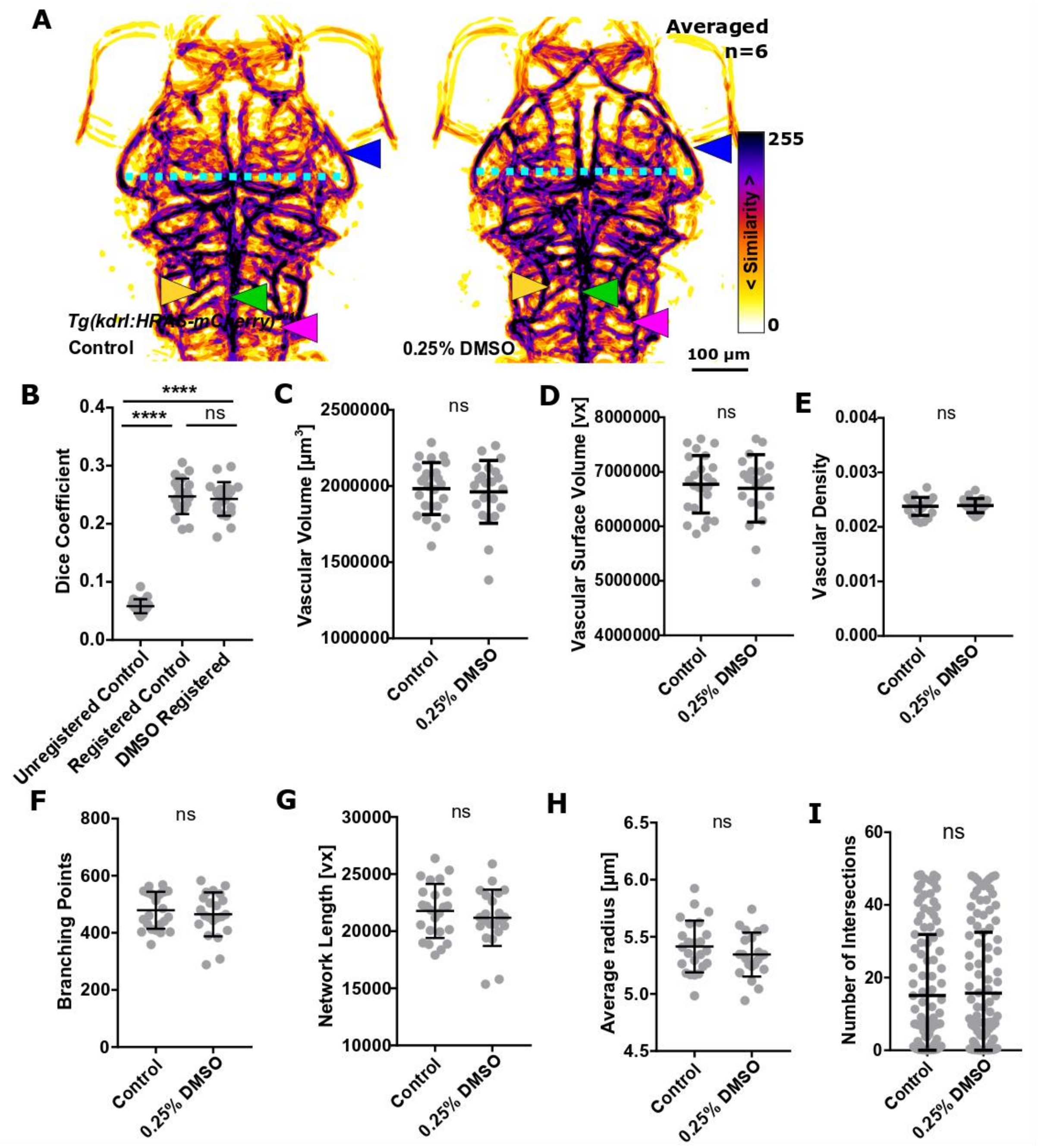

Development • Supplementary information

Fig. S9. The impact DMSO treatment on vascular topology.

A MIPs of averaged data of control and DMSO treated samples following segmentation and registration showed no observable phenotype. *PMBC pattern (blue arrowhead), head size (PMBC'-to-PMBC" distance; cyan dotted line), BA (green arrowhead), CtAs (yellow arrowhead), and PHBC (magenta arrowhead).*

B No statistically significant difference was found when comparing registered controls to DMSO treated samples (p 0.8399; control=21, DMSO=22; One-way ANOVA; mean ± s.d.).

C Vascular volume was not statistically significantly changed in DMSO treated samples (p 0.9047; control=24, DMSO=22; two-tailed Mann-Whitney U test; mean ± s.d.).

D Vascular surface was not statistically significantly changed in DMSO treated samples ($p>0.9999$; two-tailed Mann-Whitney U test; mean ± s.d.).

E Vascular density was not statistically significantly changed in DMSO treated samples (p 0.7566; unpaired two-tailed Student's t-test; mean ± s.d.).

F Branching points were not statistically significantly changed in DMSO treated samples (p 0.6909; two-tailed Mann-Whitney U test; mean ± s.d.).

G Vascular network length was not statistically significantly changed in DMSO treated samples (p 0.4015; unpaired two-tailed Student's t-test; mean ± s.d.).

H Average vessel radius was not statistically significantly changed in DMSO treated samples (p 0.2676; unpaired two-tailed Student's t-test; mean ± s.d.).

I Vascular complexity was not statistically significantly changed in DMSO treated samples (p 0.5631; two-tailed Mann-Whitney U test; mean ± s.d.).

Development • Supplementary information

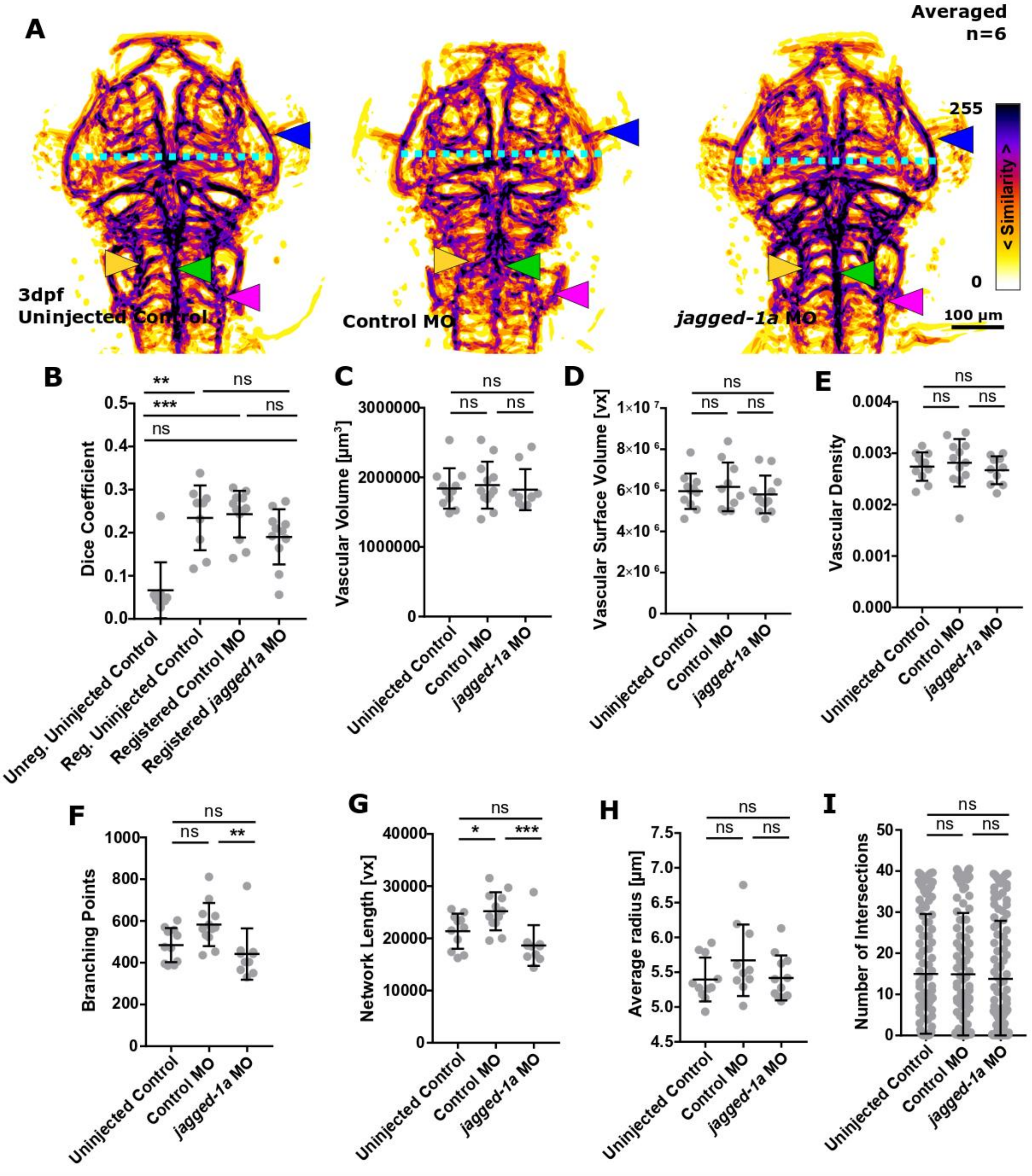
A
Averaged
n=6
255
< Similarity >
0
100 µm
3dpf
Uninjected Control
Control MO
jagged-1a MO
B
Dice Coefficient
Unreg. Uninjected Control
Reg. Uninjected Control
Registered Control MO
Registered jagged1a MO
C
Vascular Volume [µm³]
D
Vascular Surface Volume [vx]
E
Vascular Density
F
Branching Points
G
Network Length [vx]
H
Average radius [µm]
I
Number of Intersections
Uninjected Control
Control MO
jagged-1a MO
ns
**
***
*

Fig. S10. The impact jagged-1a knock-down on vascular topology.

A MIPs of averaged data of uninjected controls, control MO, and jagged-1a MO following segmentation and registration suggested subtle patterning alterations in intra-neural midbrain vessels. *PMBC pattern (blue arrowhead), head size (PMBC'-to-PMBC" distance; cyan dotted line), BA (green arrowhead), CtAs (yellow arrowhead), and PHBC (magenta arrowhead).*

B No statistically significant difference was found when comparing registered control MO to jagged-1a MO (p 0.6359; uninjected control=9, control MO=12, *jagged-1a* MO=10; Kruskal-Wallis test; mean ± s.d.).

C Vascular volume was not statistically significantly changed in jagged-1a MO (uninjected control p>0.9999, control MO p>0.9999; uninjected control=11, control MO=12, *jagged-1a* MO=10; Kruskal Wallis test; mean ± s.d.).

D Vascular surface was not statistically significantly changed in jagged-1a MO (uninjected control p 0.9254, control MO p 0.6702; One-Way ANOVA; mean ± s.d.).

E Vascular density was not statistically significantly changed in *jagged-1a* MO (uninjected control p 0.8905, control MO p 0.6131; One-Way ANOVA; mean ± s.d.).

F Branching points were statistically significantly decreased in *jagged-1a* MO in comparison to control MO (uninjected control p 0.6219, control MO p 0.0041; Kruskal-Wallis test; mean ± s.d.).

G Vascular network length was statistically significantly decreased in *jagged-1a* MO in comparison to control MO (uninjected control p 0.2140, control MO p 0.0006; One-Way ANOVA; mean ± s.d.).

H Average vessel radius was not statistically significantly changed in *jagged-1a* MO (uninjected control p 0.9876, control MO p 0.2966; One-Way ANOVA; mean ± s.d.).

I Vascular complexity was not statistically significantly changed in *jagged-1a* MO (uninjected control p>0.9999, control MO p>0.9999; Kruskal-Wallis test; mean ± s.d.).

Development • Supplementary information

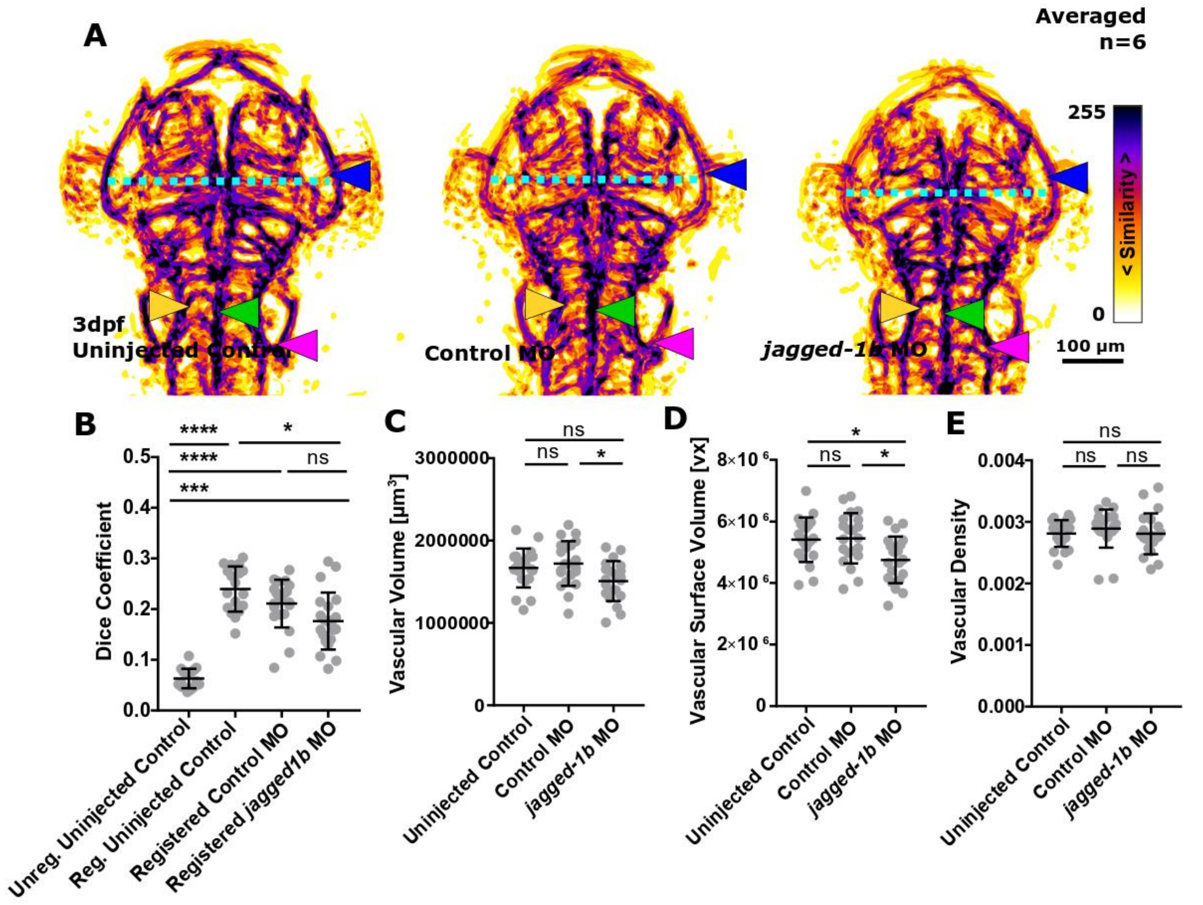
A
Averaged
n=6
255
< Similarity >
0
100 µm
3dpf
Uninjected Control
Control MO
jagged-1b MO
B
Dice Coefficient
Unreg. Uninjected Control
Reg. Uninjected Control
Registered Control MO
Registered jagged1b MO
C
Vascular Volume [µm³]
D
Vascular Surface Volume [vx]
E
Vascular Density
F
Branching Points
G
Network Length [vx]
H
Average radius [µm]
I
Number of Intersections
Uninjected Control
Control MO
jagged-1b MO

Fig. S11. The impact jagged-1b knock-down on vascular topology.

A MIPs of averaged data of uninjected controls, control MO, and jagged-1b MO following segmentation and registration suggested subtle patterning alterations in intra-neural midbrain vessels. *PMBC pattern (blue arrowhead), head size (PMBC'-to-PMBC" distance; cyan dotted line), BA (green arrowhead), CtAs (yellow arrowhead), and PHBC (magenta arrowhead).*

B No statistically significant difference was found when comparing registered control MO to *jagged-1b* MO (p 0.4737; uninjected control=18, control MO=21, *jagged-1b* MO=21; Kruskal-Wallis test; mean ± s.d.).

C Vascular volume was statistically significantly decreased in *jagged-1b* MO in comparison to control MO (uninjected control p 0.0848, control MO p 0.0222; uninjected control=21, control MO=21, *jagged-1b* MO=21; One-Way ANOVA; mean ± s.d.).

D Vascular surface was statistically significantly decreased in *jagged-1b* MO (uninjected control p 0.0204, control MO p 0.0124; One-Way ANOVA; mean ± s.d.).

E Vascular density was not statistically significantly changed in *jagged-1b* MO (uninjected control p>0.9999, control MO p 0.1694; One-Way ANOVA; mean ± s.d.).

F Branching points were not statistically significantly changed in *jagged-1b* MO (uninjected control p 0.2515, control MO p 0.1484; One-Way ANOVA; mean ± s.d.).

G Vascular network length was statistically significantly decreased in *jagged-1b* MO in comparison to control MO (uninjected control p 0.1339, control MO p 0.0082; One-Way ANOVA; mean ± s.d.).

H Average vessel radius was not statistically significantly changed in *jagged-1b* MO (uninjected control p 0.0922, control MO p 0.1601; One-Way ANOVA; mean ± s.d.).

I Vascular complexity was not statistically significantly changed in *jagged-1b* MO (uninjected control p >0.9999, control MO p 0.7584; Kruskal-Wallis test; mean ± s.d.).

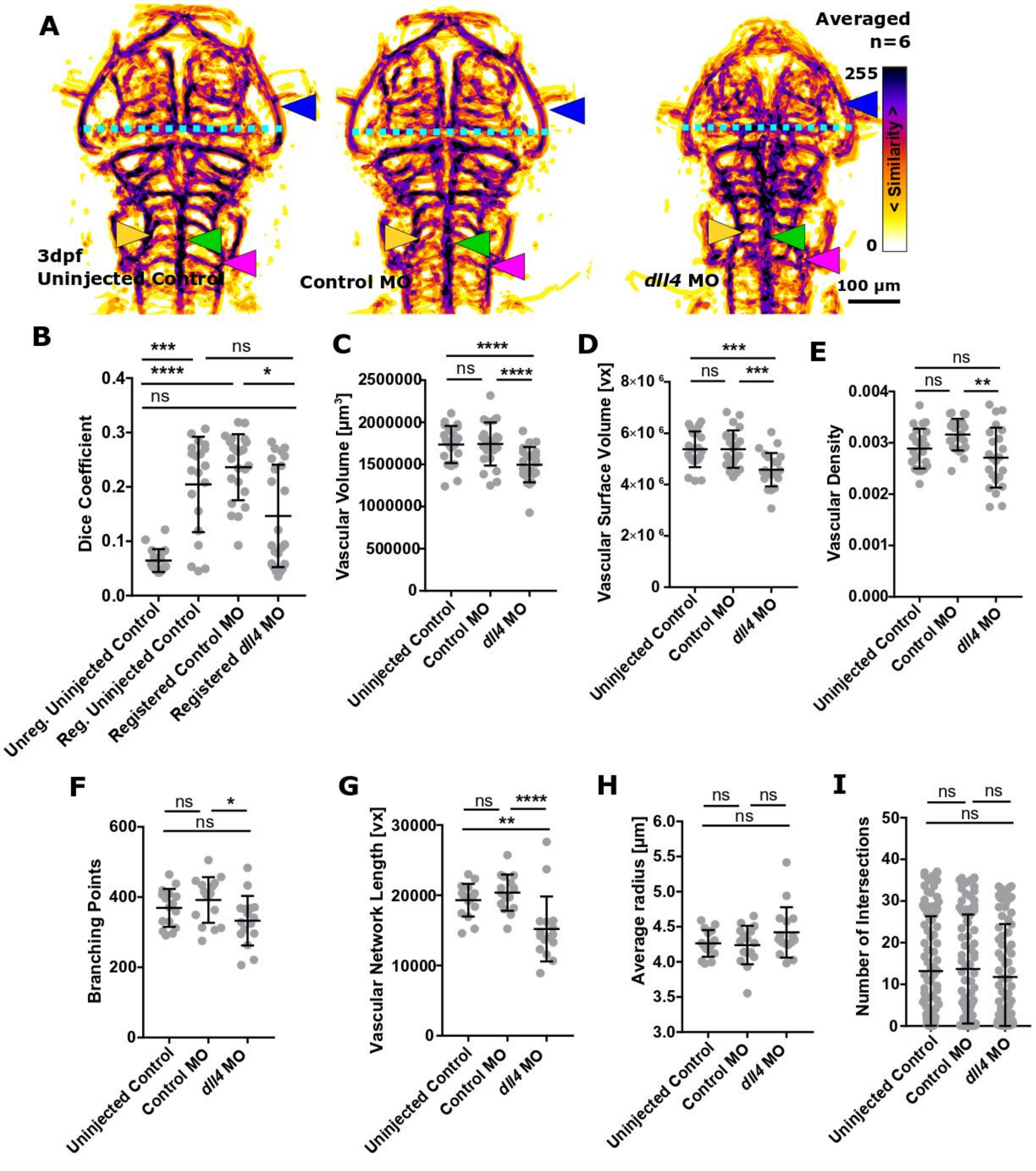
A
Averaged n=6
255
< Similarity >
0
3dpf Uninjected Control
Control MO
dll4 MO
100 µm
B
Dice Coefficient
Unreg. Uninjected Control
Reg. Uninjected Control
Registered Control MO
Registered dll4 MO
C
Vascular Volume [µm³]
D
Vascular Surface Volume [vx]
E
Vascular Density
F
Branching Points
G
Vascular Network Length [vx]
H
Average radius [µm]
I
Number of Intersections
Uninjected Control
Control MO
dll4 MO

Fig. S12. The impact dll4 knock-down on vascular topology.

A MIPs of averaged data of uninjected controls, control MO, and dll4 MO following segmentation and registration indicated *dll4* MO to have a smaller head size, with potential alterations patterning changes in the midbrain, but not hindbrain. *PMBC pattern (blue arrowhead), head size (PMBC'-to-PMBC'' distance; cyan dotted line), BA (green arrowhead), CtAs (yellow arrowhead), and PHBC (magenta arrowhead)*.

B A statistically significant decrease was found when comparing registered control MO to *dll4* MO (p 0.0103; uninjected control=20, control MO=23, dll4 MO=23; Kruskal-Wallis test; mean ± s.d.).

C Vascular volume was statistically significantly decreased in *dll4* MO (uninjected control p 0.0023; control MO p 0.0017; uninjected control=23, control MO=23, dll4 MO=23; One-Way ANOVA; mean ± s.d.).

D Vascular surface was statistically significantly decreased in *dll4* MO (uninjected control p 0.0007, control MO p 0.0006; One-Way ANOVA; mean ± s.d.).

E Vascular density was statistically significantly decreased in *dll4* MO in comparison to control MO (uninjected control p 0.3718, control MO p 0.0028; One-Way ANOVA; mean ± s.d.).

F Branching points were statistically significantly decreased in *dll4* MO in comparison to control MO (uninjected control p 0.4662, control MO p 0.0484; Kruskal-Wallis test; mean ± s.d.).

G Vascular network length was statistically significantly decreased in *dll4* MO (uninjected control p 0.0076, control MO p 0.0004; Kruskal-Wallis test; mean ± s.d.).

H Average vessel radius was not statistically significantly changed in *dll4* MO (uninjected control p 0.5547, control MO p 0.7673; Kruskal-Wallis test; mean ± s.d.).

I Vascular complexity was not statistically significantly changed in *dll4* MO (uninjected control p>0.9999, control MO p>0.9999; Kruskal-Wallis test; mean ± s.d.).

Development • Supplementary information

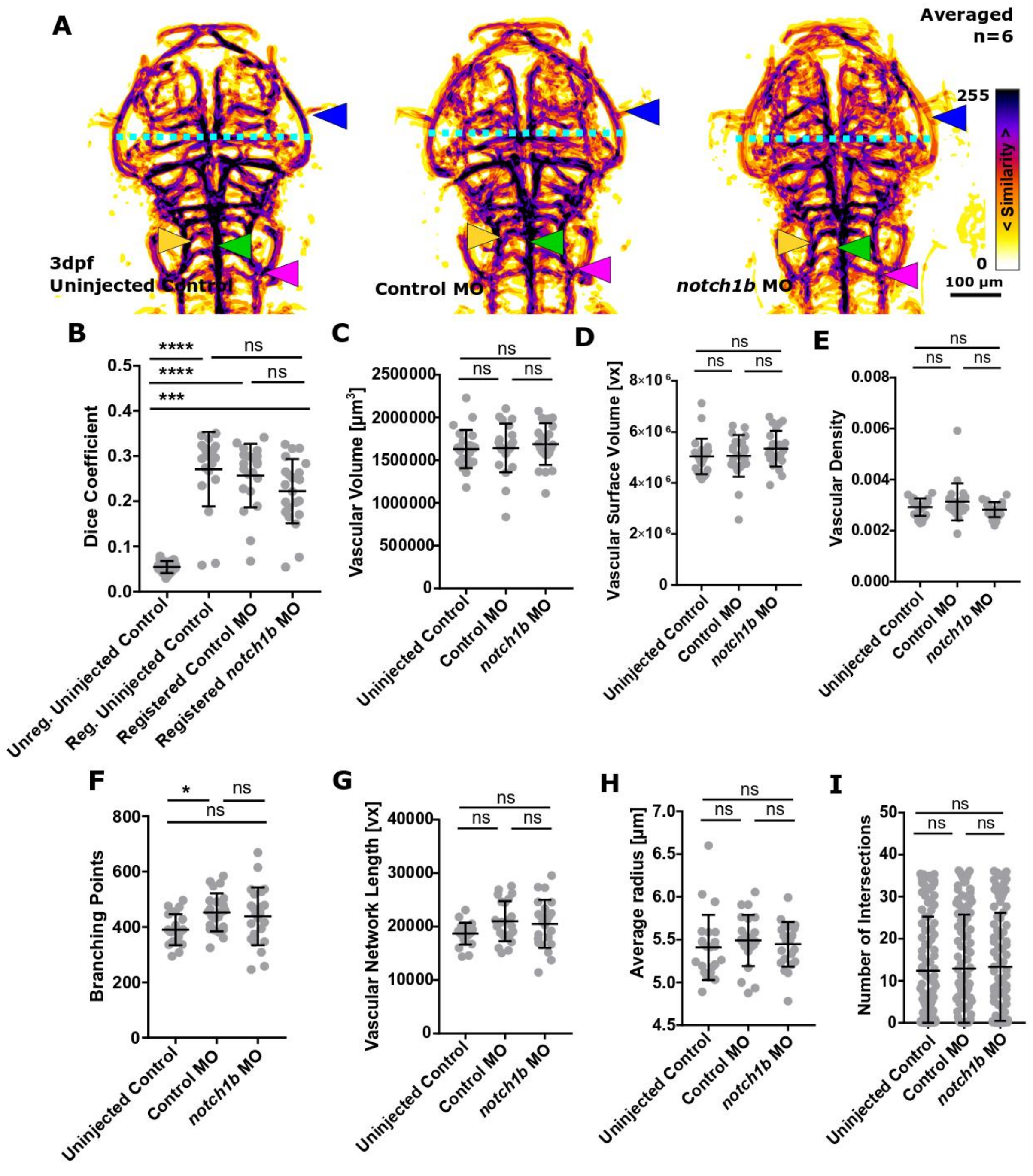

Development • Supplementary information

Fig. S13. The impact notch1b knock-down on vascular topology.

A MIPs of averaged data of uninjected controls, control MO, and notch1b MO following segmentation and registration showed no observable difference. *PMBC pattern (blue arrowhead), head size (PMBC'-to-PMBC'' distance; cyan dotted line), BA (green arrowhead), CtAs (yellow arrowhead), and PHBC (magenta arrowhead)*

B No statistically significant difference was found when comparing registered control MO to *notch1b* MO (p 0.9922; uninjected control=20, control MO=21, *notch1b* MO=23; Kruskal-Wallis test; mean ± s.d.).

C Vascular volume was not statistically significantly changed in *notch1b* MO (uninjected control p 0.9634, control MO p>0.9999; uninjected control=23, control MO=23, *notch1b* MO=23; Kruskal-Wallis test; mean ± s.d.).

D Vascular surface was not statistically significantly changed in *notch1b* MO (uninjected control p 0.2296, control MO p>0.9999; Kruskal-Wallis test; mean ± s.d.). **E** Vascular density was not statistically significantly changed in *notch1b* MO (uninjected control p 0.6589, control MO p 0.1010; Kruskal-Wallis test; mean ± s.d.). **F** Branching points were not statistically significantly changed in *notch1b* MO (uninjected control p 0.1018, control MO p 0.8225; One-Way ANOVA; mean ± s.d.). **G** Vascular network length was not statistically significantly changed in *notch1b* MO (uninjected control p 0.2077, control MO p 0.8787; One-Way ANOVA; mean ± s.d.). **H** Average vessel radius was not statistically significantly changed in *notch1b* MO (uninjected control p 0.7102, control MO p>0.9999; Kruskal-Wallis test; mean ± s.d.). **I** Vascular complexity was not statistically significantly changed in *notch1b* MO (uninjected control p>0.9999, control MO p>0.9999; Kruskal-Wallis test; mean ± s.d.).

Development • Supplementary information

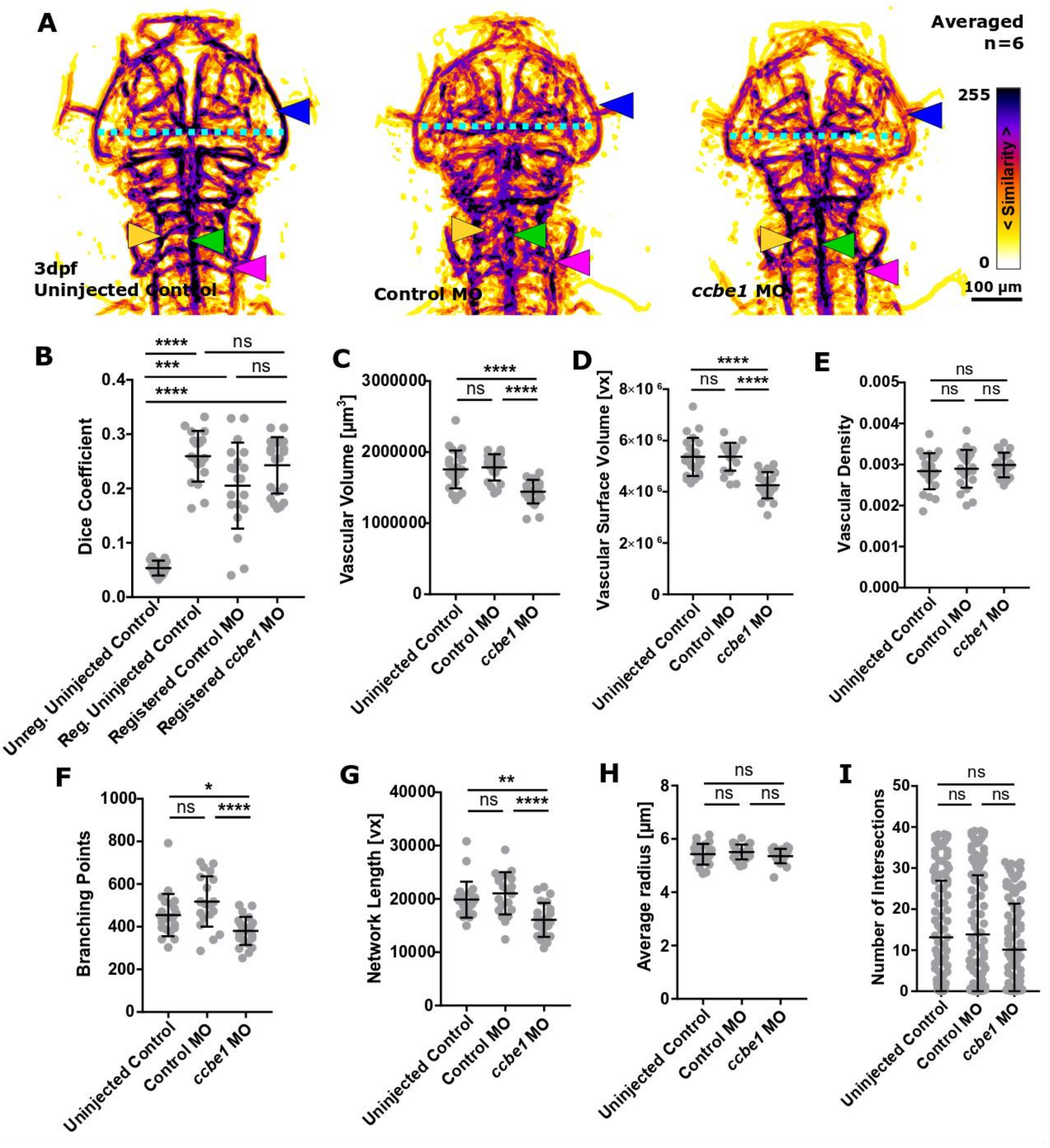

Fig. S14. The impact *ccbe1* knock-down on vascular topology.

A MIPs of averaged data of uninjected controls, control MO, and *ccbe1* MO following segmentation and registration suggested alterations of the midbrain vasculature. *PMBC pattern (blue arrowhead), head size (PMBC'-to-PMBC'' distance; cyan dotted line), BA (green arrowhead), CtAs (yellow arrowhead), and PHBC (magenta arrowhead)*

B No statistically significant difference was found when comparing registered control MO to *ccbe1* MO (p 0.9061; uninjected control=19, control MO=20, *ccbe1* MO=21; Kruskal-Wallis test; mean ± s.d.).

Development • Supplementary information

C Vascular volume was statistically significantly decreased in ccbe1 MO (uninjectedcontrol $p<0.0001$, control MO $p<0.0001$; uninjected control=23, control MO=21, *ccbe1* MO=23; One-Way ANOVA; mean ± s.d.).
D Vascular surface was statistically significantly decreased in *ccbe1* MO (uninjectedcontrol $p<0.0001$, control MO $p<0.0001$; One-Way ANOVA; mean ± s.d.).
E Vascular density was not statistically significantly changed in *ccbe1* MO (uninjectedcontrol p 0.4194, control MO p 0.7287; One-Way ANOVA; mean ± s.d.).
F Branching points were statistically significantly decreased in *ccbe1* MO (uninjectedcontrol p 0.0283, control MO $p<0.0001$; Kruskal-Wallis test; mean ± s.d.).
G Vascular network length was statistically significantly decreased in *ccbe1* MO(uninjected control p 0.0032, control MO $p<0.0001$; Kruskal-Wallis test; mean ± s.d.).
H Average vessel radius was not statistically significantly changed in *ccbe1* MO(uninjected control p 0.6170, control MO p 0.3152; One-Way ANOVA; mean ± s.d.).
I Vascular complexity was not statistically significantly changed in *ccbe1* MO(uninjected control p 0.8835, control MO p 0.6544; Kruskal-Wallis test; mean ± s.d.).

Development • Supplementary information

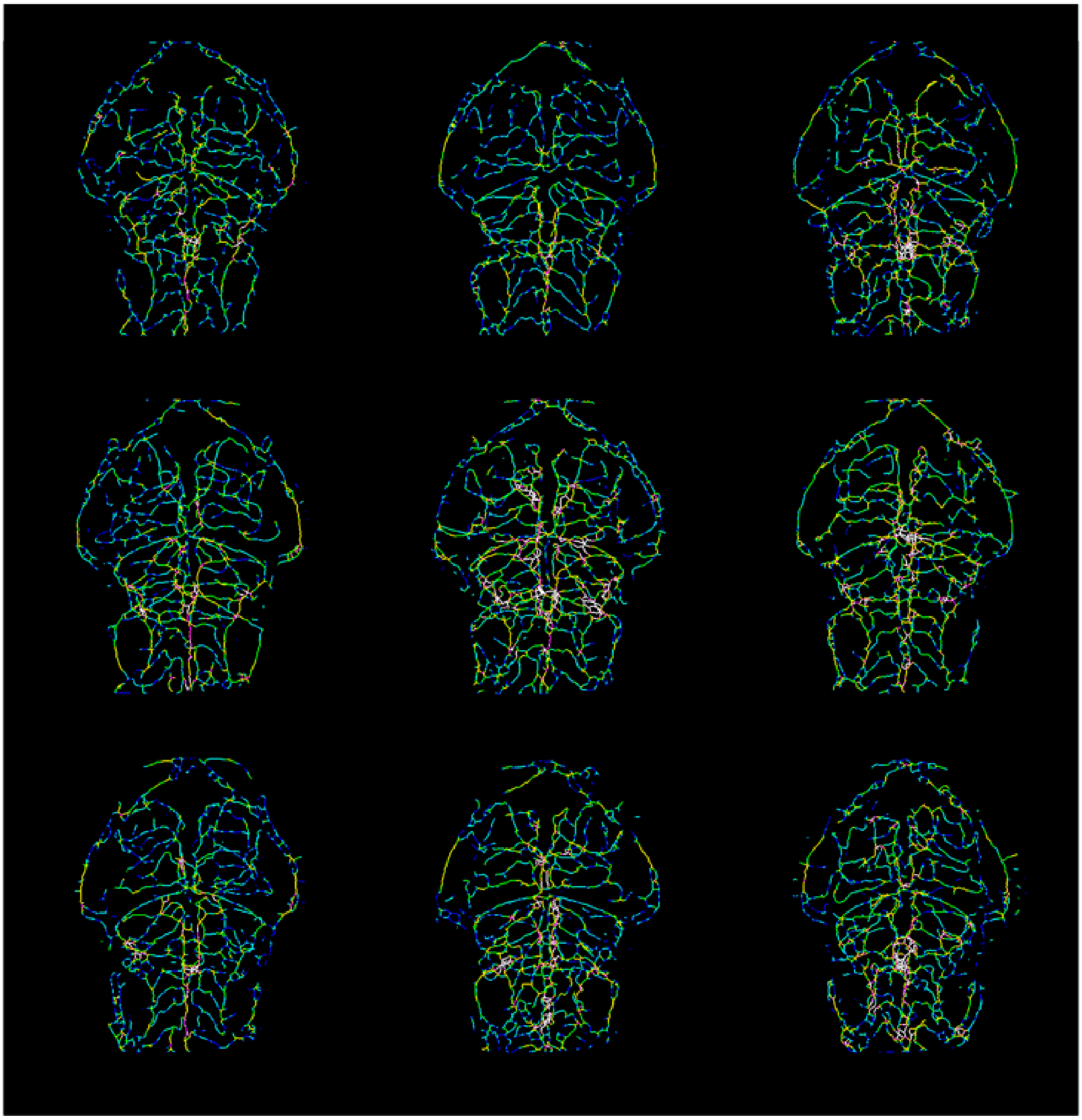

Fig. S15. Vascular radius information is saved as one-voxel-thick representations, allowing for further examination.

Image shows vessel radii on nine embryos.

Development • Supplementary information

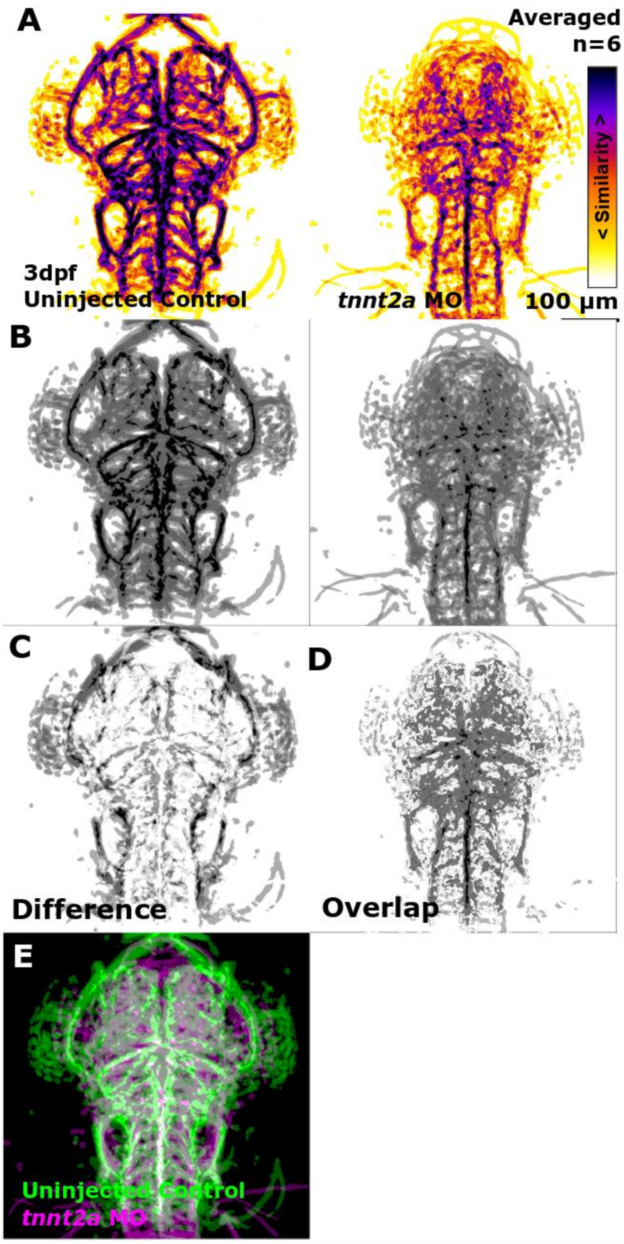

Fig. S16. Examination of regional similarity and variability.

A Six 3 dpf embryos, following segmentation, registration and averaging (LUT fire). **B** Grayscale of A.

C Difference between uninjected control and *tnnt2a* MO.

D Overlap between uninjected control and *tnnt2a* MO.

E MIP uninjected control and *tnnt2a* MO.

Development • Supplementary information

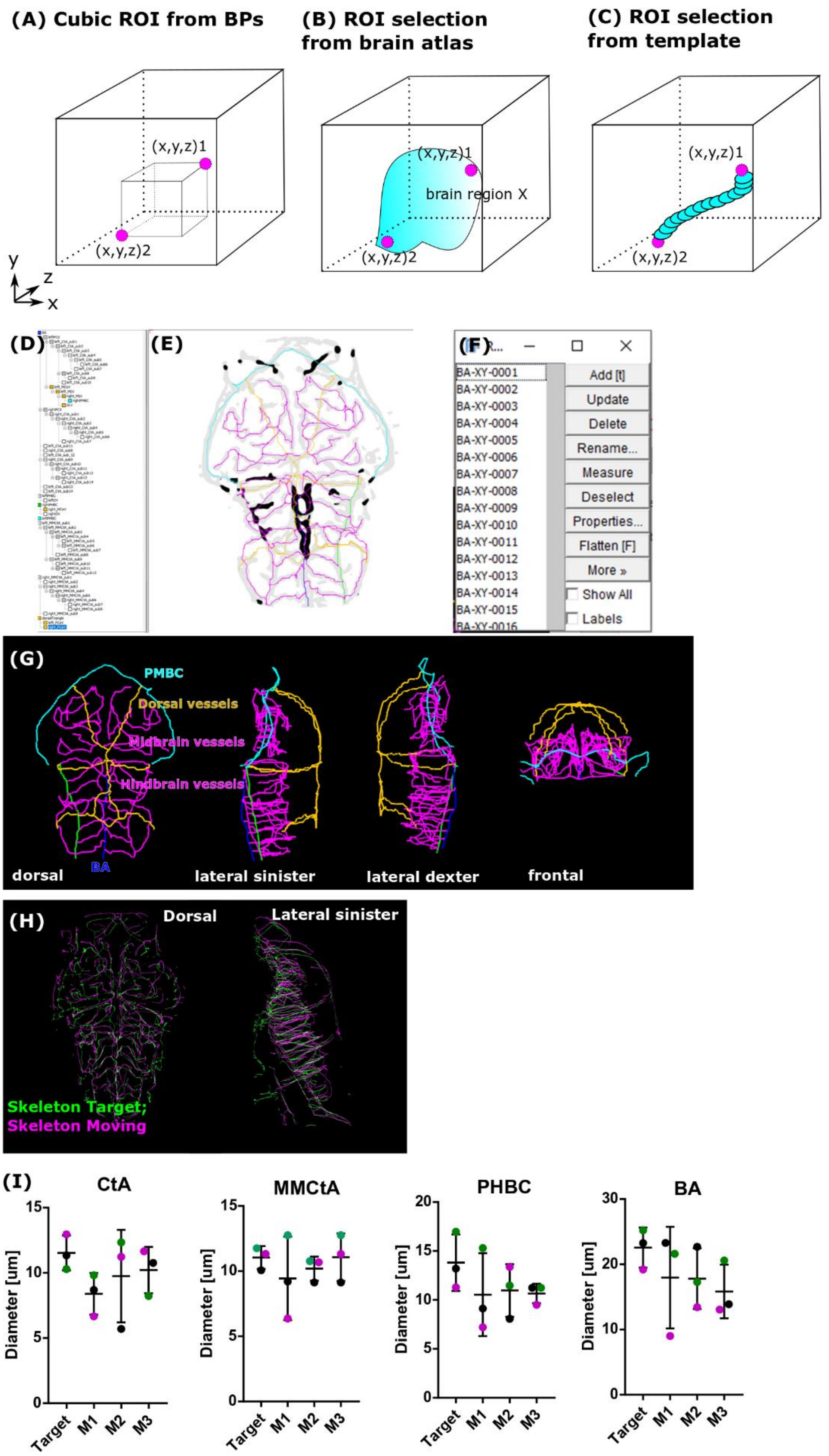

Development • Supplementary information

Fig. S17. Proof of principle examination of individual vessels.

A Vessel-specific cubic ROIs could be determined using vessel start-points $(x,y,z)_1$ and end-points $(x,y,z)_2$.

B Vessel-specific ROIs could be performed using brain regions, derived from and atlas.

C Vessel-specific ROIs could be extracted from a template and applied to registered fish.

D-F Semi-automatic vessel tracing allows annotation of individual vessels and ROI extraction.

G 3D rendering of semi-automatically traced cerebral vessels at 3dpf.

H Overlapped skeleton at 3dpf (following segmentation, registration, and skeletonization).

I Quantification of vessel diameters in four different fish (Target and 3 moving images; black - manual measurement in original image, green - manual measurement in segmented image, magenta - automatic measurement ; mean ± s.d.).

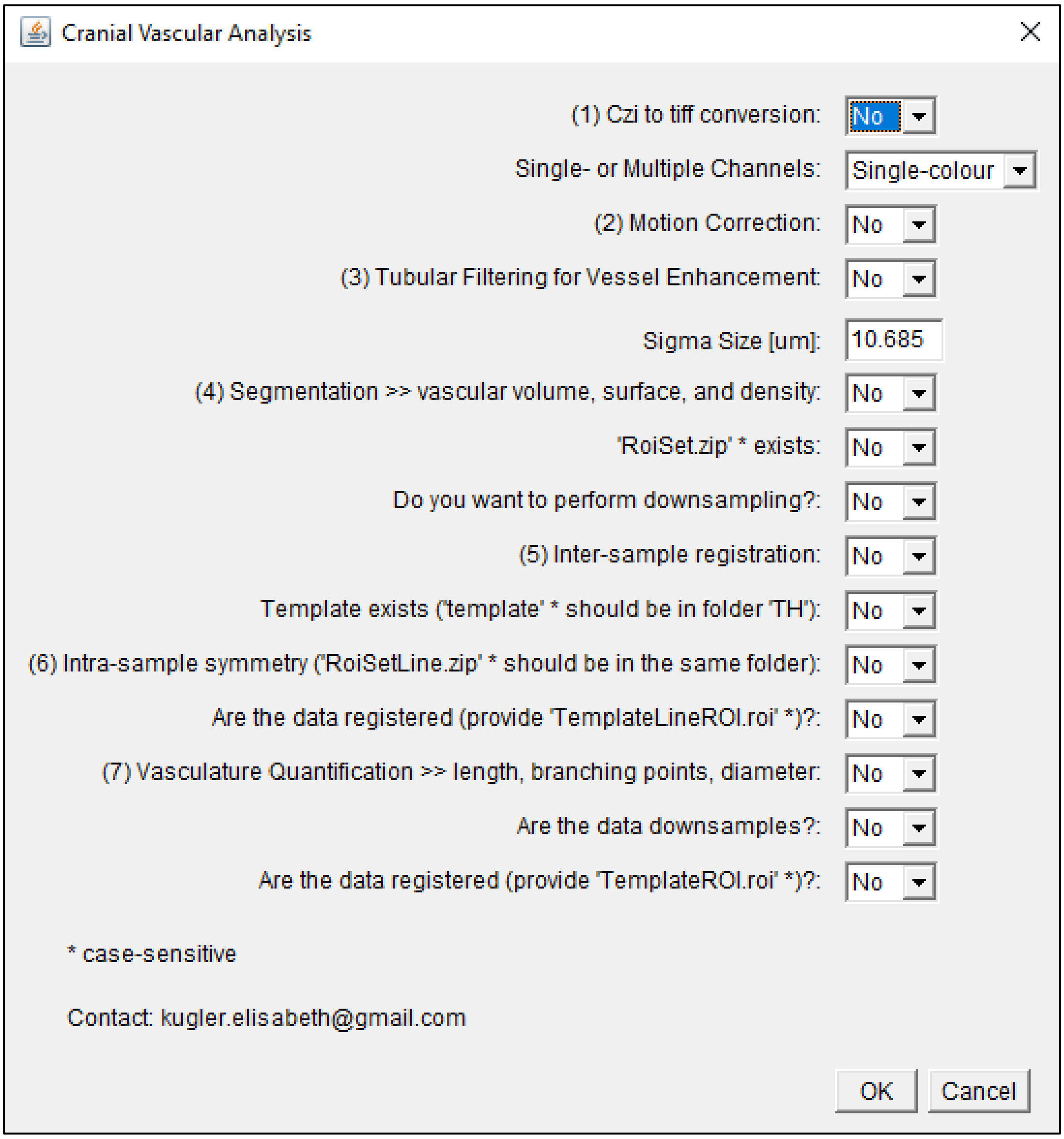

Fig. S18. A graphical user interface (GUI) was implemented to allow dissemination and applicability of the developed workflow.

Development • Supplementary information

Table S1. Mean parameter difference, following vascular parameter quantification. Quantification of similarity, volume, surface voxels, density, branching points, network length, network radius, and network complexity was conducted following morpholino-based knock-down of *tnnt2a, jagged-1a, jagged-1b, dll4, notch1b*, and *ccbe1* and application of chemicals for VEGF inhibition, Notch inhibition, F-actin polymerization inhibition, Myosin II inhibition, osmotic pressure (OP) increase, and membrane rigidity (MR) decrease.

Percentages represent difference of mean value between control morpholino (MO) and MO, or controls and treatment groups. Bold indicates statistically significant difference.

Abbreviations: act. – activation, inh. – inhibition, MR – membrane rigidity, OP – osmotic pressure;

	Similarity (S_n)	Volume (V_n)	Surface voxels (A_n)	Density (ρ_n)	Branching Points (BP_n)	Length (L_n)	Radius (R_n)	Complexity (C_n)
**MORPHOLINO KNOCKDOWN**								
tnnt2a	**-57%**	**-39%**	**-40%**	-0%	**-52%**	**-37%**	**-17%**	**-40%**
jagged-1a	+4%	-3%	-6%	-5%	**-24%**	**-26%**	-4%	-7%
jagged-1b	-16%	**-12%**	**-13%**	-3%	-14%	**-19%**	-3%	-13%
dll4	**-38%**	**-14%**	**-14%**	**-14%**	**-15%**	**-25%**	+4%	-14%
notch1b	-13%	+3%	+6%	-10%	-3%	-2%	-1%	+3%
ccbe1	+18%	**-19%**	**-21%**	+3%	**-27%**	**-24%**	-3%	-27%
**PHARMACOLOGICAL TREATMENTS**								
**VEGF inhibition**	-4%	**-10%**	**-9%**	-2%	**-15%**	**-13%**	**-3%**	-10%
**Notch inhibition**	-2%	**+9%**	**+9%**	-1%	+5%	+7%	+2%	+2%
**Actin polymerization inhibition**	**-40%**	**-25%**	**-17%**	**-15%**	**-39%**	**-28%**	**-12%**	-24%
**Myosin II inhibition**	-8%	**-25%**	**-17%**	+3%	**-26%**	**-17%**	**-14%**	-12%
**Osmotic pressure increase**	-4%	-4%	-4%	+1%	-2%	+3%	-1%	+1%
**Membrane rigidity decrease**	-2%	-1%	-1%	+1%	-3%	-3%	-1%	+4%

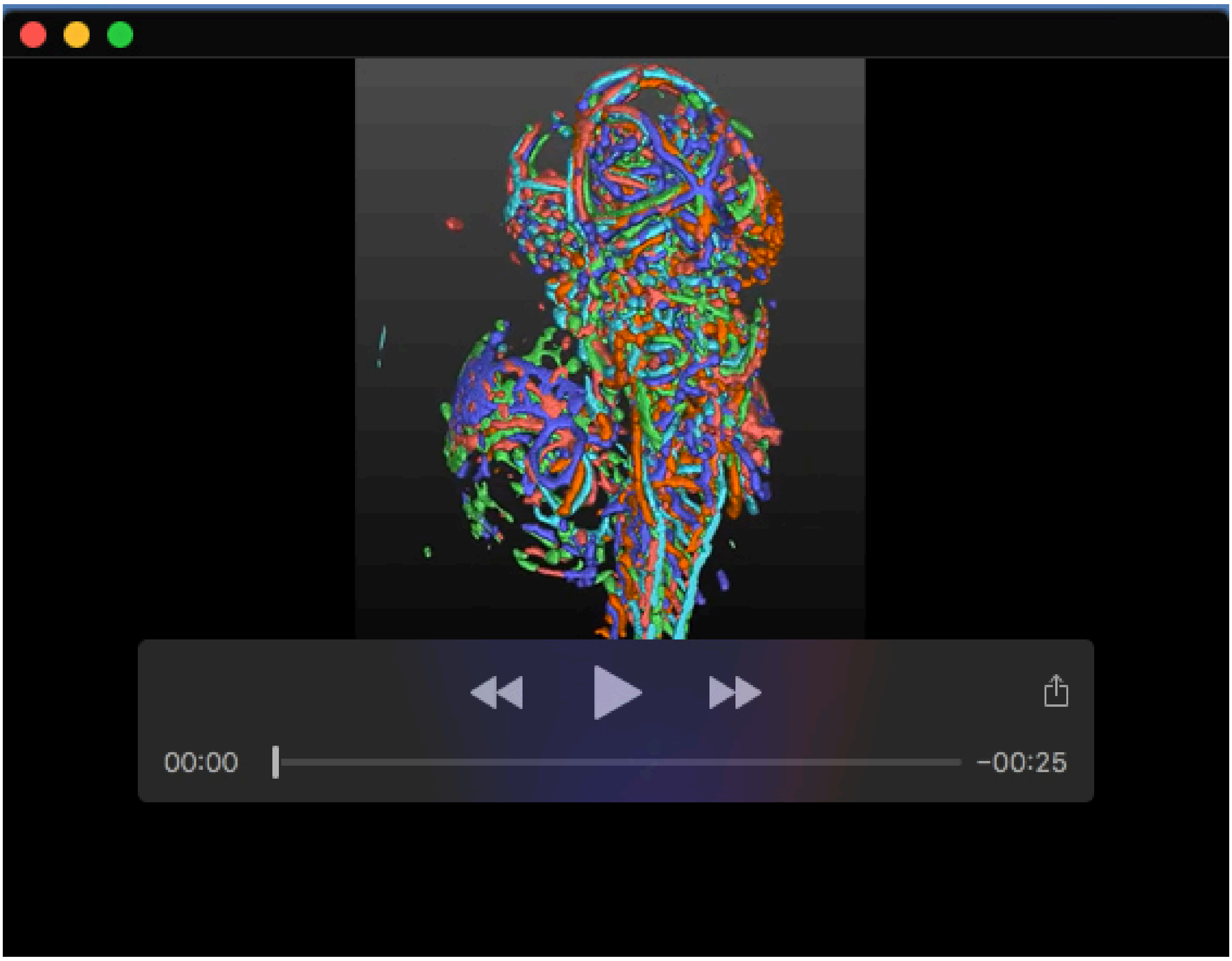

Movie 1. 2dpf inter-sample registration.

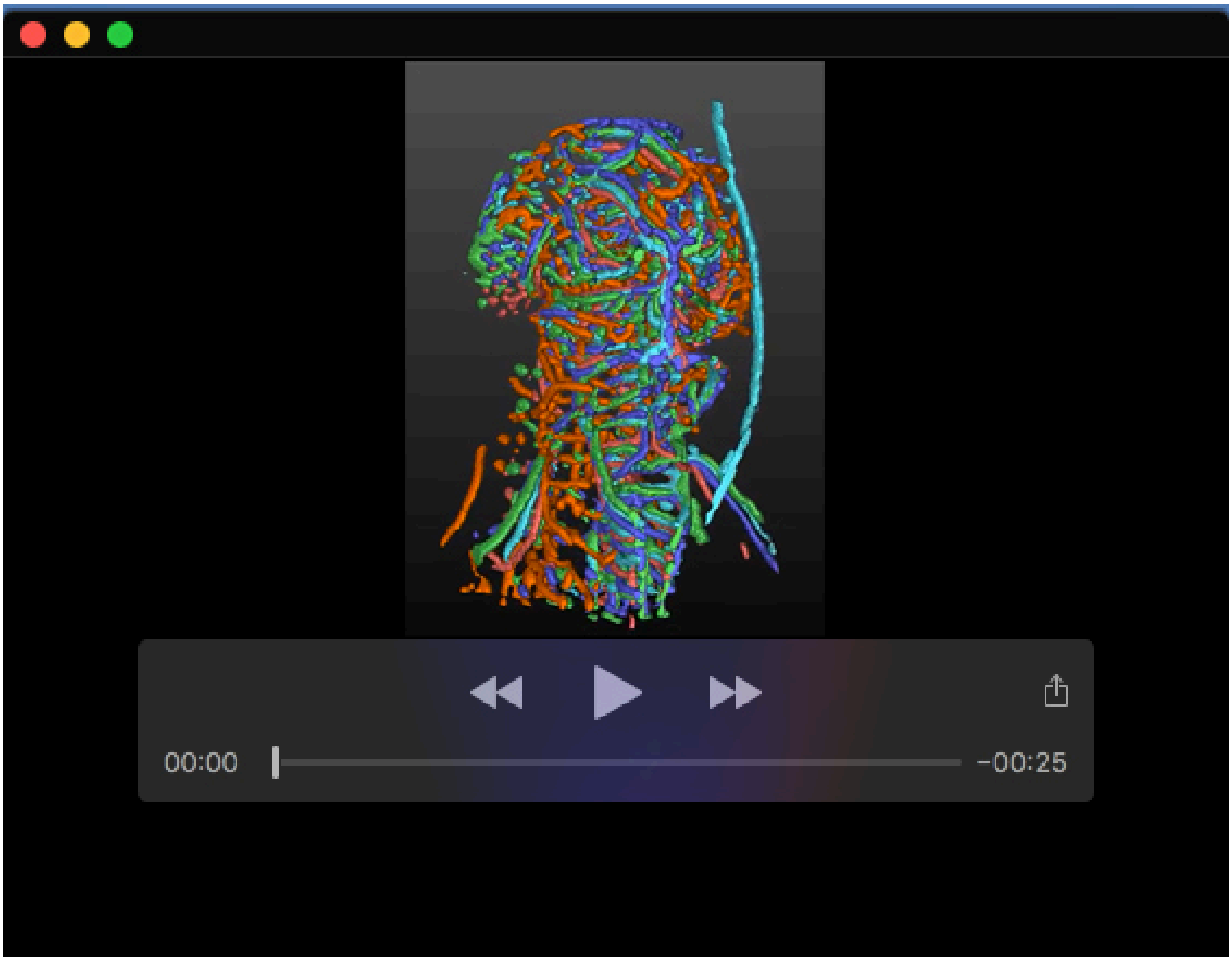

Movie 2. 3dpf inter-sample registration.

Development • Supplementary information

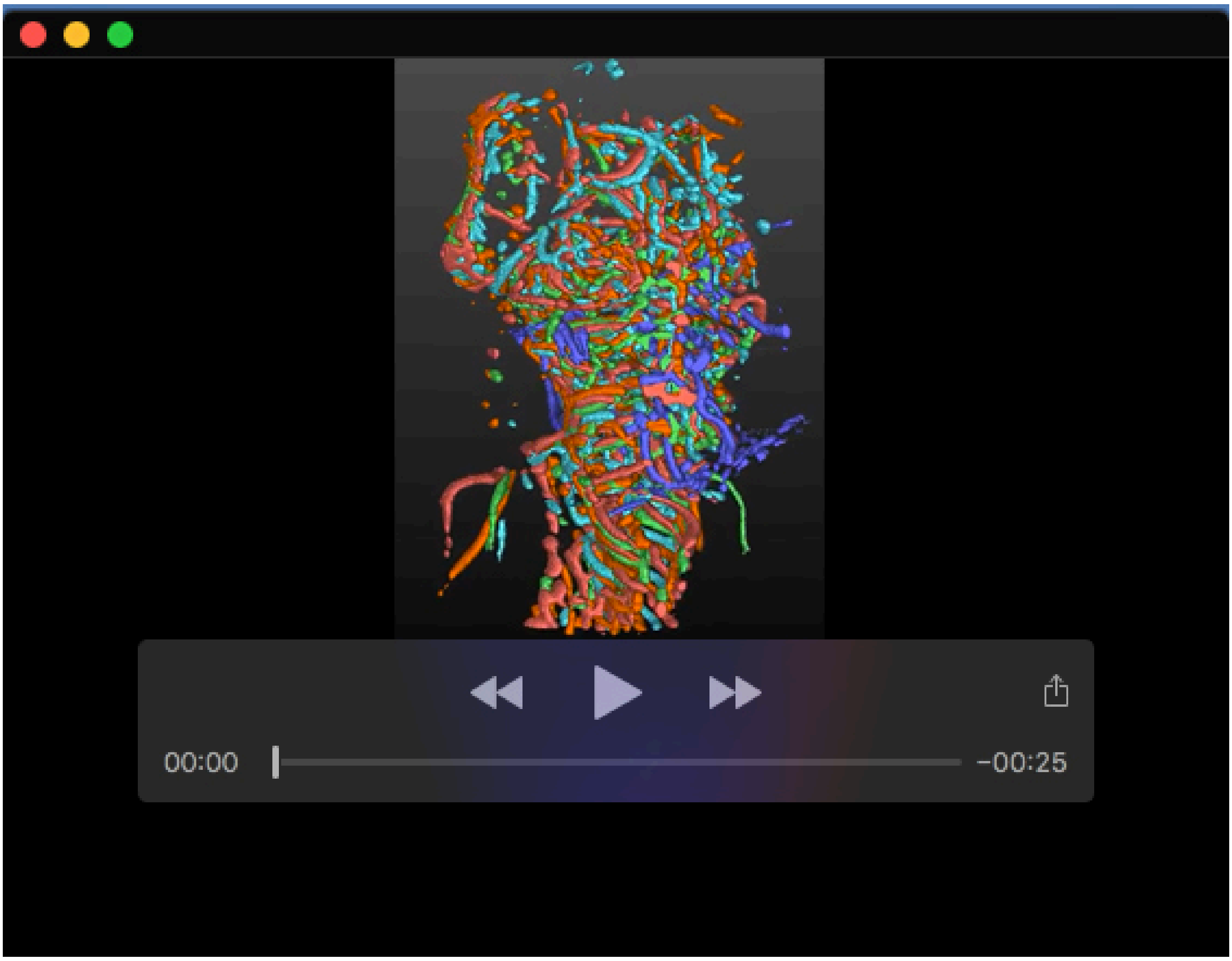

Movie 3. 4dpf inter-sample registration.

Development • Supplementary information

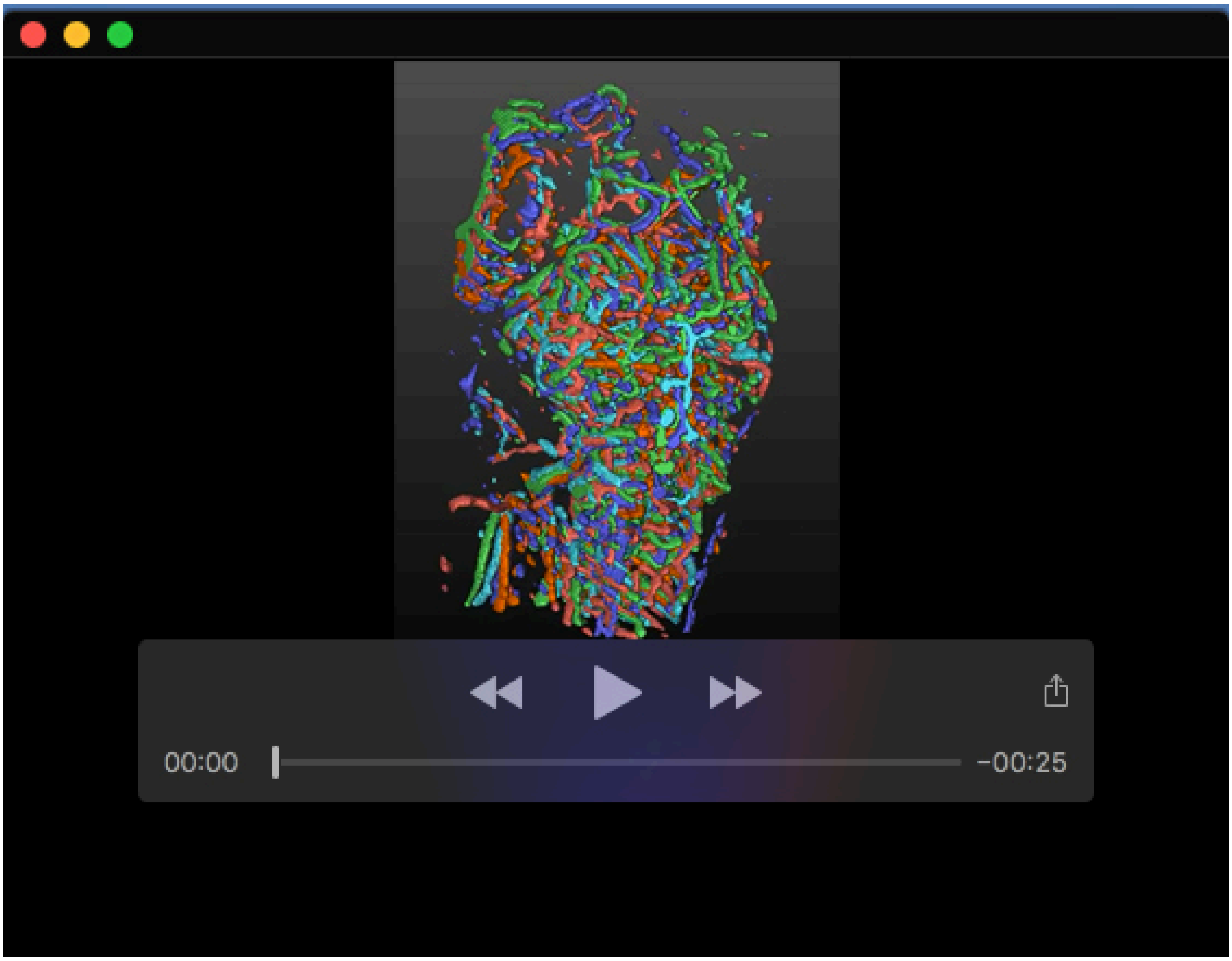

Movie 4. 5dpf inter-sample registration.

Development • Supplementary information

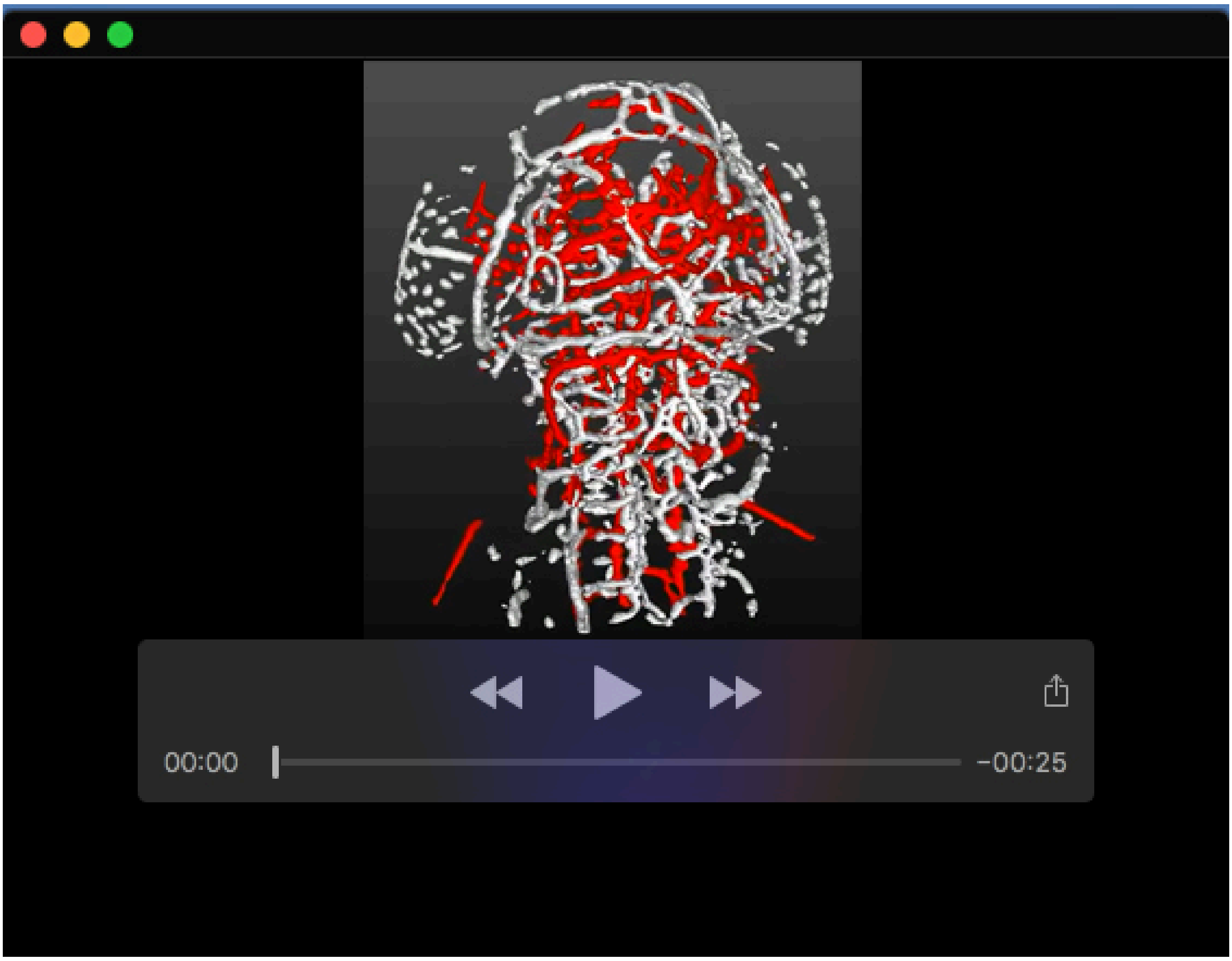

Movie 5. Inter-sample registration after *tnnt2a* knock-down.
Structural similarity was statistically significantly reduced in *tnnt2a* MO (red) in comparison to control MO (white).

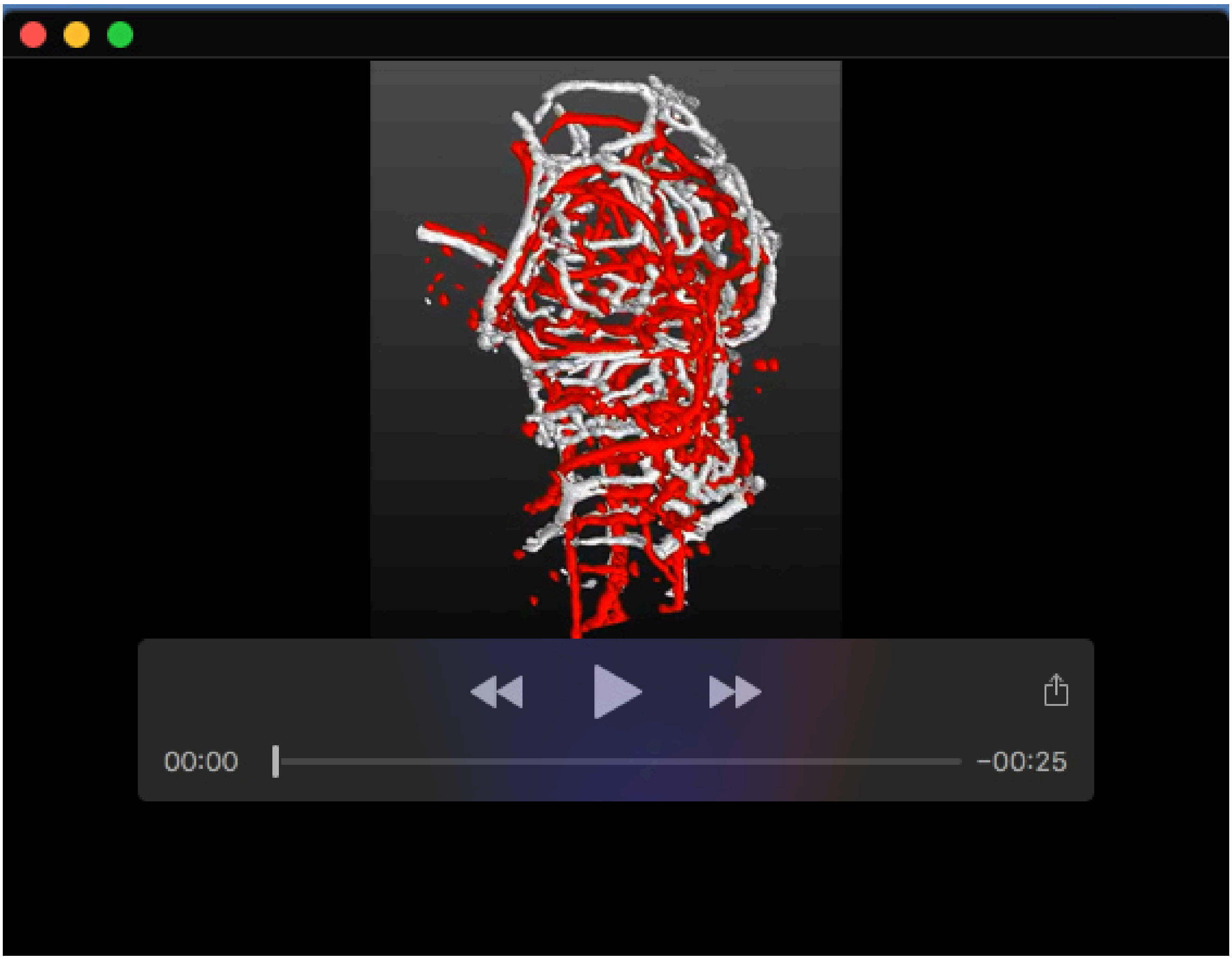

Movie 6. Inter-sample registration after *jagged-1a* knock-down.
Structural similarity was not statistically significantly altered in *jagged-1a* MO (red) in comparison to control MO (white).

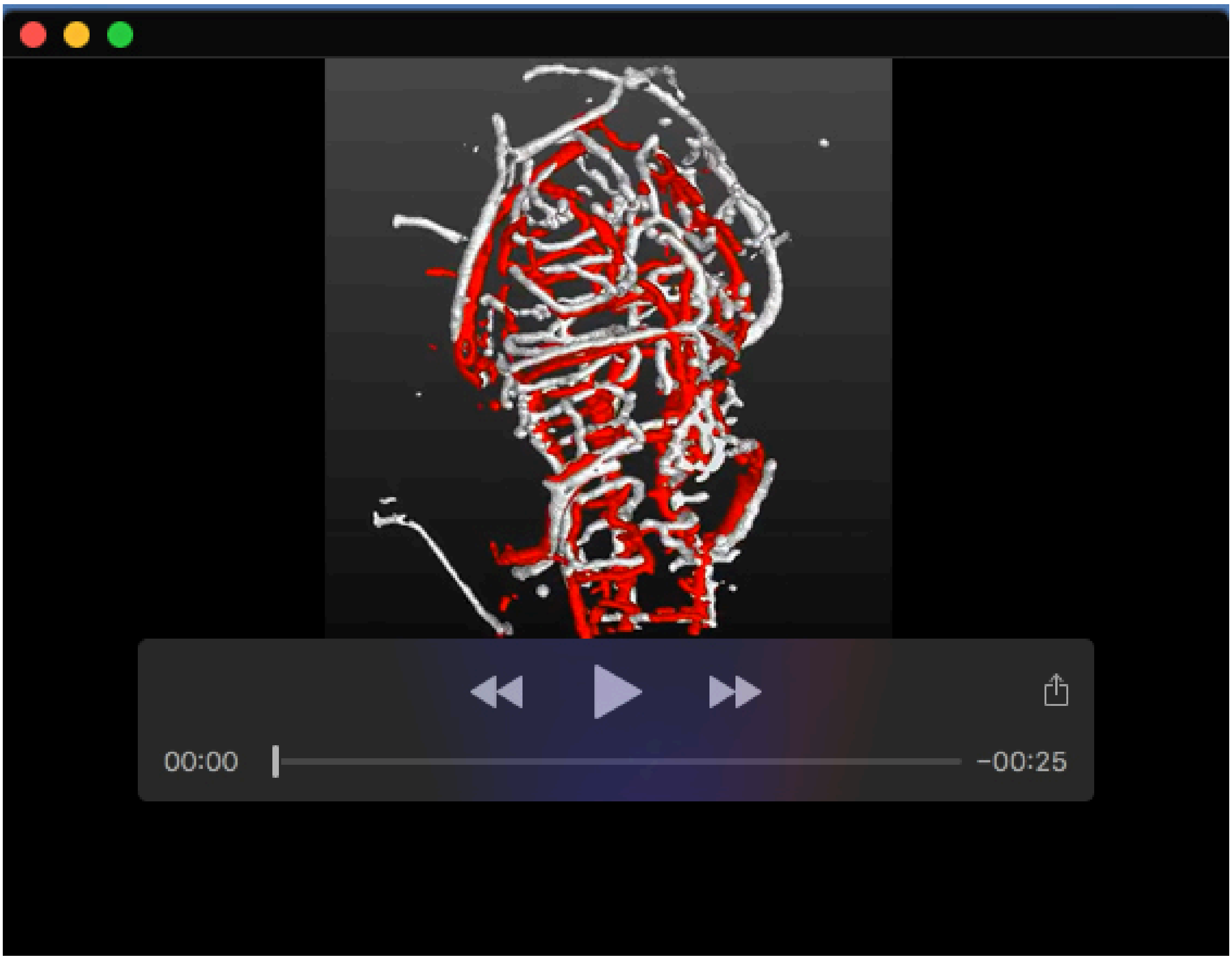

Movie 7. Inter-sample registration after *jagged-1b* knock-down.
Structural similarity was not statistically significantly altered in *jagged-1b* MO (red) in comparison to control MO (white).

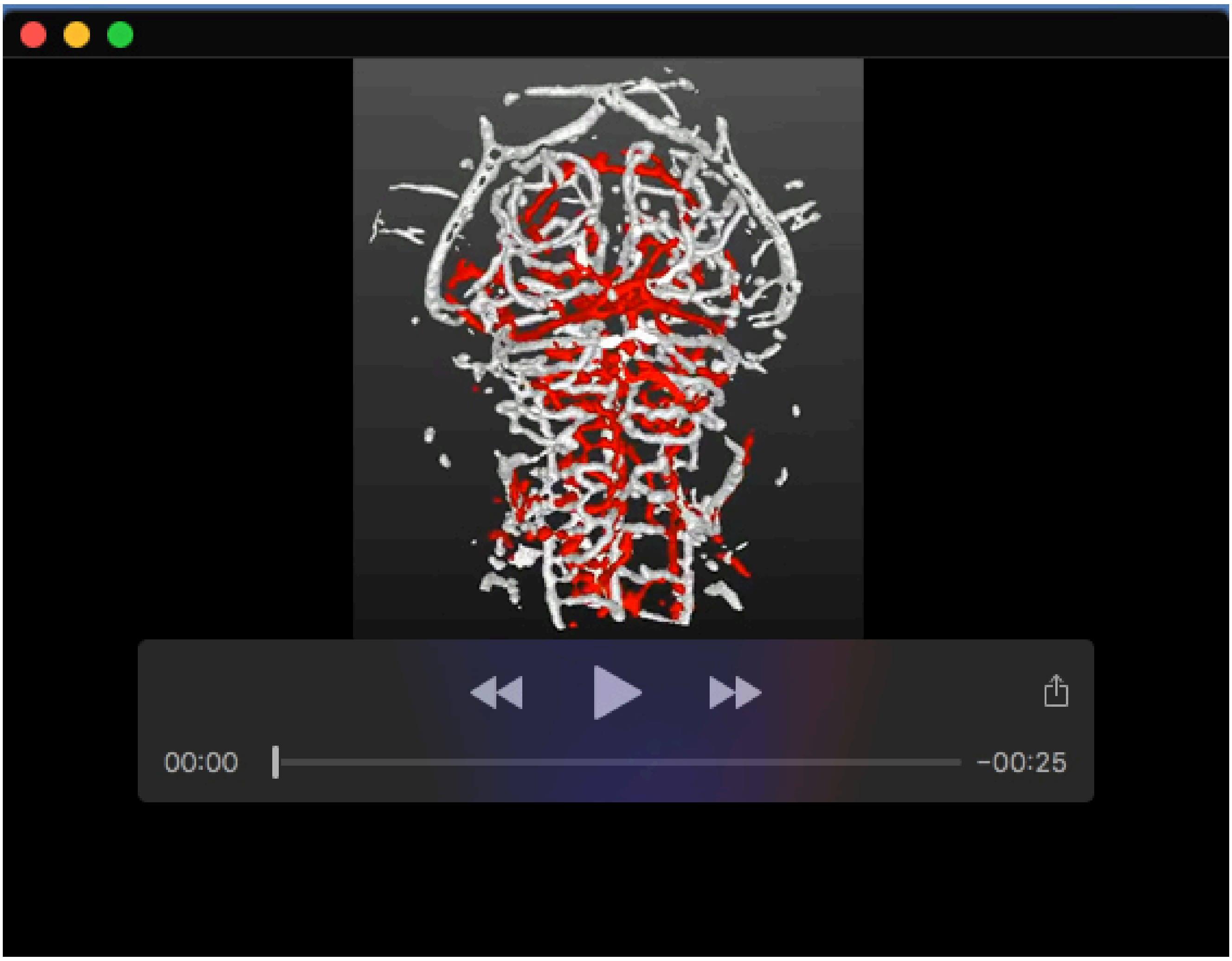

Movie 8. Inter-sample registration after *dll4* knock-down.
Structural similarity was statistically significantly reduced in *dll4* MO (red) in comparison to control MO (white).

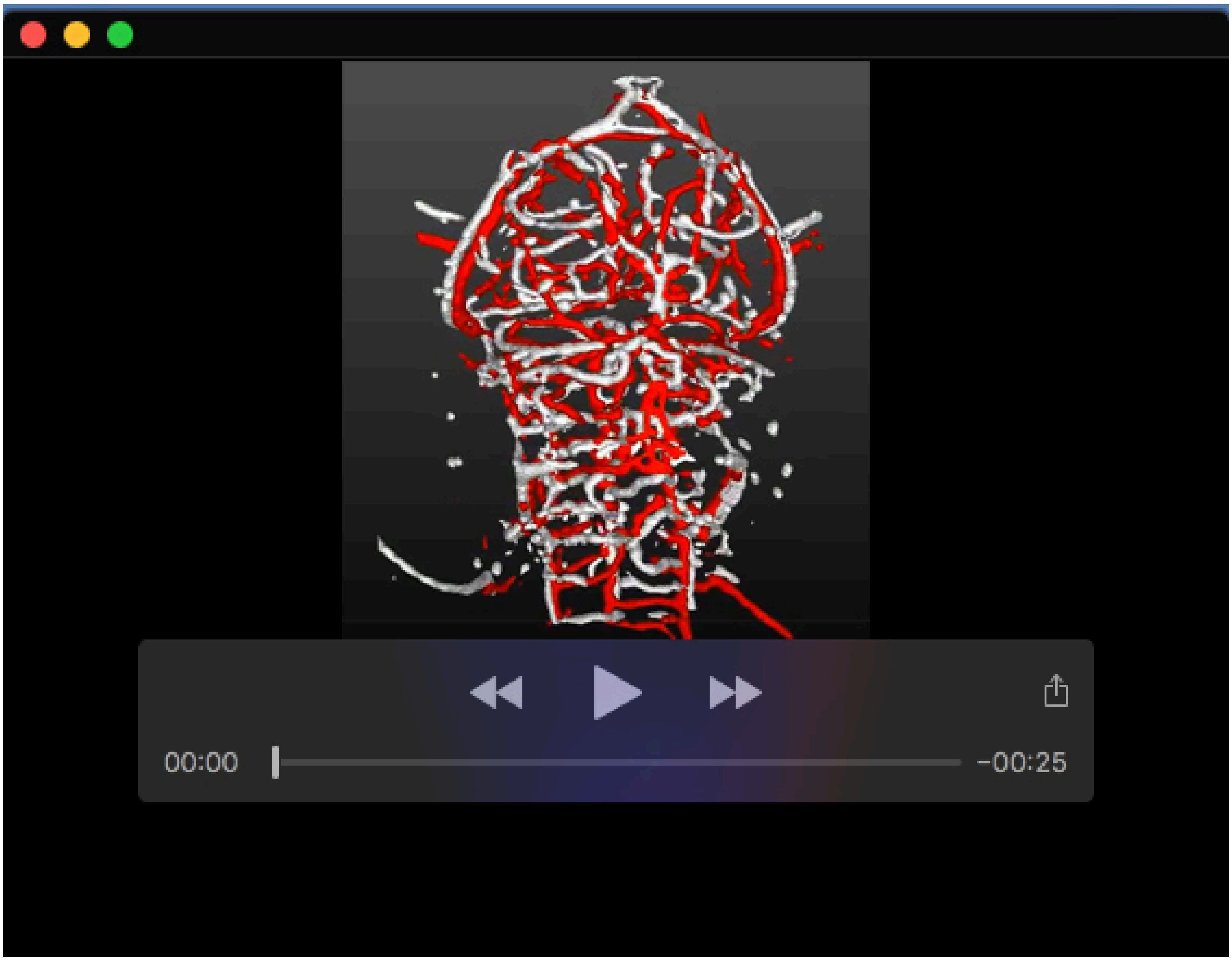

Movie 9. Inter-sample registration after *notch1b* knock-down.
Structural similarity was not statistically significantly altered in *notch1b* MO (red) in comparison to control MO (white).

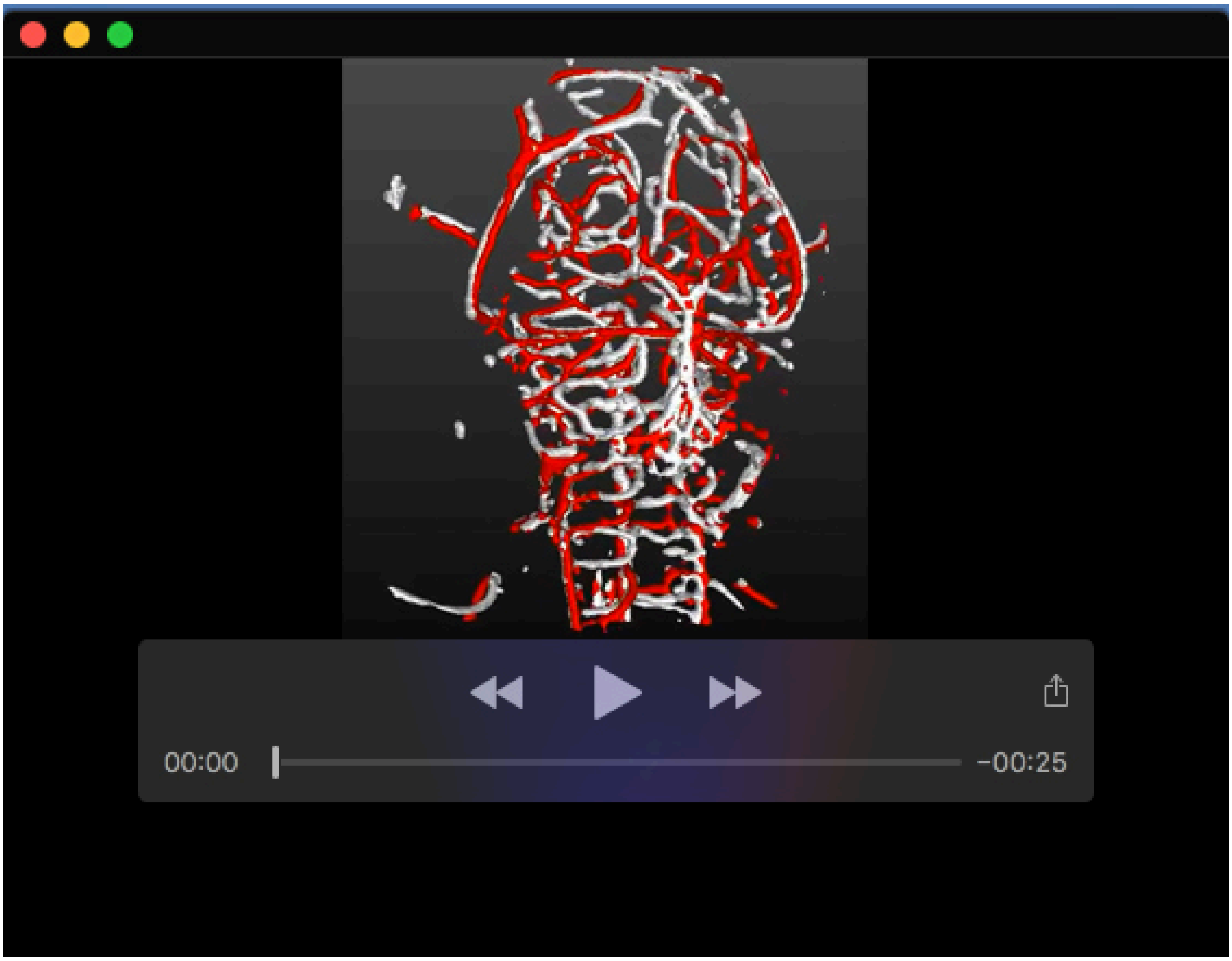

Movie 10. Inter-sample registration after *ccbe1* knock-down.
Structural similarity was not statistically significantly altered in *ccbe1* MO (red) in comparison to control MO (white).

Development • Supplementary information

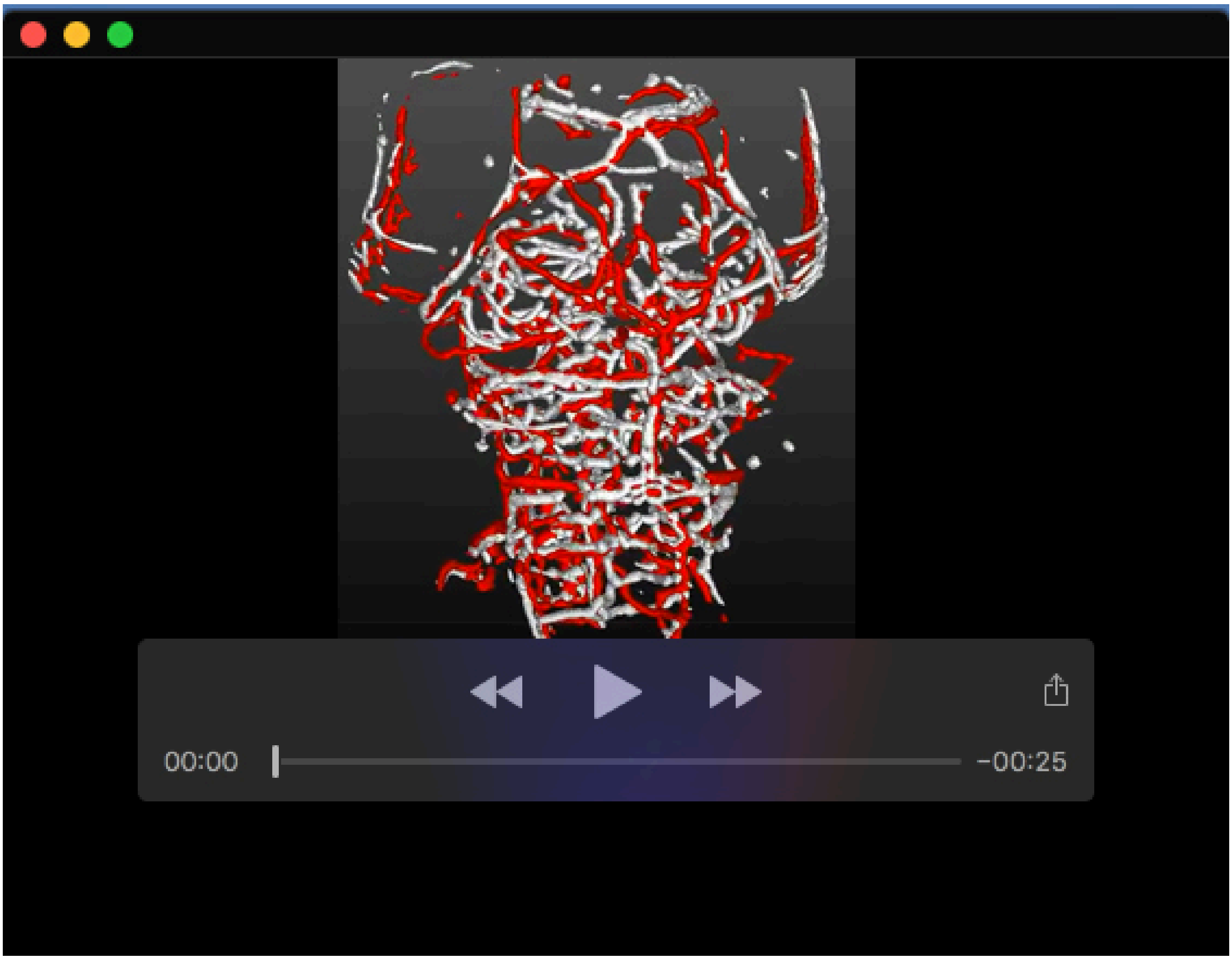

Movie 11. Inter-sample registration after VEGF inhibition.

Structural similarity was not statistically significantly altered upon AV951 treatment (red) in comparison to controls (white).

Development • Supplementary information

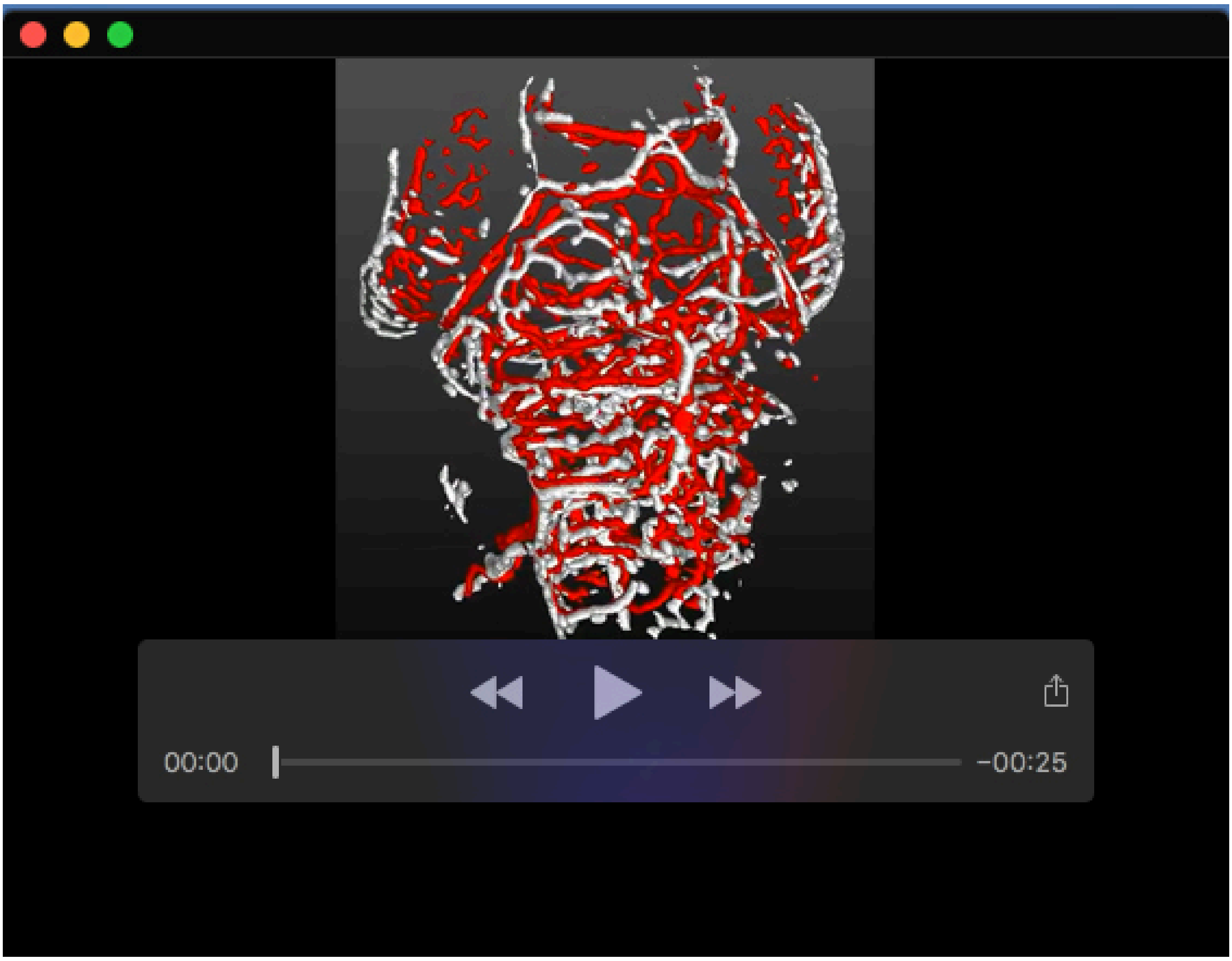

Movie 12. Inter-sample registration after Notch inhibition.

Structural similarity was not statistically significantly altered upon DAPT treatment (red) in comparison to controls (white).

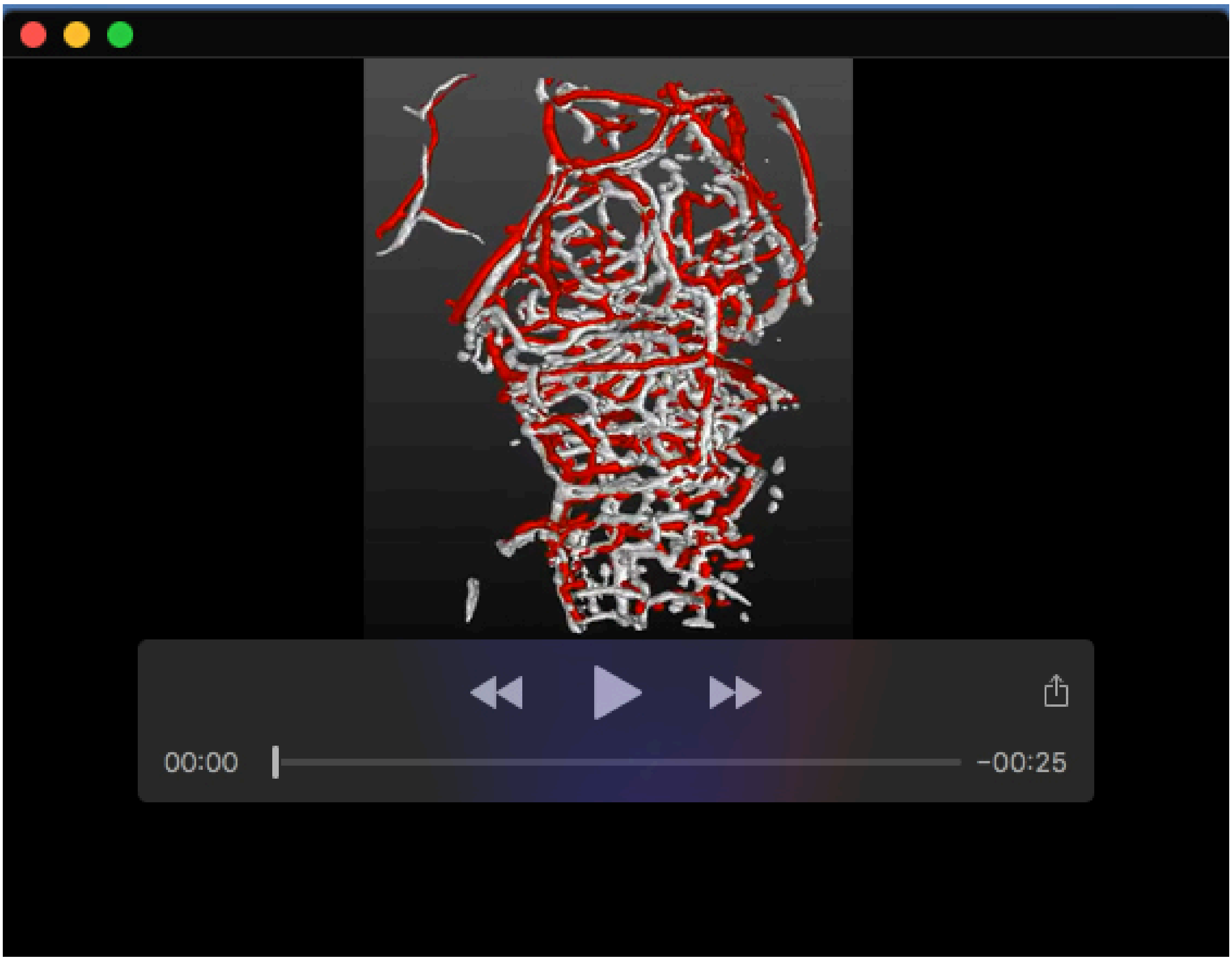

Movie 13. Inter-sample registration after actin polymerization inhibition. Structural similarity was statistically reduced upon Latrunculin B treatment (red) in comparison to controls (white).

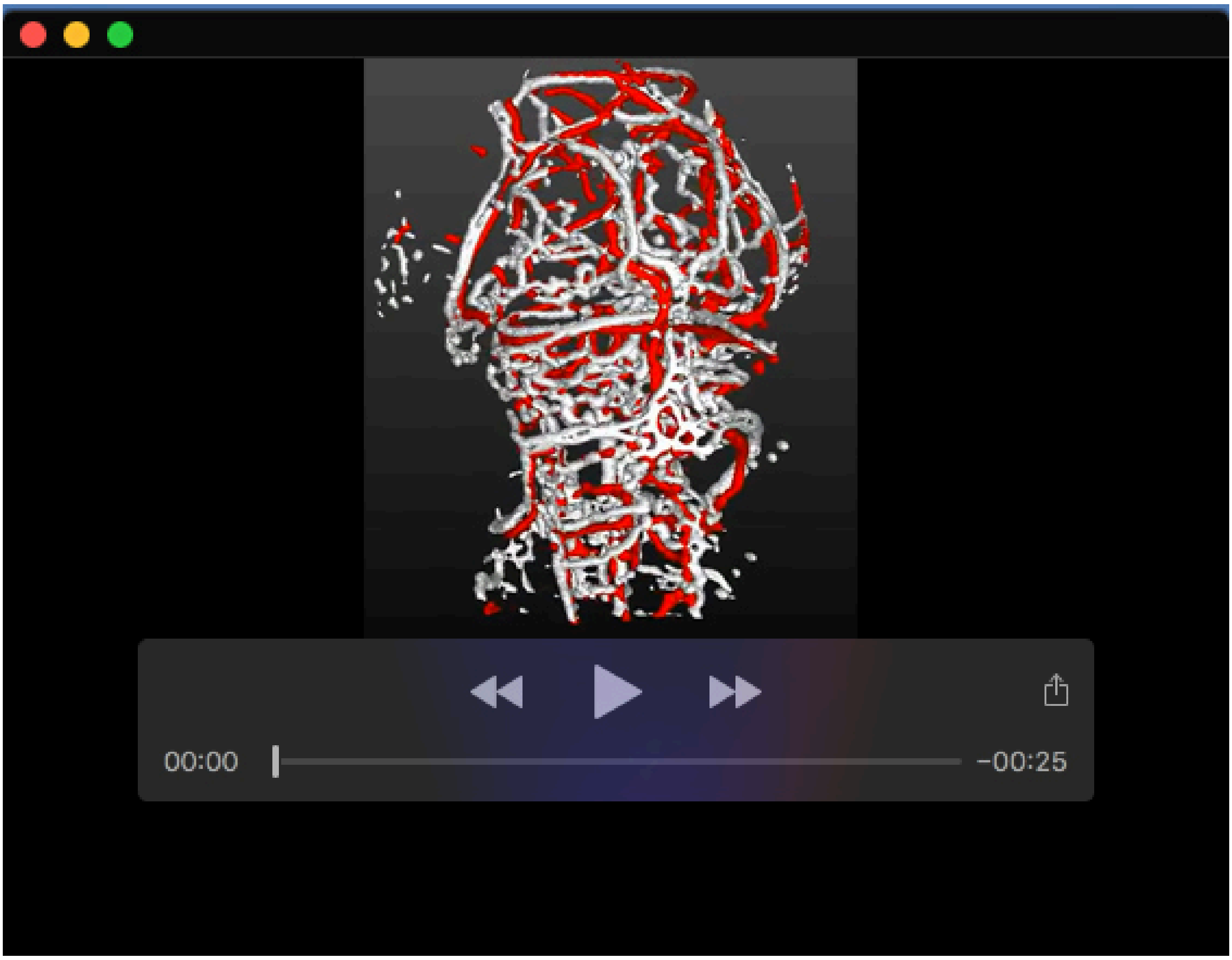

Movie 14. Inter-sample registration after Myosin II inhibition.

Structural similarity was not statistically significantly altered upon Blebbistatin treatment (red) in comparison to controls (white).

Development • Supplementary information

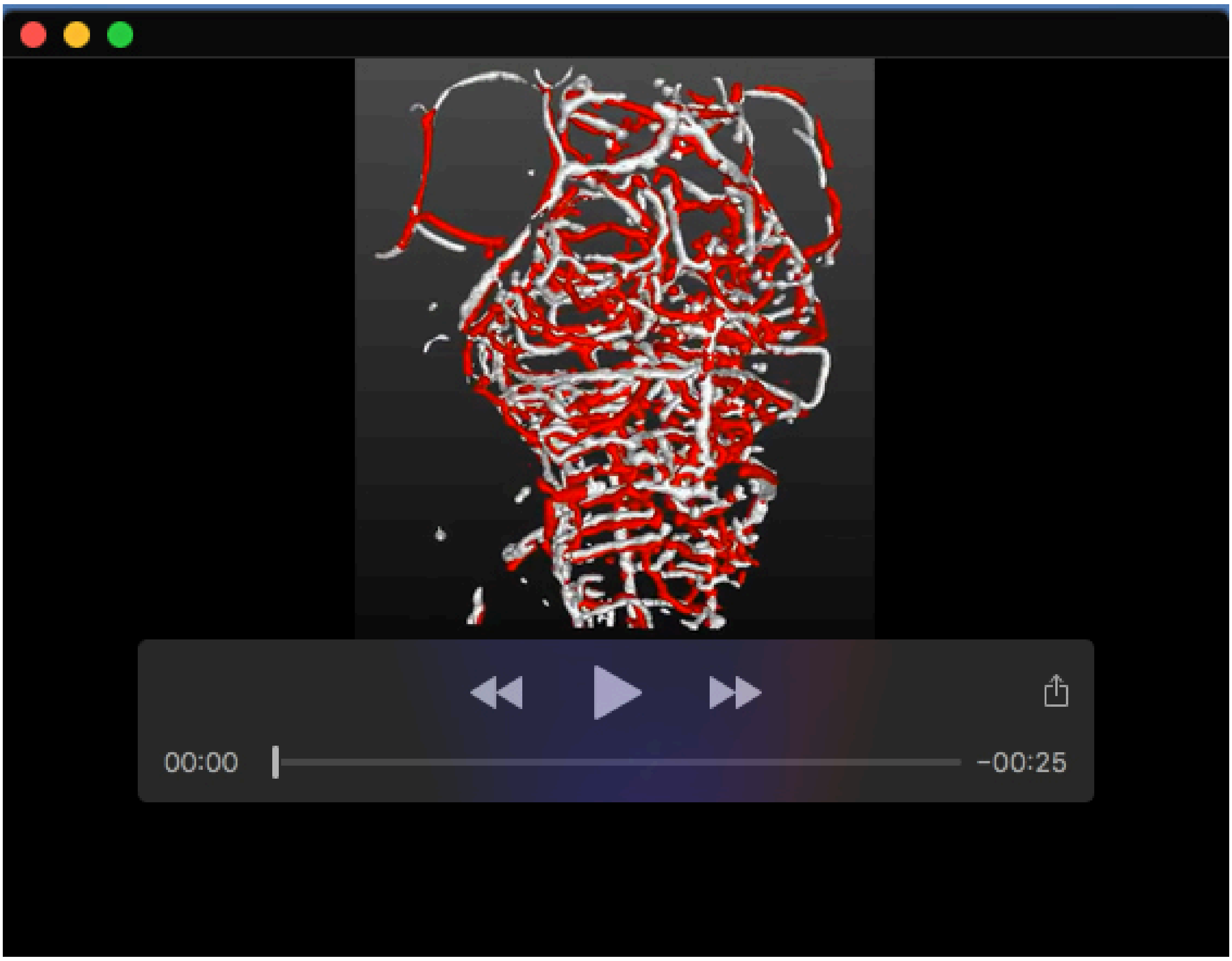

Movie 15. Inter-sample registration after glucose increase.

Structural similarity was not statistically significantly altered upon glucose treatment (red) in comparison to controls (white).

Development • Supplementary information

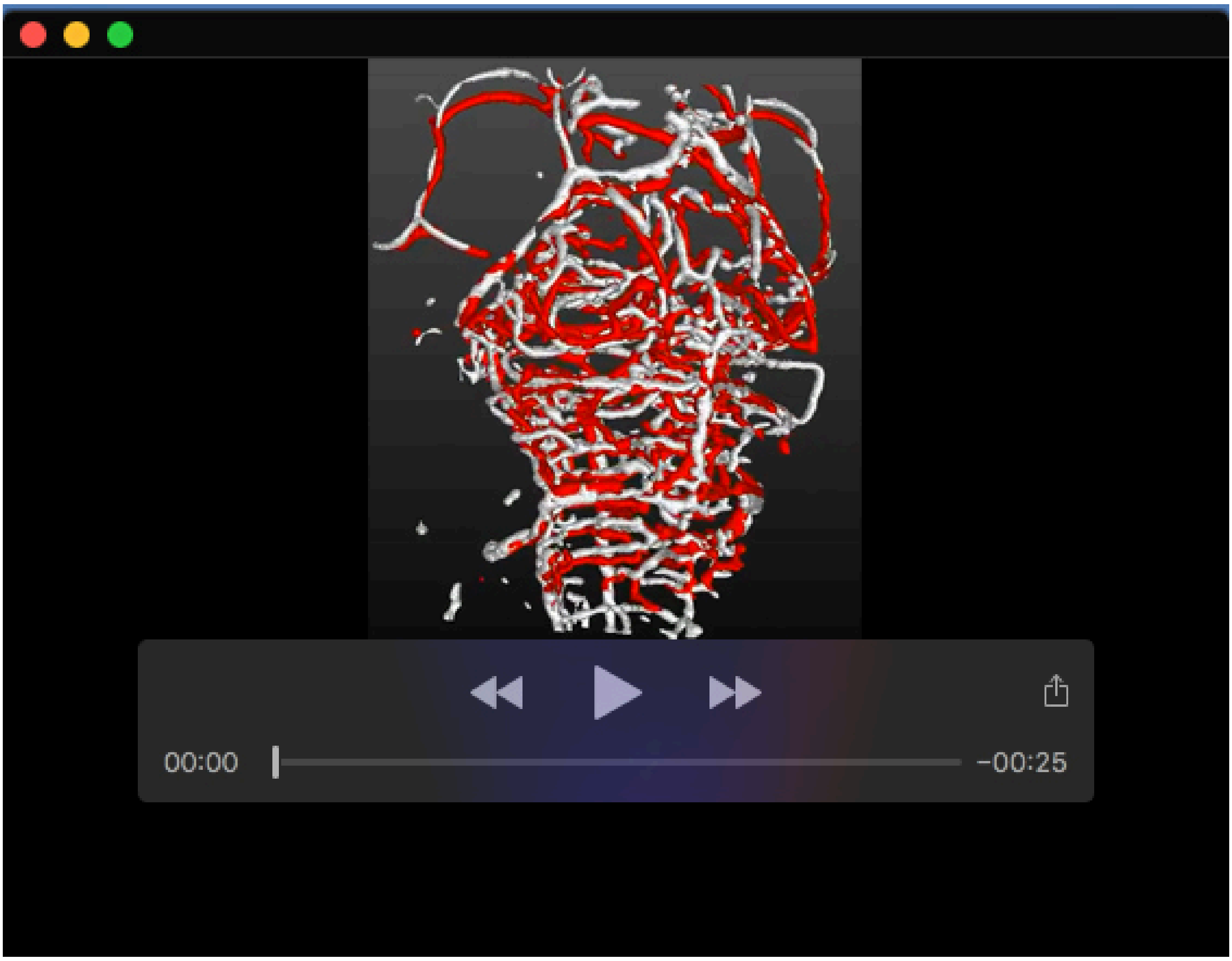

Movie 16. Inter-sample registration after DMSO treatment.

Structural similarity was not statistically significantly altered upon DMSO treatment (red) in comparison to controls (white).

Development • Supplementary information

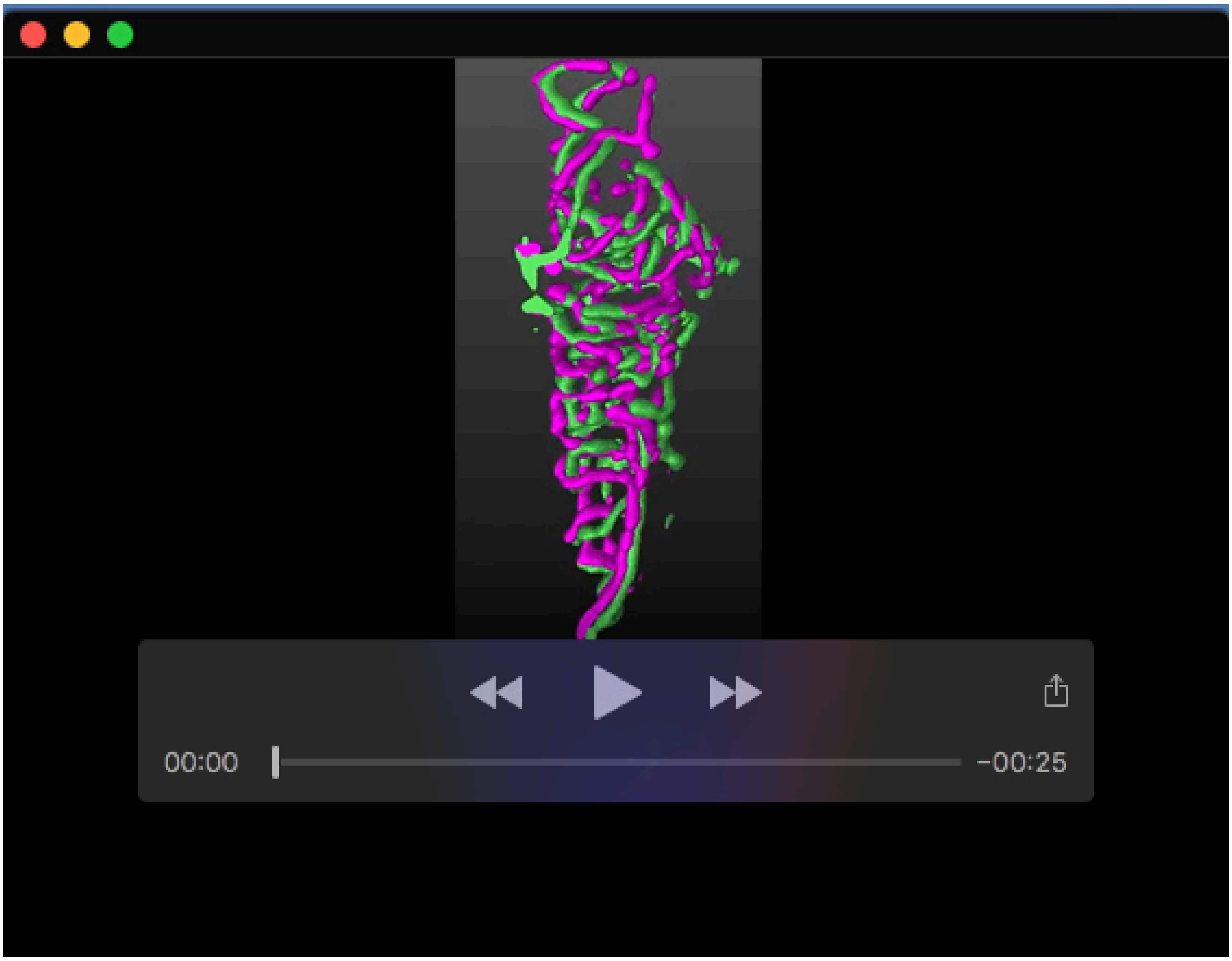

Movie 17. 3dpf intra-sample left-right symmetry. 3D rendering of overlapped left (green) and right (magenta) vasculature at 3dpf.
