# Supplementary material for: Zebrafish vascular quantification: a tool for quantification of three-dimensional zebrafish cerebrovascular architecture by automated image analysis
Source: Development. 2022 Feb 14;149(3):dev199720. doi: 10.1242/dev.199720 (PMC8918806; doi:10.1242/dev.199720)
Supplement: Reviewer comments [file develop-149-199720_review_history.pdf]

Development

# Zebrafish vascular quantification: a tool for quantification of three-dimensional zebrafish cerebrovascular architecture by automated image analysis

Elisabeth C. Kugler, James Frost, Vishmi Silva, Karen Plant, Karishma Chhabria, Tim J. A. Chico and Paul A. Armitage
DOI: 10.1242/dev.199720

**Editor**: Steve Wilson

**Review timeline**

| | |
|---|---|
| Original submission: | 5 September 2020 |
| Editorial decision: | 15 October 2020 |
| Resubmission: | 21 April 2021 |
| Editorial decision: | 1 July 2021 |
| First revision received: | 29 November 2021 |
| Accepted: | 17 December 2021 |


---

**Original submission**

<u>Decision letter</u>

MS ID#: DEVELOP/2020/196691
MS TITLE: 3D quantification of zebrafish cerebrovascular architecture by automated image analysis of light sheet fluorescence microscopy datasets

AUTHORS: Elisabeth Kugler, James Frost, Vishmi Silva, Karen Plant, Karishma Chhabria, Timothy Chico, and Paul Armitage
ARTICLE TYPE: Research Article

Dear Elisabeth,

I have now received all the referees reports on the above manuscript, and have reached a decision. The referees' comments are appended below, or you can access them online: please go to BenchPress and click on the 'Manuscripts with Decisions' queue in the Author Area.

As you will see from their reports, the referees recognise the potential of your work, but they also raise significant concerns about it. All referees have concerns about aspects of the image analysis pipeline and given that this is a methods paper, this means that I am afraid I have little choice other than to reject the paper at this stage.

However, having evaluated the paper, I do recognise the potential importance of this work. I would therefore be prepared to consider as a new submission an extension of this study that contains new data, methods, validation and discussions and that address fully the major concerns of the referees. It is essential to address all issues relating to the methods used and to validate all analyses. In addition, the use of morpholinos is potentially a concern - our expectation is that any new phenotype described using a morpholino should be fully validated using another approach (such as analysis of the

© 2022. Published by The Company of Biologists under the terms of the Creative Commons Attribution License (https://creativecommons.org/licenses/by/4.0/).

consequences of a genetic mutation). I appreciate that you may, in part, be using morpholinos to generate data simply to test the analysis pipeline rather than to draw new conclusions about role of the targeted gene but even if so, it would be advisable to use genes for which phenotypes are already known. The work required on your manuscript goes beyond a standard revision of the paper. Please bear in mind that the referees will assess the novelty of your work in the context of all previous publications, including those published between now and the time of resubmission.

Reviewer 1 Advance Summary and Potential Significance to Field:
In this study, Kugler et. al. develop a new computational pipeline to enable rigorous 3D quantitative image analysis of cerebrovascular architecture during zebrafish development. First, using images of the zebrafish cerebral vasculature acquired via light sheet microscopy, Kugler and colleagues apply image enhancement, segmentation and manual or automatic inter-sample registration to align embryo images and define areas of high similarity. Next, the authors establish a quantification workflow for automated acquisition of vessel volume, surface area, density, length, branch points, radius and complexity. Then they apply this workflow to quantify architectural changes occurring (1) during normal cerebrovascular development, (2) upon loss of blood flow, (3) following perturbation of vascular gene expression / function, and (4) in distinct regions of the brain. Moreover, they have made this pipeline publicly available as an online tool available via open-source Fiji software. As such, Kugler et. al. has generated a useful resource that may be adopted by many in the vascular biology community.

Although the function of this tool is not in doubt, what is less clear is the significance of the insights that can be generated using this workflow. Most importantly, the quantified phenotypes described in this study are somewhat superficial and lack precise details of the brain regions involved, specific vessels affected, persistence of the observed changes or underlying changes in cell behavior responsible for these events. Without further investigation of the sensitivity of this tool and rigorous confirmation of its ability to pinpoint region-specific subtle phenotypes that would normally be missed, the broad utility of this pipeline and level of insight it can generate over pre-existing methods remains unclear.

Reviewer 1 Comments for the Author:
Whilst I am overall positive about this manuscript, there are several points that I consider to be sufficiently important that they should be addressed with revisions prior to acceptance for publication:

(1) Kugler and colleagues begin the study by stating that the lack of robust automated approaches to quantify vascular anatomy in 3D prevents detection of subtle phenotypes. However, a question still remains as to the sensitivity of their analytical workflow and whether it can truly detect subtle changes. Several embryo manipulations appear to elicit little to no effect on cerebrovascular architecture using their pipeline (e.g. notch 1b knock-down, LNAME, Wnt inhibition, Wnt activation, osmotic pressure, 0.25% DMSO, left-right patterning). This is somewhat surprising and, in some cases, inconsistent with the literature (e.g. Wnt). As such, it is possible that this tool only detects severe changes in cerebrovascular architecture, which may be easy to detect by conventional manual images analyses. Indeed, many of the manipulations used to validate the workflow induce severe vascular defects that are easy to detect (e.g. lack of blood flow, VEGF inhibition, Notch inhibition, actin/ myosin inhibition, etc). The authors need to provide additional evidence that this tool is sufficiently sensitive to detect subtle phenotypes and outperforms other forms of image analysis in doing this. In particular, use of several positive control manipulations that are already known to induce subtle cerebrovascular phenotypes (e.g. loss of cxcl12b, loss of cxcr4a,

© 2022. Published by The Company of Biologists under the terms of the Creative Commons Attribution License (https://creativecommons.org/licenses/by/4.0/).

inhibition of Wnt using IWR-1) should be analyzed to confirm that their tool can robustly detect these. Otherwise, the broader utility of this tool and its improvement on other approaches are unclear.

(2) The output of the workflow is somewhat superficial and does not generate much insight into the cause of observed defects, other than to describe global shifts in the analyzed parameters. Evidence that the pipeline can be exploited to define specific underlying phenotypes (i.e. as a route to understanding gene function) is essential to confirm that this tool "will aid discovery of novel insights into vascular development". For example, can the workflow be modified to define the precise location of extra or lost branch points? Similarly, can it define specific zones of increased complexity, surface area, radius, density or volume? Can it determine where length been added to or lost from the Wt vasculature? If vessel similarity is decreased, which specific regions are losing similarity? Can the tool determine if all of the above defects are associated with certain vascular beds, specific vessels or even arterial verses venous tissues? Can the specific location of these defects then be mapped to patterns of gene expression, such as DLL4, CXCR4, VEGFRs? Addressing these questions will determine how informative the tool can be, otherwise, the insights generated by this workflow are not so compelling.

(3) At the start of page 5, Kugler et. al. state that image registration improves the reproducibility of quantification. However, there is not adequate investigation of this. A key novelty of the workflow is the use of this initial image inter-sample registration, and all the other image analysis methods used appear to be standard approaches that are routinely applied to non-registered samples. As such, the authors need to demonstrate how essential the image registration is to the subsequent workflow analysis. What happens to the variability of measurements for the other 7 parameters quantified in this study if images are not pre-registered? Does image registration truly improve the reproducibility of this quantification?

Minor comments:
(1) Kugler et. al. frequently refers to genetic manipulations. However, I did not identify any genetic manipulations in this study. Please re-word.

(2) In some figures, quantification of all 8 metrics is absent. (e.g. no surface area or density in Fig.3, which would be expected to increase) Why are some omitted? Please make reference to all measurements for consistency and to enable a full comparison between embryo manipulations.

Reviewer 2 Advance Summary and Potential Significance to Field:
This manuscript by Kugler et al. presents an analytical pipeline for 3D quantification of cerebral vasculature for larval zebrafish. The authors first describe registration approaches (landmark based and automatic rigid registration) of light-sheet microscopy data of cerebral vasculature to bring 3D images from different samples into a common co-ordinate system. Using these registered images, the authors then quantify various vasculature parameters such as similarity, volume, network length, etc. to describe how these properties change during development from 2 - 5 dpf. Next, applying a range of genetic and pharmacological manipulations that could impact cerebral vascular development, the authors employ their pipeline to characterize change in vasculature properties compared to controls. Finally, the authors also provide a comparison of vasculature parameters between mid/hindbrain and L-R sides of the brain. The authors have developed the pipeline with a Graphical User Interface in the widely used open source image processing software Fiji, and provided source code for their pipeline.

© 2022. Published by The Company of Biologists under the terms of the Creative Commons Attribution License (https://creativecommons.org/licenses/by/4.0/).

Broadly, I think the manuscript tries to address an essential gap in 3D analyses of vasculature parameters across the brain. This could potentially be valuable for the field, and not just for those working with zebrafish as a model system. However, due to the concerns I highlight below, my enthusiasm for publishing the manuscript in its current form is diminished. I have a few major conceptual concerns with the manuscript and some minor concerns, that the authors should address before publication.

Reviewer 2 Comments for the Author:

Major concerns:

(i) The authors employ rigid registration to bring different samples to the same co-ordinate system. However, as is evident from Fig. S2 (A), (B) and very low values for Dice coefficient (similarity scores) throughout the manuscript, the rigid registration performs poorly. CMTK plugins for Fiji (ImageJ) allow employing multi-step registration with both rigid and non-rigid (affine or other) approaches. The primary purpose of being able to register different samples to a common reference frame is to be able to spatially localize vascular feature changes, which does not come across in the manuscript. The authors should address this aspect, if possible, by including non-rigid, more accurate registration in their pipeline.
(ii) The Dice coefficient is used throughout the manuscript as a measure to interpret similarity within samples and between samples that underwent different genetic or pharmacological manipulations. However, as pointed out in (i), the variance and the values of Dice coefficient themselves could simply be a result of the registration quality. Therefore, using similarity between samples that have undergone different treatments to interpret mechanistic differences has little meaning. The authors should avoid using the Dice coefficient as a measure of changes in vascular properties.
(iii) While the authors correctly state that registered volumes in the same coordinate system can be used to compare vascular parameters between brain regions or left-right symmetry, it is not clear if the implementation of these anatomical masks is a part of the pipeline. Figure 1 and Figure 6 seem to suggest this. However, it becomes even more relevant considering the genetic or pharmacological manipulations included in this study, to include this analysis as these signalling pathways are known to impact control of symmetry and topology along the anterior-posterior or dorso-ventral or left-right axes during development. If this analysis is part of the pipeline, why was it not performed for all manipulations or for developmental snapshots, instead of presenting average vasculature parameters across the entire brain?
Surely, quantifying these parameters by brain region or by comparing symmetry for all manipulations, the authors could add a much needed novel component to their analysis pipeline and this study, thereby helping the community gain discovery-based mechanistic insights! It is critical that the authors address this aspect.

Minor concerns:
(i) Which datasets were specifically acquired for this manuscript and which are being re-used from previous studies by the authors? This does not come across clearly. This aspect should be made very clear both in the Results and Methods section.
(ii) Often, references to the Methods section or a brief explanation of the analysis is lacking in the Results section (for example: it is not clear what the output of the Sholl analysis is? Or how was the clustering in Figure 5 performed and validated? Or was it validated for the optimal number of clusters?). This should be rectified throughout the manuscript. This will help the authors reach a broader audience.
(iii) The hyperlink to the test dataset in the supplemental section is restricted. While it is very good that the authors provide not only the source code for their pipeline but

© 2022. Published by The Company of Biologists under the terms of the Creative Commons Attribution License (https://creativecommons.org/licenses/by/4.0/).

also provide example datasets, this should be made easily accessible to all readers on an open repository.
(iv) The color scales for 'similarity' for MIPs across all figures do not have values next to them. The values should be displayed, and all MIPs scaled to a common value range (i.e. for example, all MIPs should be between 0 - 0.3 on the color scale). This will make comparison across figures possible.
(v) It should also be made clear in the Methods section how and which template (or target) 3D volumes were chosen for registration across all treatments. For example, the tnnt2a morpholino injections result in very little structure being present in cerebral vasculature. Does this influence template selection and therefore registration accuracy?

Reviewer 3 Advance Summary and Potential Significance to Field:
Kugler et al present a workflow and software to analyze the zebrafish vasculature in LSFM data. The authors use the workflow to quantify the vasculature in wt and morphant zebrafish. The manuscript is well written and presents some valuable data. However, it is superficial and lacks the necessary controls and error estimation. The segmentation is only an approximation of the vasculature and cannot be compared without proper error estimation.

Reviewer 3 Comments for the Author:

Major comments:

1) This may be the "first easily applicable 3D image analysis pipeline for zebrafish vasculature" - but is it accurate? Before going into the various experiments, it is important to show that the vasculature is correctly segmented.
"Image segmentation is the process of assigning a label to every pixel in an image such that pixels with the same label share certain characteristics."(Wikipedia) This means in this case that a) every voxel that is labeled as vasculature is indeed vasculature, b) that every other pixel is not vasculature and c) if this is not the case an appropriate error needs to be given that describes the uncertainly of this measurement.
a) In the data a lot of random volumes are labeled as vasculature. They are clearly not vasculature, but rather pigments or so. How is this taken into account?
b) The vasculature appears to be disconnected. Have the authors tried to correct this? A vessel does not start and end somewhere random.
c) The data presented does not include any error calculations. Each data point is plotted as a point in the graphs. What is the error of each measurement given that a) and b) are not addressed?

2) The parameters that are computed in the manuscript need more explanation: e.g. what is vascular volume? Is this the volume of all segmented voxels? This would be inaccurate since the vessels are hollow and kdrl labels the walls of the vessels. Especially given the uneven fluorescence intensity of the marker the volume can only be estimated after proper segmentation including tracing the vasculature and computing the radius along the path. The same question applies to the surface (surface of blobs or hollow tubes along the vessel vs true vasculature surface), density, etc. All these depend strongly on the brightness of the signal and the quality of the segmentation, in articular given the hollow nature of the label. In my eyes, different terms need to be used here if they do not accurately describe the precise volume, density etc.

© 2022. Published by The Company of Biologists under the terms of the Creative Commons Attribution License (https://creativecommons.org/licenses/by/4.0/).

3) Using a threshold for segmentation means that higher autofluorescence or less specific labelling will yield a lot of false positives in segmentation. This needs to be taken into account when computing the error bar for the measurements.

4) The image processing literature is full of algorithms for vessel and neuron segmentation because this is a challenging task (several papers also in zebrafish). If the authors are trying to use a rather "quick and dirty" solution that is less accurate but faster, they need to acknowledge the shortcomings of their techniques and provide a comparison with proper (or manual) segmentation algorithms and give an error.

5) The authors use fish from 2dpf - 5dpf. Is there any reason for why they do not use older fish? Optical clearing would make older fish transparent and also offer superior image quality with potentially better segmentation results at 2-5dpf.

6) How can you compare different fish by registering and overlapping their data? Wouldn't a small shift in the data give you a mismatch? What if the same vessel is in a slightly difference place in one fish than the other? Shouldn't such comparisons only be made after skeletoniziation and branching analysis?

Minor comments:

1) General: In the zebrafish community, the use of morpholinos is highly debated and the use of morpholinos in this study needs to be justified and discussed. This may also have some implications for the results obtained with morpholinos.

2) Introduction: "LSFM datasets are often terabytes in size, rendering data handling, processing, and analysis computationally demanding." Is listed as the first of obstacles that need to be overcome. While the statement is correct, it is not a feature of the LSFM and is not justified here without giving any numbers.
The amount of data depends on the resolution of the camera, the number of z-planes, the number of colors, the number of timepoints etc. in a timelapse as in any other microscopy technique. It would be important to discuss what resolution, pixel size, chip size, field of view etc. are needed to sufficiently resolve the vasculature in the zebrafish and then make a calculation whether these are "terabytes of data" and whether the resulting data poses a problem. LSFM offers low phototoxicity and high acquisition speed. These features do not translate necessarily to large amounts of data if used wisely. And apparently the authors have been able to handle the amounts of data without a supercomputer. One of the next sentences "A further challenge…" needs an explanation. Not sure what the authors refer to.

---

**Resubmission**

MS ID#: DEVELOP/2021/199720

MS TITLE: Zebrafish Vascular Quantification (ZVQ): a tool for quantification of three-dimensional zebrafish cerebrovascular architecture by automated image analysis

AUTHORS: Elisabeth Christina Kugler, James Frost, Vishmi Silva, Karen Plant, Karishma Chhabria, Tim Chico, and Paul Armitage
ARTICLE TYPE: Research Article

© 2022. Published by The Company of Biologists under the terms of the Creative Commons Attribution License (https://creativecommons.org/licenses/by/4.0/).

Author response to reviewers' comments

**Point-by-point responses to the referees' concerns:**

*Reviewer 1 Advance Summary and Potential Significance to Field:*

In this study, Kugler et. al. develop a new computational pipeline to enable rigorous 3D quantitative image analysis of cerebrovascular architecture during zebrafish development. First, using images of the zebrafish cerebral vasculature acquired via light sheet microscopy, Kugler and colleagues apply image enhancement, segmentation and manual or automatic intersample registration to align embryo images and define areas of high similarity. Next, the authors establish a quantification workflow for automated acquisition of vessel volume, surface area, density, length, branch points, radius and complexity. Then they apply this workflow to quantify architectural changes occurring (1) during normal cerebrovascular development, (2) upon loss of blood flow, (3) following perturbation of vascular gene expression / function, and (4) in distinct regions of the brain. Moreover, they have made this pipeline publicly available as an online tool available via open-source Fiji software. As such, Kugler et. al. has generated a useful resource that may be adopted by many in the vascular biology community.
Although the function of this tool is not in doubt, what is less clear is the significance of the insights that can be generated using this workflow. Most importantly, the quantified phenotypes described in this study are somewhat superficial and lack precise details of the brain regions involved, specific vessels affected, persistence of the observed changes or underlying changes in cell behavior responsible for these events. Without further investigation of the sensitivity of this tool and rigorous confirmation of its ability to pinpoint region-specific subtle phenotypes that would normally be missed, the broad utility of this pipeline and level of insight it can generate over pre- existing methods remains unclear.

*Reviewer 1 Comments for the Author:*
Whilst I am overall positive about this manuscript, there are several points that I consider to be sufficiently important that they should be addressed with revisions prior to acceptance for publication:
(1) Kugler and colleagues begin the study by stating that the lack of robust automated approaches to quantify vascular anatomy in 3D prevents detection of subtle phenotypes. However, a question still remains as to the sensitivity of their analytical workflow and whether it can truly detect subtle changes. Several embryo manipulations appear to elicit little to no effect on cerebrovascular architecture using their pipeline (e.g. notch 1b knock-down, LNAME, Wnt inhibition, Wnt activation, osmotic pressure, 0.25% DMSO, left-right patterning). This is somewhat surprising and, in some cases, inconsistent with the literature (e.g. Wnt). As such, it is possible that this tool only detects severe changes in cerebrovascular architecture, which may be easy to detect by conventional manual images analyses. Indeed, many of the manipulations used to validate the workflow induce severe vascular defects that are easy to detect (e.g. lack of blood flow, VEGF inhibition, Notch inhibition, actin/ myosin inhibition, etc). The authors need to provide additional evidence that this tool is sufficiently sensitive to detect subtle phenotypes and outperforms other forms of image analysis in doing this. In particular, use of several positive control manipulations that are already known to induce subtle cerebrovascular phenotypes (e.g. loss of cxcl12b, loss of cxcr4a, inhibition of Wnt using IWR-1) should be analyzed to confirm that their tool can robustly detect these. Otherwise, the broader utility of this tool and its improvement on other approaches are unclear.
**Our response:** We thank the reviewer for their insightful comments and have restructured the entire manuscript to address their concerns. We now include group percentage differences to show the ability in detecting subtle biological differences. In the revised manuscript, data on vascular development are shown first to demonstrate the ability to detect subtle differences between age groups. Next, we examine tnnt2a MO data which are known to show severe inter-group differences in fish of the same age and following analyse the impact of short-term low-dose VEGF inhibition which results in subtle inter-group differences. This is followed by showing for the first time that we can quantitatively assess differences for data where cerebrovascular hypo- or hyper-vascularization were assumed but never quantified (i.e. inhibition Notch, Actin polymerization, Myosin II), then move on to data which were previously suggested to show no difference (i.e. glucose and DMSO). Lastly, we examine morpholino data, for morpholinos which were all previously confirmed. Together, our data confirmed 8 previous findings/assumptions

© 2022. Published by The Company of Biologists under the terms of the Creative Commons Attribution License (https://creativecommons.org/licenses/by/4.0/).

(development, VEGF inhibition, Notch inhibition, Actin inhibition, Myosin II inhibition, DMSO treatment, glucose treatment, *tnnt2a* MO), provides new insights into 4 proteins (*jagged1a, jagged1b, ccbe1, notch1b*) and highlights *dll4* to be further examined, providing for the first time quantitative insights and rate of change for all the examined data. With these additional changes, we hope to have highlighted that we are able to detect subtle differences, as would be the case for cxcl12b, cxcr4a, IWR-1, etc.
We removed data on NOS inhibition, Wnt activation, and Wnt inhibition, as we believe that with longer treatments or higher concentrations these might show an effect, but are currently not able to examine this further.

(2) The output of the workflow is somewhat superficial and does not generate much insight into the cause of observed defects, other than to describe global shifts in the analyzed parameters. Evidence that the pipeline can be exploited to define specific underlying phenotypes (i.e. as a route to understanding gene function) is essential to confirm that this tool "will aid discovery of novel insights into vascular development". For example, can the workflow be modified to define the precise location of extra or lost branch points? Similarly, can it define specific zones of increased complexity, surface area, radius, density or volume? Can it determine where length been added to or lost from the Wt vasculature? If vessel similarity is decreased, which specific regions are losing similarity? Can the tool determine if all of the above defects are associated with certain vascular beds, specific vessels or even arterial verses venous tissues? Can the specific location of these defects then be mapped to patterns of gene expression, such as DLL4, CXCR4, VEGFRs? Addressing these questions will determine how informative the tool can be, otherwise, the insights generated by this workflow are not so compelling.
**Our response:** We appreciate that the reviewer would like to see outputs for individual vessels delivered, rather than "global" shifts to be analysed.
However, this is computationally a very challenging task and currently beyond the scope of the tool. In the future, it would be possible to extend this once appropriate atlases are available, as it was recently shown for mouse data Todorov et al., 2020
https://www.nature.com/articles/s41592- 020-0792-1.
In the revised manuscript we discuss the challenges of vessel-specific analysis. Briefly, the currently available brain atlases are for fish at 6dpf (Randlett et al 2015 https://www.nature.com/articles/nmeth.3581; Kunst et al 2019 https://www.sciencedirect.com/science/article/pii/S0896627319303915; https://fishatlas.neuro.mpg.de/) and none exists for 3dpf or different developmental stages. This would be a requirement to annotate vessels based on their anatomical location robustly.

In the revised manuscript we included Fig. S18 to examine regional differences, studying semi-automatic vessel annotation from templates using the simple neurite tracer, but found this to be extremely labour intensive and not feasible for all examined sample groups (ie 2-5dpf, 2 experimental groups in 9 chemical treatments; 3 experimental groups in 6 morpholino datasets).

We hope that by sharing code, documentation, and data, this will be picked up by the community and collaborative efforts could produce such an atlas.
As for arterio-venous specification/selection: This could be addressed, again with an atlas, for example by using double-transgenic lines which allow to distinguish arteries from veins. Similarly, expression patterns could be back-mapped using registration. However, this was beyond the scope of this study.
Additionally, the implemented workflow is modular (newly included Figure in section 1 of the workflow documentation to make this clearer), allowing the user to choose which steps to be performed. E.g. if the user wants to perform LR analysis they can do this on unregistered samples (step (6) in the analysis workflow by providing manually drawn line ROIs) or on registered samples (step (7) in the analysis workflow by drawing a mask).
Thus, users could identify zones of interest using global comparisons first and then run the quantification analysis on their sub-region of interest.

(3) At the start of page 5, Kugler et. al. state that image registration improves the reproducibility of quantification. However, there is not adequate investigation of this. A key novelty of the workflow is the use of this initial image inter-sample registration, and all the other image analysis methods used appear to be standard approaches that are routinely applied to

© 2022. Published by The Company of Biologists under the terms of the Creative Commons Attribution License (https://creativecommons.org/licenses/by/4.0/).

non-registered samples. As such, the authors need to demonstrate how essential the image registration is to the subsequent workflow analysis. What happens to the variability of measurements for the other 7 parameters quantified in this study if images are not pre-registered? Does image registration truly improve the reproducibility of this quantification?
**Our response:** We could not find the particular section the reviewer referred to. The aim of the presented inter-sample registration approach is to be able to compare fish against each other in terms of regions of similarity and variability. However, we considered the reviewers suggestion and included Figure **S5A-F** (Bland-Altman test pre and post registration) as well as CoV analysis to examine the impact of registration on the quantified parameters.
One main advantage of applying registration prior to quantification is that a ROI can be manually drawn on the template and applied to all samples, replacing the need to draw ROIs for each fish individually. Thus, reducing time investment of researchers as well as potential bias.
We would also like to point out that inter-sample registration is not essential to the workflow, as the individual steps (1-7 see GUI and workflow documentation) are implemented in a modular fashion. To clarify this, we included an additional figure in section 1 of the accompanying workflow documentation.

*Minor comments:*
(1) Kugler et. al. frequently refers to genetic manipulations. However, I did not identify any genetic manipulations in this study.Please re-word.
**Our response:** Corrected this in the manuscript.

(2) In some figures, quantification of all 8 metrics is absent. (e.g. no surface area or density in Fig.3, which would be expected to increase) Why are some omitted? Please make reference to all measurements for consistency and to enable a full comparison between embryo manipulations.
**Our response:** We rectified this mistake and quantified vascular surface and density for the developmental dataset (Fig. 3), as well as changed the according section in the text.

*Reviewer 2 Advance Summary and Potential Significance to Field:*

This manuscript by Kugler et al. presents an analytical pipeline for 3D quantification of cerebral vasculature for larval zebrafish. The authors first describe registration approaches (landmark based and automatic rigid registration) of light-sheet microscopy data of cerebral vasculature to bring 3D images from different samples into a common co-ordinate system. Using these registered images, the authors then quantify various vasculature parameters such as similarity, volume, network length, etc. to describe how these properties change during development from 2
– 5 dpf. Next, applying a range of genetic and pharmacological manipulations that could impact cerebral vascular development, the authors employ their pipeline to characterize change in vasculature properties compared to controls. Finally, the authors also provide a comparison of vasculature parameters between mid/hindbrain and L-R sides of the brain. The authors have developed the pipeline with a Graphical User Interface in the widely used open source image processing software Fiji, and provided source code for their pipeline.
Broadly, I think the manuscript tries to address an essential gap in 3D analyses of vasculature parameters across the brain. This could potentially be valuable for the field, and not just for those working with zebrafish as a model system. However, due to the concerns I highlight below, my enthusiasm for publishing the manuscript in its current form is diminished. I have a few major conceptual concerns with the manuscript and some minor concerns, that the authors should address before publication.

*Reviewer 2 Comments for the Author:*

*Major concerns:*
(i) The authors employ rigid registration to bring different samples to the same co-ordinate system. However, as is evident from Fig. S2 (A), (B) and very low values for Dice coefficient (similarity scores) throughout the manuscript, the rigid registration performs poorly. CMTK plugins for Fiji (ImageJ) allow employing multi-step registration with both rigid and non- rigid (affine or other) approaches. The primary purpose of being able to register different samples to a common reference frame is to be able to spatially

© 2022. Published by The Company of Biologists under the terms of the Creative Commons Attribution License (https://creativecommons.org/licenses/by/4.0/).

localize vascular feature changes, which does not come across in the manuscript. The authors should address this aspect, if possible, by including non-rigid, more accurate registration in their pipeline.

**Our response:** We thank the reviewer for their insightful comments and examined these further, as suggested.

We were unable to perform successful non-rigid registration with any of the available methods (including CMTK, thin-plate landmark bases, etc., See Fig. 2 I,J), we believe due to the sparse nature of zebrafish vascular datasets. It may be possible to develop a bespoke registration methodology in the future, or utilise double transgenics that include more structural information, but this is beyond the scope of current work. Non-rigid registration will also introduce additional problems where the topology differs between sample and template and will require a different analysis approach, such as tensor based morphometry, as the registration process will remove the inter-sample differences that we wish to measure. We believe that we have shown that there is sufficient similarity between samples (e.g. Fig. 3) to facilitate reliable regional analysis of the zebrafish vasculature, as proposed in this workflow. Indeed, our Bland-Altman analyses have shown that the method reliably calculates vascular parameters using this approach, when compared to manual region placement.

(ii) The Dice coefficient is used throughout the manuscript as a measure to interpret similarity within samples and between samples that underwent different genetic or pharmacological manipulations. However, as pointed out in (i), the variance and the values of Dice coefficient themselves could simply be a result of the registration quality. Therefore, using similarity between samples that have undergone different treatments to interpret mechanistic differences has little meaning. The authors should avoid using the Dice coefficient as a measure of changes in vascular properties.

**Our response:** We additionally examined whether other structural similarity measurements would be more appropriate for our data (including Jaccard index, Total Overlap, Mutual information, mean square difference, sum of squared difference, and structural similarity; **Fig. S5G-M**), finding none of them to perform “better” than the Dice coefficient. We have added a section to the manuscript describing these findings. While the similarity scores may be low, we believe that they still provide useful information on vascular similarity, reflecting the overall variability in the vasculature, even though we have shown that many larger vessels show remarkable consistency. We also believe that leaving the similarity score in the workflow is worthwhile, as it will provide a benchmark against which to compare future developments in registration methodology.

(iii) While the authors correctly state that registered volumes in the same coordinate system can be used to compare vascular parameters between brain regions or left-right symmetry, it is not clear if the implementation of these anatomical masks is a part of the pipeline. Figure 1 and Figure 6 seem to suggest this. However, it becomes even more relevant considering the genetic or pharmacological manipulations included in this study, to include this analysis as these signalling pathways are known to impact control of symmetry and topology along the anterior- posterior or dorso-ventral or left- right axes during development. If this analysis is part of the pipeline, why was it not performed for all manipulations or for developmental snapshots, instead of presenting average vasculature parameters across the entire brain? Surely, quantifying these parameters by brain region or by comparing symmetry for all manipulations, the authors could add a much needed novel component to their analysis pipeline and this study, thereby helping the community gain discovery-based mechanistic insights! It is critical that the authors address this aspect.

**Our response:** The implemented workflow is modular (newly included Figure in section 1 of the workflow documentation to make this clearer), allowing the user to choose which steps to be performed. E.g. if the user wants to perform LR analysis they can do this on unregistered samples (step (6) in the analysis workflow by providing manually drawn line ROIs) or on registered samples (step (7) in the analysis workflow by drawing a mask).

Although we appreciate that additional quantification of LR and mid-to-hindbrain differences would likely provide novel biological insights, this was not performed as this manuscript is (a) focused on the methods development and (b) additional quantification of these parameters would require approximately 487 hours of computing (45 min * >650

© 2022. Published by The Company of Biologists under the terms of the Creative Commons Attribution License (https://creativecommons.org/licenses/by/4.0/). 

embryos). Additionally, requiring statistical data analysis and data representation of a further 256 graphs (16 datasets * 8 parameters * 2 (for LR and mid-to-hindbrain)). Resulting in roughly 92 (743 hours / 8) working days to purely do this.
Regarding "as these signalling pathways are known to impact control of symmetry and topology along the anterior-posterior or dorso-ventral or left- right axes during development" – the authors are unsure which pathways the reviewer is referring to, particularly with regards to the vasculature, as the authors did not find any literature on this and would be grateful if the reviewer could expand on this.
Lastly, the authors are happy to provide datasets to the community, should a researcher be interested in examining a particular (or all) datasets.

*Minor concerns:*

i. Which datasets were specifically acquired for this manuscript and which are being re-used from previous studies by the authors? This does not come across clearly. This aspect should be made very clear both in the Results and Methods section.
**Our response:** We clarified this in the text.

ii. Often, references to the Methods section or a brief explanation of the analysis is lacking in the Results section (for example: it is not clear what the output of the Sholl analysis is? Or how was the clustering in Figure 5 performed and validated? Or was it validated for the optimal number of clusters?). This should be rectified throughout the manuscript. This will help the authors reach a broader audience.
**Our response:** We added additional information on cluster analysis and validation in the Material and Methods section. We expanded on Sholl analysis in the supplementary figures (**Fig. S4**).

iii. The hyperlink to the test dataset in the supplemental section is restricted. While it is very good that the authors provide not only the source code for their pipeline but also provide example datasets, this should be made easily accessible to all readers on an open repository.
**Our response:** We rectified this by depositing example data to Zenodo (doi 10.5281/zenodo.4108660; https://zenodo.org/record/4108660#.X47XU9BKizc).

iv. The color scales for 'similarity' for MIPs across all figures do not have values next to them. The values should be displayed, and all MIPs scaled to a common value range (i.e. for example, all MIPs should be between 0 – 0.3 on the color scale). This will make comparison across figures possible.
**Our response:** Rectified in the manuscript to 0-255 value range.

v. It should also be made clear in the Methods section how and which template (or target) 3D volumes were chosen for registration across all treatments. For example, the tnnt2a morpholino injections result in very little structure being present in cerebral vasculature. Does this influence template selection and therefore registration accuracy?
**Our response:** As there is currently no standard reference brain/registration template for zebrafish, all embryos were registered to a template from the control group while ROIs for quantification were drawn for one sample/template per group. This was performed to avoid biasing of results in cases where cerebral growth might be altered as it is the case in for example zebrafish without blood flow. Future work might examine the establishment of standard templates for registration, considering age differences (minimum intervals 12h suggested) as well as treatment/manipulation impacts.

*Reviewer 3 Advance Summary and Potential Significance to Field:*
Kugler et al present a workflow and software to analyze the zebrafish vasculature in LSFM data. The authors use the workflow to quantify the vasculature in wt and morphant zebrafish. The manuscript is well written and presents some valuable data. However, it is superficial and lacks the necessary controls and error estimation. The segmentation is only an approximation of the vasculature and cannot be compared without proper error estimation.

*Reviewer 3 Comments for the Author: Major comments:*

© 2022. Published by The Company of Biologists under the terms of the Creative Commons Attribution License (https://creativecommons.org/licenses/by/4.0/).

(i) This may be the "first easily applicable 3D image analysis pipeline for zebrafish vasculature" - but is it accurate? Before going into the various experiments, it is important to show that the vasculature is correctly segmented. "Image segmentation is the process of assigning a label to every pixel in an image such that pixels with the same label share certain characteristics."(Wikipedia) This means in this case that a) every voxel that is labeled as vasculature is indeed vasculature, b) that every other pixel is not vasculature and c) if this is not the case an appropriate error needs to be given that describes the uncertainly of this measurement.

**Our response:** We thank the reviewer for their feedback and appreciate that the main concern of this reviewer was that the subsequent image analysis steps depend on a robust segmentation approach. As we feel the same about this (and referred to it multiple times in the manuscript), we have spent a significant amount of time on data understanding, enhancement, and segmentation methodology development, producing 3 peer reviewed manuscripts:

(a) Kugler E., Chico T. and Armitage P. Validating Segmentation of the Zebrafish Vasculature, In: Zheng Y., Williams B., Chen K. (eds) Medical Image Understanding and Analysis. MIUA 2019. Communications in Computer and Information Science, vol 1065. Springer,
Cham

(b) Kugler E., Plant K., Chico T. and Armitage P., Enhancement and Segmentation Workflow for the Developing Zebrafish Vasculature, Journal of Imaging, MDPI, Vol. 5, Issue 1 2019

(c) Kugler E., Chico T. and Paul A., Image Analysis in Light Sheet Fluorescence Microscopy Images of Transgenic Zebrafish Vascular Development, In: Nixon, M., Mahmoodi, S. and Zwiggelaar, R., (eds.) Medical Image Understanding and Analysis. MIUA 2018, 09-11 Jul 2018, Southampton, UK. Communications in Computer and Information Science, 894 . Springer Nature Switzerland AG , pp. 343-353. ISBN 9783319959207

In addition, we have performed a detailed validation of our segmentation methodology for the zebrafish brain vasculature, which is currently under review elsewhere.

(d) Elisabeth C. Kugler, Andrik Rampun, Timothy J.A. Chico, Paul A. Armitage, Segmentation of the Zebrafish Brain Vasculature from Light Sheet Fluorescence Microscopy Datasets, bioRxiv 2020.07.21.213843; doi: https://doi.org/10.1101/2020.07.21.213843

We expanded on this in the manuscript to clarify these concerns.

(ii) In the data a lot of random volumes are labeled as vasculature. They are clearly not vasculature, but rather pigments or so. How is this taken into account?

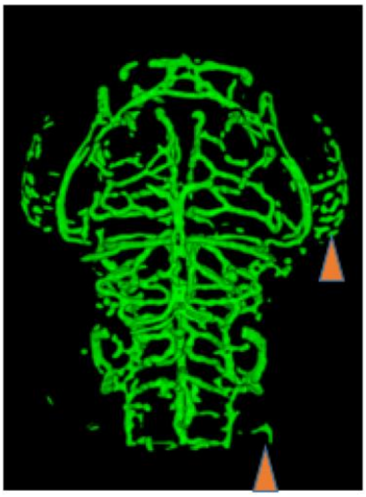

© 2022. Published by The Company of Biologists under the terms of the Creative Commons Attribution License (https://creativecommons.org/licenses/by/4.0/). 

**Our response:** We are not entirely sure which random volumes the reviewer refers to, but assume these are mainly observed in the eye and peripherally (e.g. fins), which are regions that are generally excluded from the analysis by manual or automated ROI selection. Tg(kdrl:HRAS-mCherry) very specific signal, which would not be the case in cases with fluorophore expression under pan-endothelial promotors (as shown in Kugler et al. 2020 (BioRxiv 10.1101/2020.07.21.213843, Figure 8).
Future work might address this further by connected component analysis or size exclusion. However, reanalysis of the presented data was not performed due to time limitations.

(iii) The vasculature appears to be disconnected. Have the authors tried to correct this? A vessel does not start and end somewhere random.

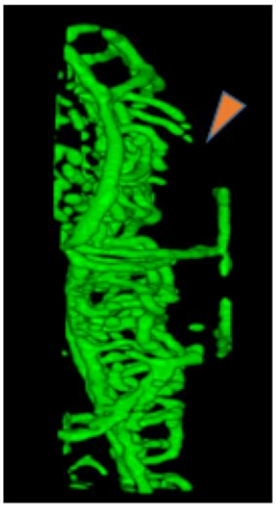

**Our response:** We appreciate the reviewers comment and found disconnectedness to particularly effect the most peripheral/dorsal vessels (indicated below) which constitute 8 out of approximately 100 vessels at 3dpf.
Our analysis was performed on standard data derived from a different study and disconnectedness is most likely due to locally reduced signal caused by pigmentation. However, data quality could be improved by (a) application of the chemical PTU, (b) using zebrafish lines without pigmentation, (c) multi-view image acquisition. Overall, our aim here was to provide a tool for the community to quantify their data, and in reality we appreciate that “optimum image quality” is barely ever reached.

Additionally, we have examined segmentation methods which are based on statistical similarity and connectedness (Kugler et al., 2019 MDPI) previously. However, we found these to (a) deliver less satisfactory segmentation results than Otsu based thresholding, and (b) be computationally more demanding (particularly, level set segmentation which is a promising method in medical image analysis; which would have been otherwise optimised for segmentation of our data).
We thus further examined deep-learning based methods for vascular segmentation in Kugler et al. 2020 (BioRxiv 10.1101/2020.07.21.213843, Figure 8). However, the trained methods were at this time only examined for their applicability to one particular age (3dpf).
We included a section on this in the discussion.

(iv) The data presented does not include any error calculations. Each data point is plotted as a point in the graphs. What is the error of each measurement given that a) and b) are not addressed?

**Our response**: The authors are unsure to which graphs the reviewer is referring to, as all graphs have error bars. Should the reviewer refer to error calculations in segmentation outcomes, we would like to refer them again to the above mentioned manuscripts in which we assess segmentation quality.

© 2022. Published by The Company of Biologists under the terms of the Creative Commons Attribution License (https://creativecommons.org/licenses/by/4.0/).

(v) The parameters that are computed in the manuscript need more explanation: e.g. what is vascular volume? Is this the volume of all segmented voxels? This would be inaccurate since the vessels are hollow and kdrl labels the walls of the vessels. Especially given the uneven fluorescence intensity of the marker the volume can only be estimated after proper segmentation including tracing the vasculature and computing the radius along the path. The same question applies to the surface (surface of blobs or hollow tubes along the vessel vs true vasculature surface), density, etc. All these depend strongly on the brightness of the signal and the quality of the segmentation, in articular given the hollow nature of the label. In my eyes, different terms need to be used here if they do not accurately describe the precise volume, density etc.

**Our response:** The reviewer raises the concern about "uneven excitation": We examine this in Kugler et al 2019 (MDPI) Figure 2, showing that the signal is not statistically significant different in different vessels (small-large, ventral-dorsal) and over time (2-5dpf). Additionally, we show in Kugler et al 2020 (bioRxiv) Figure 5 that experimental and augmented contrast-to-noise ratio (CNR) changes do not statistically significantly impact segmentation outcomes.

The reviewer also points out that vessels are hollow. However, this is only partially true, as lumenized and unlumenized vessels exists and a segmentation approach needs to take both cases into account. To achieve this, we are using a vascular enhancement approach which converts all vessels into filled tubes with cross-sectional single-peak intensity distributions (see Kugler et al 2019 (MDPI) Figure 4; Kugler et al 2020 (bioRxiv) Figure 3).

(vi) Using a threshold for segmentation means that higher autofluorescence or less specific labelling will yield a lot of false positives in segmentation. This needs to be taken into account when computing the error bar for the measurements.

**Our response:** We examined various segmentation approaches in Kugler et al 2019 (MDPI) Figure 5, finding that intensity-based thresholding using Otsu thresholding is the most applicable to our data. Prior to segmentation, the method of Sato et al. was used to enhance tubular structures at a scale optimised for the zebrafish vasculature and minimise the contributions from non-vessel structures.

(vii) The image processing literature is full of algorithms for vessel and neuron segmentation because this is a challenging task (several papers also in zebrafish). If the authors are trying to use a rather "quick and dirty" solution that is less accurate but faster, they need to acknowledge the shortcomings of their techniques and provide a comparison with proper (or manual) segmentation algorithms and give an error.

**Our response:** We hope that the reviewer sees that the authors have not presented a "quick and dirty" segmentation approach, but an approach which has undergone rigorous peer review at multiple stages of its development.

(viii) The authors use fish from 2dpf – 5dpf. Is there any reason for why they do not use older fish? Optical clearing would make older fish transparent and also offer superior image quality with potentially better segmentation results at 2-5dpf.

**Our response:** As mentioned in the manuscript, the data analysed here were reused from a previous biological publication by Kugler et al. 2019 (EMBO Reports). We here focus on in vivo data analysis prior to 5.2dpf due to multiple reasons (a) most zebrafish laboratories examine vascular development prior to 5.2dpf; which is partially also due to animal research regulations AS(P)A which classify zebrafish experiments above 5.2dpf as animal. Thus, for ethical reasons we did not sacrifice animals purely for this study. (b) The authors would like to argue against the reviewers comment that the image quality would be superior due to optical clearing as (i) the embryos are transparent (and could be treated with PTU, or samples could be raised with a nacre background to further reduce pigmentation), (ii) the vasculature is prone to collapsing during the fixation process, and (iii) intrinsic fluorophores could be quenched in the process.

(ix) How can you compare different fish by registering and overlapping their data? Wouldn't a small shift in the data give you a mismatch? What if the same vessel is in a slightly difference place in one fish than the other? Shouldn't such comparisons only be made after skeletoniziation and branching analysis?

**Our response:** We examined this further in Fig. S5 , S18, and S19 added an additional section in the discussion. We furthermore examined affine and thin-plate landmark-

© 2022. Published by The Company of Biologists under the terms of the Creative Commons Attribution License (https://creativecommons.org/licenses/by/4.0/).

based registration, finding neither of those applicable to our data with current implementations (**Fig. 2I,J**). As the main aim of this study was to compare different embryos to each other, we show for the first time that some vessels show extensive overlap, while others do not. We anticipate that non-rigid registration might remove these inter-sample differences by overfitting the registration, thus, potentially removing biologically important inter-sample differences. In this work, the registration methodology facilitates automated regional analysis of vascular properties, whereby the regions are large enough to minimise the effects of small registration errors.

*Minor comments:*

(i) General: In the zebrafish community, the use of morpholinos is highly debated and the use of morpholinos in this study needs to be justified and discussed. This may also have some implications for the results obtained with morpholinos.

**Our response:** We restructured the entire manuscript to address this concern. We now show data of vascular development first, to validate the ability to detect subtle differences between age groups, next we examine tnnt2a MO data which are known to show severe inter-group differences in fish of the same age. We next show for the first time that we can quantitatively assess differences for data which cerebrovascular hypo- or hyper-vascularization were assumed but never quantified (i.e. inhibition of VEGF, Notch, Actin polymerization, Myosin II), then move on data which were previously suggested to show no difference (i.e. negative controls; glucose and DMSO). We examine morpholino data in only a small part of the study for morpholinos which were all previously confirmed. Together, our data confirmed 8 previous findings/assumptions (development, VEGF inhibition, Notch inhibition, Actin inhibition, Myosin II inhibition, DMSO treatment, glucose treatment, *tnnt2a* MO), provides new insights into 4 proteins (*jagged1a, jagged1b, ccbe1, notch1b*) and highlights *dll4* to be further examined, providing for the first time quantitative insights and rate of change for all the examined data.
We removed data on NOS inhibition, Wnt activation, and Wnt inhibition, as we believe that these longer treatments or higher concentrations might show an effect, but are currently not able to examine this further.

(ii) Introduction: "LSFM datasets are often terabytes in size, rendering data handling, processing, and analysis computationally demanding." Is listed as the first of obstacles that need to be overcome. While the statement is correct, it is not a feature of the LSFM and is not justified here without giving any numbers. The amount of data depends on the resolution of the camera, the number of z- planes, the number of colors, the number of timepoints etc. in a timelapse as in any other microscopy technique. It would be important to discuss what resolution, pixel size, chip size, field of view etc. are needed to sufficiently resolve the vasculature in the zebrafish and then make a calculation whether these are "terabytes of data" and whether the resulting data poses a problem. LSFM offers low phototoxicity and high acquisition speed. These features do not translate necessarily to large amounts of data if used wisely.

**Our response:** We rectified this comment by inclusion of information on this in the introduction.

(iii) And apparently the authors have been able to handle the amounts of data without a supercomputer. One of the next sentences "A further challenge…"

**Our response:** This section was expanded by the authors.

© 2022. Published by The Company of Biologists under the terms of the Creative Commons Attribution License (https://creativecommons.org/licenses/by/4.0/).

First decision letter

MS ID#: DEVELOP/2021/199720

MS TITLE: Zebrafish Vascular Quantification (ZVQ): a tool for quantification of three-dimensional zebrafish cerebrovascular architecture by automated image analysis

AUTHORS: Elisabeth Christina Kugler, James Frost, Vishmi Silva, Karen Plant, Karishma Chhabria, Tim Chico, and Paul Armitage

Many apologies for the long delay in making a decision on your manuscript. I was waiting on one reviewer who was unfortunately not able to submit their review. Consequently, I have made a decision based on the two referees' comments we did receive. You can access them below or online: please go to BenchPress and click on the 'Manuscripts with Decisions' queue in the Author Area.

As you will see, one of the referees thinks that although the manuscript could be further strengthened through addition of registration analysis, he/she considers that the study can be published in its current form. The second reviewer still has two significant concerns that need to be addressed prior to publication. Please attend to these comments and ensure that you clearly highlight all changes made in the revised manuscript. Please avoid using 'Tracked changes' in Word files as these are lost in PDF conversion. I should be grateful if you would also provide a point-by-point response detailing how you have dealt with the points raised by the reviewers in the 'Response to Reviewers' box. If you do not agree with any of their criticisms or suggestions please explain clearly why this is so.

Reviewer 1

*Advance summary and potential significance to field*

In this revised version of their manuscript, Kugler et. al. again presents a detailed description and validation of the new computational pipeline that they have developed to enable rigorous 3D quantitative image analysis of cerebrovascular architecture during zebrafish development. As detailed in my previous assessment of the original manuscript, Kugler et. al. has generated a useful resource, but my previous main concerns were that the sensitivity and utility of this tool, and overall benefit of image registration in this pipeline, were unclear. As such the usefulness of this pipeline and level of insight it could generate over pre-existing image analysis methods were in doubt. To address these comments, Kugler et. al. has added some further analysis of the impact of image registration on data output, as well as clarification of the ability of this pipeline to define vascular regions of similarity / variability. Moreover, Kugler et. al. has removed or rearranged several data panels in direct response to my previous comments. Despite these changes, I feel that the revised manuscript does not fully address my original concerns about the sensitivity and utility of this tool, and requires further revision to address these issues.

*Comments for the author*

As mentioned in my previous review of this manuscript, I am overall positive about the study. However, several of my previous comments have not been sufficiently addressed in this revised manuscript. These are points that I consider to be sufficiently important that they should be addressed with revisions prior to acceptance for publication:
(1) A question still remains as to the sensitivity of their analytical workflow and whether it can truly detect subtle changes in vascular architecture. I previously raised the concern that several manipulations elicit little to no effect on cerebrovascular architecture using their pipeline, which was surprising and, in some cases, inconsistent with the literature (e.g. Wnt). These points raised concerns that the tool can only detects severe changes in cerebrovascular architecture, which may be easy to detect by conventional manual image analyses. To address this, I suggested that the authors provide additional evidence that the tool is sufficiently sensitive to detect subtle phenotypes induced by known manipulations (e.g. loss of cxcl12b, loss of cxcr4a, inhibition of Wnt using IWR-1) and that it outperforms other forms of image analysis in

© 2022. Published by The Company of Biologists under the terms of the Creative Commons Attribution License (https://creativecommons.org/licenses/by/4.0/). 

doing this. Rather than adding new data to directly address this point and confirm the sensitivity of the tool, the authors have instead removed the several figures of data that raised these initial concerns about tool sensitivity. In addition, wording of the text has been altered to further strengthen their point that the tool detects “subtle” biological differences (for example, over developmental time or upon brief VEGF inhibition). Yet, I am not entirely convinced that these detected differences are subtle and wouldn't be easily detected using other analysis methods. Without adding new data validating that their pipeline can identify known subtle phenotypes described elsewhere, the broader utility of this tool and its improvement on other approaches are still unclear.

(2) Another remaining concern that has not been fully addressed is that the output of the workflow is somewhat superficial and does not generate much insight into the cause of observed defects (other than to describe global shifts in the analyzed parameters). For example, a decrease in vessel similarity is observed upon loss of dll4 expression (Fig. S13), but what this actually reflects at the level of vascular architecture remains obscure. Can this observation be traced back to manipulation-induced variance in specific regions or vessels? This is essential as a route to defining gene or pathway function, otherwise insights generated by this workflow are not particularly informative. Similarly, it is critical to understand why vascular volume and surface volume are specifically elevated upon DAPT treatment – does this indicate wider vessel lumens? And if so, in which regions?
Likewise, if network length is reduced upon loss of jagged-1b, are all vessels slightly reduced in length or does this reflect a reduction in length (or loss) of specific vessels? Perhaps even more puzzling is the transient dip in vessel surface volume seen at 4dpf (Fig. 2F) and transient increase in vessel density observed at 3dpf (Fig. 2G) during normal embryonic development. This data was excluded from the original submission and added upon request, but raises a key question as to the robustness of the analysis tool. Can these unexpected shifts be explained by specific developmental stage-dependent changes to vessel shape and/or topology (which can then be clearly indicated in a representative image), or do they simply reflect noise or error in the data acquisition or analysis pipeline. Hence, rather than only providing the 8x pipeline output values, which only provide superficial insight, this needs to be followed up with a clear description of the underlying cause of this parameter shift to (1) confirm the utility of the tool as a means to generating informative insights into gene or pathway function, and (2) validate the robustness of the analysis pipeline to confirm that any unexpected shifts in output values are truly meaningful and of biological significance (i.e. not artifactual).

Minor points:
(1) I previously commented that the statement in the text suggesting that image registration improves the “reproducibility of quantification” was not experimentally explored. However, the authors claim that they could not find this section, despite it still being included in the text (now on page 7). This statement needs to be reworded.
(2) Several references to Figures in the main text refer to the incorrect figure – for example references to Fig. S2C-G and Fig. 3D on page 6, and Fig. 4A on page 8.
(3) Figure S3, S5 and S16 are not referred to or described in the main text.

Reviewer 2

*Advance summary and potential significance to field*

The authors have developed an image analysis pipeline for the alignment and analysis of brain vasculature in 3D for larval zebrafish. The pipeline supports alignment and quantification of several vasculature parameters for whole brain datasets in larval zebrafish. This could be a potentially useful tool for many labs in the field, and would require little computational expertise. The tool development addresses a gap in the field for statistically driven analysis to map changes in vasculature between different samples/genotypes.

*Comments for the author*

The authors have addressed/clarified most of the comments from the previous review. I do appreciate that some fundamental/conceptual challenges persist and would be hard to address

© 2022. Published by The Company of Biologists under the terms of the Creative Commons Attribution License (https://creativecommons.org/licenses/by/4.0/).

within the scope of this manuscript/project. I am satisfied with the manuscript in its current form and would be happy to proceed with publication. The authors have also made an effort to ensure that their pipeline/test datasets are more openly accessible to the community.
I do however strongly suggest/recommend that the authors in the future develop standardized reference brains and explore elastic/diffeomorphic registration approaches and explore robust parameters for true 3D analysis of their datasets. That will render their approach much more powerful than their current pipeline for a robust analysis of brain vasculature.
That said, the current tool/analysis pipeline should benefit researchers in the community for a preliminary analysis of their 3D datasets without requiring extensive computational training.

---

**First revision**

Author response to reviewers' comments

***Response to reviewers comments***

***Reviewer 1 Advance Summary and Potential Significance to Field:***
In this revised version of their manuscript, Kugler et. al. again presents a detailed description and validation of the new computational pipeline that they have developed to enable rigorous 3D quantitative image analysis of cerebrovascular architecture during zebrafish development. As detailed in my previous assessment of the original manuscript, Kugler et. al. has generated a useful resource, but my previous main concerns were that the sensitivity and utility of this tool, and overall benefit of image registration in this pipeline, were unclear. As such, the usefulness of this pipeline and level of insight it could generate over pre-existing image analysis methods were in doubt. To address these comments, Kugler et. al. has added some further analysis of the impact of image registration on data output, as well as clarification of the ability of this pipeline to define vascular regions of similarity / variability. Moreover, Kugler et. al. has removed or rearranged several data panels in direct response to my previous comments. Despite these changes, I feel that the revised manuscript does not fully address my original concerns about the sensitivity and utility of this tool, and requires further revision to address these issues.

Reviewer 1 Comments for the Author:
As mentioned in my previous review of this manuscript, I am overall positive about the study. However, several of my previous comments have not been sufficiently addressed in this revised manuscript. These are points that I consider to be sufficiently important that they should be addressed with revisions prior to acceptance for publication:

(1) A question still remains as to the sensitivity of their analytical workflow and whether it can truly detect subtle changes in vascular architecture. I previously raised the concern that several manipulations elicit little to no effect on cerebrovascular architecture using their pipeline, which was surprising and, in some cases, inconsistent with the literature (e.g. Wnt). These points raised concerns that the tool can only detects severe changes in cerebrovascular architecture, which may be easy to detect by conventional manual image analyses. To address this, I suggested that the authors provide additional evidence that the tool is sufficiently sensitive to detect subtle phenotypes induced by known manipulations (e.g. loss of cxcl12b, loss of cxcr4a, inhibition of Wnt using IWR-1) and that it outperforms other forms of image analysis in doing this. Rather than adding new data to directly address this point and confirm the sensitivity of the tool, the authors have instead removed the several figures of data that raised these initial concerns about tool sensitivity. In addition, wording of the text has been altered to further strengthen their point that the tool detects “subtle” biological differences (for example, over developmental time or upon brief VEGF inhibition). Yet, I am not entirely convinced that these detected differences are subtle and wouldn't be easily detected using other analysis methods. Without adding new data validating that their pipeline can identify known subtle phenotypes described elsewhere, the broader utility of this tool and its improvement on other approaches are still unclear.

**Our response:**

© 2022. Published by The Company of Biologists under the terms of the Creative Commons Attribution License (https://creativecommons.org/licenses/by/4.0/). 

We agree with the reviewer on the need to show our approach has sufficient sensitivity to detect biologically meaningful changes in vascular patterning. We hope that after having revised the manuscript substantially it is sufficiently clear in allowing the reader to judge that ZVQ has this sensitivity.

As there is no “gold-standard” method in the field, sensitivity in this context can be reasonably defined as an ability to detect statistically significant differences using realistic group sizes in response to experimental conditions. This is the main purpose of the experimental data we included. This experimental data was used to demonstrate the performance of this tool in a range of experimental conditions commonly applied in the field. We deliberately chose published manipulations but in the case of the pharmacological inhibition of VEGF, Notch, Wnt etc we used far shorter durations of treatment than have been previously published to induce less severe defects. It is not surprising some of these treatments were associated with less effect than previously published studies (which will tend to show the doses and treatments that induce the greatest effect, and not neutral effects).

Our data confirm the sensitivity of our approach to detect minor alterations in vascular patterning. As an example, Figure 4A shows the population average maps (PAMs) of groups of six animals treated with 2h VEGF inhibitor or control. In the revised manuscript we explain the relevance of PAMs for the interpretation of global and local phenotypes in more depth. The VEGF inhibition PAMs show overall and regional vessel patterning is largely preserved. However, quantification reveals subtle but statistically significant differences in multiple vascular parameters (Fig. 4B-I). A similar ability to detect statistically significant quantitative differences in vascular parameters, despite very similar overall vascular patterning on the PAMs, was seen in most experimental conditions we examined. Together, this confirms that ZVQ detects differences that would not be detectable by-eye.

The reviewer suggests “other analysis methods” could potentially outperform our approach, and we would like to reemphasize that to our knowledge there is currently no (open-source) tool available for zebrafish brain vasculature segmentation and quantification. As our work is producing this first analysis workflow, we propose that the here presented approach establishes a benchmark that future analysis workflows can be compared against. As long as other methods are lacking, a comparative approach to assess outcomes is impossible and our approach was to use manual measurements and inter-approach comparisons to establish validity of our workflow before integrating all steps into ZVQ (Kugler et al. 2020 doi: 10.1101/2020.07.21.213843; Kugler et al. 2019 doi: 10.3390/jimaging5010014; Kugler et al. 2020 doi: 10.1007/978-3-030-39343-4_23).

The reviewer suggests we examine the effect of three further manipulations (cxcl12b and cxcr4a knockdown and Wnt inhibition) because these induce “subtle” effects. The figures below from published papers demonstrate that all these induce severe vascular phenotypes that are easily detectible by eye and thus would not help demonstrate that your method is able to detect subtle differences. For comparison we show the PAMs from our studies using short-term VEGF inhibition (Figure 4) which induces a far less severe phenotype. We therefore are confident that performing such studies would not be helpful in further demonstrating the sensitivity of our tools.

Together, our data does already show our approach does not “only detect severe changes in cerebrovascular architecture”.

***The effect of Wnt inhibition using IWR-1 (for 43hrs from 29-72hpf) From Hübner et al., 2018:***

***NOTE:*** *Figure provided for reviewer has been removed. It showed part of Supplementary Fig. 1a from* Hübner, K., Cabochette, P., Diéguez-Hurtado, R. et al. Wnt/ß-catenin signaling regulates VE-cadherin-mediated anastomosis of brain capillaries by counteracting S1pr1 signaling. *Nat Commun* 9, 4860 (2018). (doi: 0.1038/ s41467-018-07302-x)

***The effect of cxcl12b and cxcr4a mutation From Bussmann, Wolfe, and Siekmann, 2011:***

© 2022. Published by The Company of Biologists under the terms of the Creative Commons Attribution License (https://creativecommons.org/licenses/by/4.0/). 

**NOTE**: *Figure provided for reviewer has been removed. It showed part of Fig. 4d, e and f from* Jeroen Bussmann, Scot A. Wolfe, Arndt F. Siekmann; Arterial-venous network formation during brain vascularization involves hemodynamic regulation of chemokine signaling. *Development* 1 May 2011; 138 (9): 1717–1726. (doi: 10.1242/dev.059881)

***Population average maps of animals treated for 2h with the VEGF inhibitor AV951***

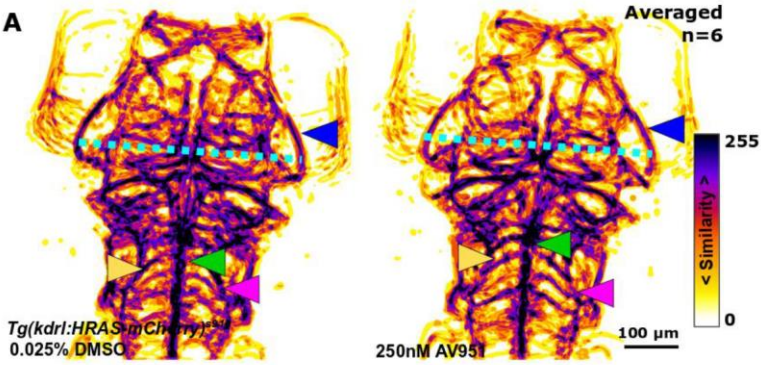


(2) Another remaining concern that has not been fully addressed is that the output of the workflow is somewhat superficial and does not generate much insight into the cause of observed defects (other than to describe global shifts in the analyzed parameters). For example, a decrease in vessel similarity is observed upon loss of dll4 expression (Fig. S13), but what this actually reflects at the level of vascular architecture remains obscure. Can this observation be traced back to manipulation-induced variance in specific regions or vessels? This is essential as a route to defining gene or pathway function, otherwise insights generated by this workflow are not particularly informative. Similarly, it is critical to understand why vascular volume and surface volume are specifically elevated upon DAPT treatment – does this indicate wider vessel lumens? And if so, in which regions? Likewise, if network length is reduced upon loss of jagged-1b, are all vessels slightly reduced in length or does this reflect a reduction in length (or loss) of specific vessels? Perhaps even more puzzling is the transient dip in vessel surface volume seen at 4dpf (Fig. 2F) and transient increase in vessel density observed at 3dpf (Fig. 2G) during normal embryonic development. This data was excluded from the original submission and added upon request, but raises a key question as to the robustness of the analysis tool. Can these unexpected shifts be explained by specific developmental stage-dependent changes to vessel shape and/or topology (which can then be clearly indicated in a representative image), or do they simply reflect noise or error in the data acquisition or analysis pipeline. Hence, rather than only providing the 8x pipeline output values, which only provide superficial insight, this needs to be followed up with a clear description of the underlying cause of this parameter shift to (1) confirm the utility of the tool as a means to generating informative insights into gene or pathway function, and (2) validate the robustness of the analysis pipeline to confirm that any unexpected shifts in output values are truly meaningful and of biological significance (i.e. not artifactual).

**Our response**:

The Population Average Maps, ZVQ quantification results, and interpretation by experts are intended to be used together for maximum insight. The PAMs allow by-eye assessment of group differences in regional vascular patterning. As we demonstrate, such regional changes can then be further quantified or compared with other regions by selecting different Region/s of Interest (exemplified by midbrain to hindbrain measurements, and left to right vasculature comparison).

Our workflow produces a comprehensive set of objectively measured parameters, creating a benchmark for cerebrovascular measurements. The relevance of each parameter will vary according to the phenotype sought or detected and the scientific question. The specific alteration in similarity the reviewer mentions reflects inter-group differences in vascular patterning in the *dll4* morphant group (i.e. not only are they different to the controls but they are more different to each other than controls are to each other). We speculate this is due to

© 2022. Published by The Company of Biologists under the terms of the Creative Commons Attribution License (https://creativecommons.org/licenses/by/4.0/). 

variable morpholino dose effects. This metric could be useful to detect incompletely penetrant phenotypes but this will depend on the research question.
Insights into gene or pathway function will come from the types of experimental groups quantified and compared using our tools, not from the tools themselves. It is impossible to design a tool that generates all possible desired parameters for all possible experimental questions, but the range of parameters generated by ZVQ is likely to be helpful to a large proportion of studies in the field, especially those in which it is not known whether an experimental manipulation has any effect on vascular patterning at all.

The reviewer suggests the pattern in vascular density during development is unexpected. We anticipate that the change from 2-to-3dpf in vascular density is likely to be due to the higher head curvature at 2dpf, rather than less volume (Fig. 2E) or head size (Fig. S3); we have included this in the discussion. Similarly, we suggest that the transient dip in surface at 4dpf might be due to **(a)** the amount of pruning starting at 3dpf resulting in decreased surface area, and **(b)** the fact that myogenic responses and vascular regulation start at 4dpf and might alter surface transiently. Again, as there is no benchmark to compare our data against, future work is needed and we now discuss this in the paper.

Minor points:

(1) I previously commented that the statement in the text suggesting that image registration improves the “reproducibility of quantification” was not experimentally explored. However, the authors claim that they could not find this section, despite it still being included in the text (now on page 7). This statement needs to be reworded.
**Our response:** We corrected this sentence.

(2) Several references to Figures in the main text refer to the incorrect figure – for example references to Fig. S2C-G and Fig. 3D on page 6, and Fig. 4A on page 8.
**Our response:** We corrected this.

(3) Figure S3, S5 and S16 are not referred to or described in the main text.
**Our response:** We changed figure S3 to be S1; found S5 to be missing/mislabelled, which changed the numbering of the subsequent supplementary figures; the lack of referral to S16 – now S15 – has been corrected.

***Reviewer 2 Advance Summary and Potential Significance to Field:***

The authors have developed an image analysis pipeline for the alignment and analysis of brain vasculature in 3D for larval zebrafish. The pipeline supports alignment and quantification of several vasculature parameters for whole brain datasets in larval zebrafish. This could be a potentially useful tool for many labs in the field, and would require little computational expertise. The tool development addresses a gap in the field for statistically driven analysis to map changes in vasculature between different samples/genotypes.

Reviewer 2 Comments for the Author:
The authors have addressed/clarified most of the comments from the previous review. I do appreciate that some fundamental/conceptual challenges persist and would be hard to address within the scope of this manuscript/project. I am satisfied with the manuscript in its current form and would be happy to proceed with publication. The authors have also made an effort to ensure that their pipeline/test datasets are more openly accessible to the community.
I do however strongly suggest/recommend that the authors in the future develop standardized reference brains and explore elastic/diffeomorphic registration approaches and explore robust parameters for true 3D analysis of their datasets. That will render their approach much more powerful than their current pipeline for a robust analysis of brain vasculature.
That said, the current tool/analysis pipeline should benefit researchers in the community for a preliminary analysis of their 3D datasets without requiring extensive computational training.

© 2022. Published by The Company of Biologists under the terms of the Creative Commons Attribution License (https://creativecommons.org/licenses/by/4.0/).

**Our response:** We are grateful to the reviewer for their time and consideration of this manuscript. We agree that future work, as suggested, should include standardized reference brains and vessel-specific analysis – particularly across ages, and have expanded the discussion on this in the revised manuscript.

---

Second decision letter

MS ID#: DEVELOP/2021/199720

MS TITLE: Zebrafish Vascular Quantification (ZVQ): a tool for quantification of three-dimensional zebrafish cerebrovascular architecture by automated image analysis

AUTHORS: Elisabeth Christina Kugler, James Frost, Vishmi Silva, Karen Plant, Karishma Chhabria, Tim Chico, and Paul Armitage
ARTICLE TYPE: Research Article

I sent your manuscript back to one reviewer who is happy with your revisions and so I am happy to tell you that your manuscript has been accepted for publication in Development, pending our standard ethics checks. The referee's report on this version is appended below.

Reviewer 1

*Advance summary and potential significance to field*

The manuscript by Kugler et. al. describes a new computational pipeline for the 3D analysis of cerebrovascular architecture in zebrafish larvae that will be a useful resource for others in the vascular field.

*Comments for the author*

In revising this manuscript, the authors have added significant text additions and discussion points that have toned down several claims that were not entirely supported by the data and have addressed most of my previous points of concern.

© 2022. Published by The Company of Biologists under the terms of the Creative Commons Attribution License (https://creativecommons.org/licenses/by/4.0/).
